# Is Japan’s child allowance effective for the well-being of children? A statistical evaluation using data from K-CHILD study

**DOI:** 10.1186/s12889-020-09367-0

**Published:** 2020-10-06

**Authors:** Yuna Koyama, Takeo Fujiwara, Aya Isumi, Satomi Doi

**Affiliations:** Department of Global Health Promotion, Tokyo Medical and Dental University (TMDU), 1-5-45 Yushima, Bunkyo-ku, Tokyo, 113-8510 Japan

**Keywords:** Child allowance, Cash transfer, Household spending, Parental investment, Child health, Behavior problems, Propensity score matching

## Abstract

**Background:**

Child allowance payment is one form of social security policy that aims to mitigate the child poverty gap by providing financial support to families. This study aimed to explore the impact of the child allowance on children’s physical and mental health (BMI, problem behavior, depression, and self-rated health), and parental investment in child health (children’s material goods, family events, extracurricular activities, interaction with children, and involvement in child maltreatment).

**Methods:**

We used cross-sectional data from the 2016 Kochi Child Health Impact of Living Difficulty (K-CHILD) study. Participants were 1st, 5th and 8th grade children living in Kochi prefecture in Japan (*N* = 8207). Caregivers reported children’s child allowance status, BMI and behavior problems, while children filled out a self-assessment on depression and health condition. Propensity score matching analysis regarding potential confounders was used.

**Results:**

We found that children in families that received child allowance showed a smaller total difficulties score by 1.29 points (95% CI: − 2.32 to − 0.25) and a lower risk of overweight (OR: 0.51, 95% CI: 0.29 to 0.91) although there is no association with underweight, prosocial behavior, depressive symptoms and self-rated health. Parental investment did not differ by child allowance status (*p* > 0.05).

**Conclusions:**

Child allowance was found to be potentially beneficial in decreasing behavior problems and reducing child overweight. Further longitudinal studies are needed to elucidate how child allowance is used by family members and associated with children’s well-being.

(230/350 words)

## Background

Child poverty is a growing problem around the world. In 2016, the prevalence of children living in poverty was 17.0% in 89 countries [1] and 13.1% among OECD countries [2]. The rates have increased in more than half of OECD countries since the early 1990s [3], which is partially due to the long-term changes in demographics and labour market behavior, or insufficient development of policies tackling poverty [4, 5]. It is also a growing problem in Japan, where the child poverty rate was 13.9% in 2015 [6, 7], which is relatively higher compared to the average rate of OECD countries. Since childhood is a critical period for human development, the impact of growing up in poverty affects children throughout their life course. Children living in poverty are less likely to perform well at school, to have good health or life satisfaction, and to realize their full potential [8]. Combatting child poverty, therefore, is an increasingly important public health issue.

To tackle child poverty, several social policies are implemented. Among the policies, cash transfer, where money is given to households directly, is believed to be more beneficial [9] than in-kind benefit because it allows more flexible money allocation within families to meet the needs of each household and the children [10]. Although some studies have shown that unconditional cash transfer programs do not impact on the total health service usage among children and adults [11, 12], nor on inequalities in household income and child’s health status [13], the programs were also shown to improve some health determinants [11, 14–16], health service access [17, 18], and health outcomes [15, 17] in low- and middle-income countries. Comparing to low- and middle-income countries, the number of studies in high-income countries is small, especially in medical settings. A randomized controlled trial in Australia showed the effectiveness of conditional cash transfer on vaccination uptake [19], while health outcomes in high-income countries were not conclusive [20, 21].

Child allowance, a cash transfer program targeting households with children, is thought to improve children’s outcomes through two mechanisms. One is the direct impact of increased investment in children. This pathway is called the ‘resource channel’ [22, 23]. Several studies proved that child allowance payments increased household spending on children [22, 24], such as on education and basic expenditure, and reduced risky consumption of alcohol or tobacco [22]. The psychological mechanism underlying this favorable money allocation is known as the ‘labeling effect’, in that parents experience a moral obligation to spend a relatively large part of their child allowance on their children [24]. This phenomenon is partially influenced by peer pressure, thus we hypothesized its impact might differ depending on the country where the policy is implemented. Therefore the impact should be considered in each social context although this has not been done universally so far. In addition, in spite of the theoretical hypothesis that resource channel is beneficial for children, there are still the limited number of studies exploring what kind of investment could produce benefits for children, and only investing in early childhood education and care [25], or extracurricular activities [26] have been shown to be beneficial. Thus to date, there is no empirical evidence showing that child allowance may have an impact on child health and well-being through resource channel.

The second pathway, known as the ‘family process channel’, involves improving the quality and quantity of parent-child interactions and, as a result, family function [22, 23]. It is well known that poverty badly affects child development [27–31] through poor parental cognitive function and economic decision making [32] and low quality of parenting [33] due to parental mental stress [33]. In addition, unpredictable condition due to unstable financial status [30] inhibits child healthy development because of family conflicts and increased parental working time induced by economic pressure [34]. Direct cash transfer could mitigate these negative factors by providing a fixed income. Though the importance of child-parental interaction and family social capital on a child’s well-being is established [35, 36], this process is often overlooked when assessing the impact of child allowance payments.

Not only the mechanism but also the effectiveness of child allowance payments on children’s well-being itself has not been widely researched. Some studies have shown that child allowance can increase favorable health behaviors such as vaccination [37], and improve physical and mental health for both children and mothers [23]. The evidence on academic outcomes is controversial [23, 38]. As for mitigating child poverty, recent research has shown that both the selective system targeting poor children and the universal system, which often holds a higher distributive budget, are strongly associated with higher levels of child poverty reduction [30, 39, 40]. Russian research, however, has demonstrated that more poverty reduction can be observed in the universal system rather than in a targeted system [41], and a study in Canada showed more investment in education among low-educated families under the universal system [22]. In addition, universal distribution helps to eliminate the discrimination or stigma associated with severe poverty among people who have no option but to turn to a social safety net [30].

A child allowance system has been also implemented as a social security policy for children in Japan. Under the national system, caregivers or parents who raise children aged 0 to 15 [42] can receive payments, or a child allowance (*jido teate*). The amount of payment is determined based on the number and ages of children, and household income (i,e. means test). For example, if household income is below the threshold, families with children aged 0 to 3 years old are eligible to receive 15,000 JPY per month and 10,000 JPY per month is given if children are aged 3 to 15 years old. Under the current system, even caregivers whose household income exceeds the threshold can receive a special benefit of 5,000 JPY per month (*tokurei kyufu*), making the Japanese child allowance system  a universal system that is fairly accessible to most families (for more details of Japanese child allowance system, see Appendix).

Previous Japanese studies showed controversial results on money allocation within families. Some showed that receiving the child allowance resulted in increased expenditure on children, such as education and insurance [43]. On the other hand, others revealed that most of the allowance was saved [44] and expenditure on children did not change [45]. Moreover, to the best of our knowledge, no previous study has explored whether child allowance improves child health or well-being in Japan, making it problematic to enforce an effective child allowance policy from a public health perspective. In addition, the Japanese sample may have a unique contribution in assessing the impact of child allowance on child well-being. This is because under the current system, potential recipients must submit application forms to each municipality in order to receive the allowance, which makes the proportion of recipients among eligible children lower than expected, that is, 92% in 2017 [46], and this enables us to evaluate its impact by comparing recipients and non-recipients with similar backgrounds. In addition, studies investigating the impact of child allowance on child well-being, including mental health are scarce. Thus, this study aimed to examine the impact of child allowance on child well-being, and parental investment in their children.

## Methods

### Data and sampling methods

**Fig. 1:**
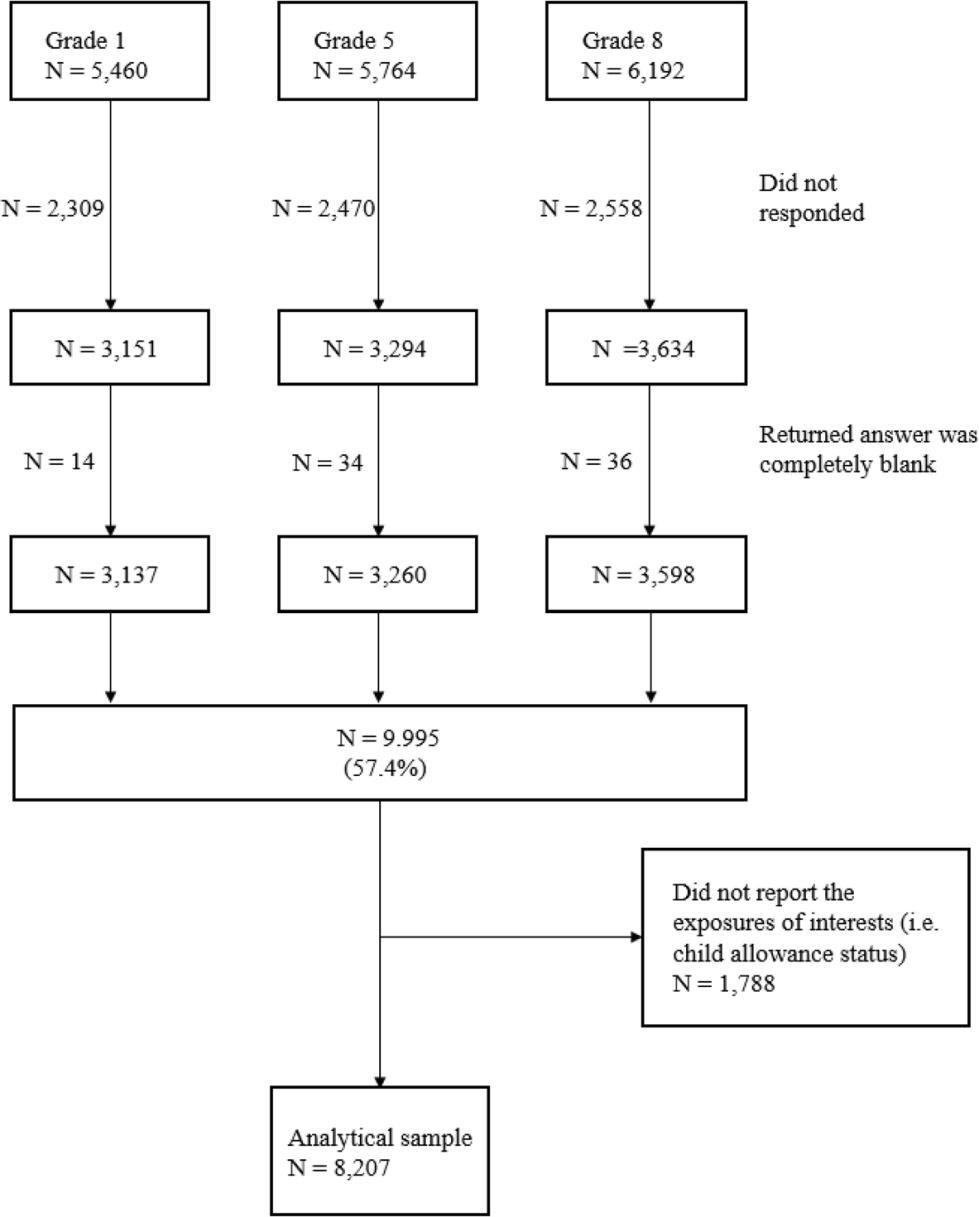
Sampling flow chart

The current study was secondary analysis of the Kochi Child Health Impact of Living Difficulty (K-CHILD) study, the cross-sectional study conducted in 2016, which is described elsewhere in detail [47]. The K-CHILD study aimed to examine the living environment and health of children in all public, private and special needs schools, except for correspondence course high schools and one special needs school in Kochi prefecture, Japan. Self-reported questionnaires (targeting both children and caregivers, whose contents depended on variables and grade, and developed originally, see supplementary file) were distributed to all the students enrolling in 1st, 5th and 8th school grades in Kochi prefectures; 5460 children in 1st grade, 5764 children in 5th grade, and 6192 children in 8th grade, and totaled 17,416 children and their caregivers. For the 1st graders, the questionnaires were filled out by caregivers. A total of 10,079 questionnaires were returned via mail in Kochi city (*N* = 3417, response rate = 38.9%) and via school in other cities except for Kochi city (*N* = 6662, response rate = 77.2%). Of these, 9995 responses were valid (response rate 57.4%). Considering the difference in rates between cities due to different data collection methods, we weighted with inverse response rates. We excluded the samples with questionnaires not indicating child allowance status. Finally, 8207 questionnaires were analyzed (2466 dyads, 2735 dyads, 3006 dyads, for 1st, 5th, and 8th grade, respectively) (Fig. 1).

### Measurements

#### Child allowance

Child allowance status was assessed by asking parents how much they receive as child allowance. Because we are interested in finding out whether caregivers are receiving child allowance or not, we dichotomized the responses if caregivers answered with a value more than ‘0 JPY’ (i.e. receiving) or ‘0 JPY’ (i.e. not receiving).

#### Child health outcomes

Children’s body mass index (BMI) was calculated with self-reported heights and weights obtained from children themselves (for 8th graders) and caregivers (for 1st and 5th graders). BMI was expressed as z-scores representing the deviation in standard deviation units from the mean of a standard normal distribution of BMI specific to age and sex according to the WHO Child Growth Standards [48]. We categorized BMI as underweight (<−1SD), normal weight (−1SD to 1SD) and overweight (+1SD).

Problem behavior was assessed by caregivers using the Japanese version of the Strengths and Difficulties Questionnaire (SDQ) [49], which was generated from the original version [50]. The SDQ scores were calculated using the scale of total difficulties scores and prosocial behavior scores. The Cronbach’s alpha for total difficulties score and prosocial behavior were 0.81 and 0.72 in the current study, respectively.

Child depressive symptoms were assessed by children (5th and 8th graders) using the Japanese version of the Depression Self-Rating Scale (DSRS), validated previously [51]. In the current study, children answered a total of 15 items with the scale from “0 = never” to “2 = most of the time”, and we calculated the sum of the rated values (Cronbach’s alpha = 0.84). A higher score indicates children have more depressive symptoms.

Child’s self-rated health (SRH) was assessed among 5th and 8th grade children. Responses were selected from a five-point Likert scale (1 = not good; 2 = not very good; 3 = normal; 4 = somewhat good; 5 = good). Child’s SRH was confirmed as an indicator of physical and psychological health [52].

#### Financial investment for children (resource channel)

Caregivers were asked about their experience that they could not afford to have materials for children in their home and family events due to financial reasons. For investment in extracurricular activities, caregivers were asked how much they pay for them. The sum of the fees was calculated and categorized as “0 JPY”, “1–10,000 JPY”, “10,001–20,000 JPY”, “20,001–30,000 JPY”, and “+ 30,001 JPY” (see supplementary method 1 for details).

#### Time investment for children and maltreatment (family process channel)

Time investment was assessed by the frequency of caregivers’ interaction with children and child maltreatment experience to account for both quantity and quality of investment. Child maltreatment was assessed by parental response to 8 items on child maltreatment, such as beating or kicking [53, 54] Caregivers who reported no maltreatment were coded as “1 = no maltreatment”, otherwise “0 = maltreatment” (see supplementary method 1 for details).

#### Covariates

Potential confounders of the association between child allowance and child health outcomes, and predictive variables of child health outcomes were chosen as covariates. Caregivers were family size, number of younger and older siblings, number of cohabiting grandparents, marital status, place of residence, parental ages, parental educational attainments, parental occupational status, parental working times, household income from employment, respondent’s K6 score [55, 56] as parental psychological distress, respondent’s self-rated health, parental smoking habits, child’s sex and child’s grade (Table 1). These variables were used for prediction of propensity scores for child allowance status (see supplementary method 1 for details).

**Table 1:** Description of characteristics of family with/without child allowance before and after propensity score matching

Variables	Before matching						After matching					
	Child allowance (−) *N* = 219		Child allowance (+) *N* = 7988		*P*-value	%bias	Child allowance (−) *N* = 217		Child allowance (+) N = 217		P-value	%bias
	N/Mean/Median	%/SD/IQR	N/Mean/Median	%/SD/IQR			N/Mean/Median	%/SD/IQR	N/Mean/Median	%/SD/IQR		
Respondents												
Mother	184	84.0	7144	89.43			182	83.9	176	81.1		
Father	27	12.3	727	9.10		**−10.4**	27	12.4	31	14.3		6.0
Others	7	3.2	64	0.80		**−17.1**	7	3.2	9	4.2		6.6
Missing	1	0.5	53	0.66	**0.004**	2.8	1	0.5	1	0.5	0.872	0.0
Family size	4	1	4	1	0.568	7.6	4	1	4	1	0.676	5.6
Number of younger siblings	0	1	0	1	**< 0.001**	**29.7**	0	1	0	1	0.335	−5.9
Number of older siblings	1	1	0	1	**0.047**	**−16.0**	1	1	1	1	0.110	**15.0**
Number of grandparents	0	0	0	0	0.110	−7.6	0	0	0	0	0.711	0.0
Marital status												
Married	181	82.7	6771	84.76			179	82.5	175	80.7		
Divorced	33	15.1	1005	12.58		−7.2	33	15.2	36	16.6		4.0
Widowed	2	0.9	74	0.93		0.1	2	0.9	3	1.4		4.8
Unmarried	3	1.4	82	1.03		−3.2	3	1.4	3	1.4		0.0
Missing	0	0.0	56	0.70	0.568	**11.9**	0	0.0	0	0.0	0.940	0.0
Residential place												
Prefectural capital city	100	45.7	2986	37.38			99	45.6	83	38.3		
Within prefecture	117	53.4	4920	61.59		**16.6**	116	53.5	134	61.8		**16.8**
Outside of prefecture	2	0.9	30	0.38		−6.7	2	0.9	0	0.0		**−11.5**
Missing	0	0.0	52	0.65	**0.024**	**11.4**	0	0.0	0	0.0	0.080	0.0
Mother’s age	43.6	5.0	41	5.16	**<0.001**	**−48.0**	43.6	5.0	44.6	4.6	**0.026**	**20.2**
Father’s age	46.4	5.6	43	5.67	**<0.001**	**−58.6**	46.3	5.6	47.3	5.3	**0.049**	**18.4**
Mother’s education												
High school or lower	52	23.7	2688	33.65			52	24.0	47	21.7		
Technical/ junior college/ university dropout	96	43.8	3583	44.85		2.0	95	43.8	91	41.9		−3.7
University or higher	55	25.1	1327	16.61		**−21.0**	54	24.9	60	27.7		6.8
Missing/ unknown	16	7.3	390	4.88	**<0.001**	**−10.1**	16	7.4	19	8.8	0.823	5.8
Father’s education												
High school or lower	53	24.2	3328	41.66			53	24.4	36	16.6		
Technical/ junior college/ university dropout	30	13.7	1517	18.99		**14.3**	30	13.8	20	9.2		**−12.5**
University or higher	103	47.0	1945	24.35		**−48.7**	101	46.5	131	60.4		**29.7**
Missing/ unknown	33	15.1	1198	15.00	**<0.001**	−0.2	33	15.2	30	13.8	**0.026**	−3.9
Mother’s occupation												
Full-time employee	77	35.2	2782	34.83			76	35.0	86	39.6		
Part-time/ temporary employee	62	28.3	2919	36.54		**17.6**	62	28.6	51	23.5		**−10.9**
Self-employed/ others	23	10.5	888	11.12		2.0	23	10.6	14	6.5		**−13.3**
Not working	41	18.7	1055	13.21		**−15.1**	40	18.4	44	20.3		5.0
Missing	16	7.3	344	4.31	**0.010**	**−12.8**	16	7.4	22	10.1	0.286	**11.8**
Father’s occupation												
Full-time employee	149	68.0	5070	63.47			147	67.7	149	68.7		
Part-time/ temporary employee	2	0.9	304	3.81		**19.1**	2	0.9	1	0.5		−3.0
Self-employed/ others	33	15.1	1364	17.08		5.5	33	15.2	28	12.9		−6.3
Not working	1	0.5	75	0.94		5.8	1	0.5	0	0.0		−5.5
Missing	34	15.5	1175	14.71	0.125	−2.3	34	15.7	39	18.0	0.767	6.4
Mother’s working time	36.3	11.7	6	11.46	0.987	0.1	36.2	11.7	37.5	10.0	0.219	**11.1**
Father’s working time	48.7	14.4	48	11.48	0.288	−6.5	48.4	14.0	48.8	9.4	0.767	2.6
Income from employment (million JPY)												
< 3.00	34	15.5	1585	19.84			34	15.7	34	15.7		
3.00–5.99	44	20.1	2918	36.53		**37.1**	44	20.3	37	17.1		−7.3
6.00–9.99	36	16.4	1831	22.92		**16.3**	36	16.6	43	19.8		8.1
≧ 10.00	66	30.1	660	8.26		**−57.7**	64	29.5	71	32.7		8.5
Missing	39	17.8	994	12.44	**<0.001**	**−15.0**	39	18.0	32	14.8	0.685	−9.0
Respondent’s K6 score	1	4	2	5	**0.027**	**15.1**	1	4	2	4	0.619	4.5
Parental self-rated health												
Excellent	73	33.3	2515	31.5			73	33.6	74	34.1		
Very good	75	34.3	2569	32.2		−4.4	75	34.6	67	30.9		−7.8
Good	45	20.6	1987	24.9		**10.3**	45	20.7	48	22.1		3.3
Fair	15	6.9	593	7.4		2.2	15	6.9	17	7.8		3.6
Poor	4	1.8	52	0.7		**−10.6**	2	0.9	4	1.8		8.3
Missing	7	3.2	272	3.4	0.270	1.2	7	3.2	7	3.2	0.937	0.0
Maternal smoking habit												
Current smoker	19	8.7	1084	13.6			19	8.8	12	5.5		
Ex-smoker	24	11.0	1332	16.7		**16.6**	24	11.1	15	6.9		**−12.0**
Never	156	71.2	5196	65.1		**−13.3**	154	71.0	167	77.0		**12.9**
Missing	20	9.1	376	4.7	**< 0.001**	**−17.5**	20	9.2	23	10.6	0.222	5.5
Paternal smoking habit												
Current smoker	52	23.7	2824	35.4			52	24.0	42	19.4		
Ex-smoker	62	28.3	2144	26.8		−3.3	61	28.1	70	32.3		9.3
Never	70	32.0	1838	23.0		**−20.1**	69	31.8	69	31.8		0.0
Missing	35	16.0	1182	14.8	**0.001**	−3.3	35	16.1	36	16.6	0.638	1.3
Child’s sex												
Boys	102	46.6	3781	47.3			101	46.5	98	45.2		
Girls	109	49.8	4029	50.4		1.3	108	49.8	105	48.4		−2.8
Missing	8	3.7	178	2.2	0.348	−8.4	8	3.7	14	6.5	0.443	**16.4**
Child’s grade												
1st	38	17.4	2428	30.4			38	17.5	29	13.4		
5th	64	29.2	2671	33.4		9.1	64	29.5	62	28.6		−2.0
8th	117	53.4	2889	36.2	**<0.001**	**−35.2**	115	53.0	126	58.1	0.418	**10.3**

Bold signified *p* < 0.05 and |% bias| > 0.10

N, % and p-value for chi-square test were provided for parental education, maternal occupation, income, parental smoking status, and child grade

N, % and p-value for Fisher’s exact test were provided for respondents, marital status, residential place, paternal occupation, parental self-rated health, and child sex

Mean, SD and *p*-value for t-test were provided for parental age, and parental working time

Median, IQR and *p*-value for Wilcoxon rank-sum test were provided for family size, number of young siblings, number of old siblings, number of grandparents, and K6 score

### Analysis and estimation methods

Propensity score matching analysis was used to conduct a quasi-experimental study by comparing child health in recipient and non-recipient households with similar backgrounds. Propensity score matching allows to exclude bias regarding exposure allocation before accessing outcome, which makes observational study more analogous to experimental design, and to include as much covariates as possible regardless of sample size [57, 58]. Ideally, we need panel data on allowance recipients and non-recipients to reliably study the impact. Observational impact assessment studies have analyzed such data using a variety of methods such as double difference, propensity score matching or both [59]. But our analysis is based on only post-intervention data. Therefore, we could only employ a multivariate regression analysis and propensity score matching methods for impact assessment. Propensity scores were calculated using multiple logistic regression with the potential predictive variables stated above (see details of propensity score estimation and matching in supplementary method 2). The histogram of propensity scores are shown in supplementary figure 1. The missing values for categorical and numerical data were substituted with dummy variables and median values, respectively (for the missingness, see supplementary table 1). Subjects were matched based on the obtained scores using 1-to-1 (one treated matched to one control) optimal matching with caliper width equal to 0.01 to avoid making poorly matched pairs. Matching was done with no replacement since there are enough overlapping regions of distribution of propensity score in treated and untreated groups [60]. The balance of possible confounders within the matched pairs was checked using standardized bias, which was calculated as the mean difference of non-recipients and recipients as a percentage of the average standard deviation of non-recipients and recipients (Table 1). Propensity score matching was done with command “psmatch2”. Finally, the association between child allowance status and child’s health outcomes were analyzed with conditional logistic regression for BMI and linear fixed effects regression for behavior problems, prosocial problems, depressive symptoms and self-rated health, using the matched pairs. Also, differences in financial and time investment between recipients and non-recipients were assessed with chi-square test for categorical variables and t-test for continuous variables. In order to confirm sensitivity of the estimate, we conducted the sensitivity analysis using multiple imputation for missing in covariates (supplementary method 2). All analyses were performed with STATA 15.0.

## Results

Table 1 describes the characteristics of samples with and without child allowance before and after propensity score matching. As for the recipient families, the median numbers of younger and older siblings were both 0, 36.5% of families earned JPY 3,000,000 to 5,999,999 per year, 47.3% of children were boys, and 30.4, 33.4, and 36.2% were 1st, 5th, and 8th grade children, respectively. In families without child allowance, less younger siblings and more older siblings were cohabiting (*p* < 0.001 and *p* = 0.047, respectively), more lived in capital city (53.4% vs 61.6%, *p* = 0.024), both mothers and fathers were older (43.6yo vs 41.2yo, p < 0.001 and 46.4yo vs 43.1yo, p < 0.001), parents had completed higher education (p < 0.001), total wages for employment was relatively high or low (p < 0.001), less parents suffered from mental health problems (*p* = 0.027), and parents smoked less (p < 0.001).

Table 2 shows the association between child allowance status and child health outcomes. The inverse association of child allowance status with total difficulties score (coefficient = − 1.29, 95% CI = − 2.32 to − 0.25) and reduction of overweight (odds ratio = 0.51, 95% CI = 0.29 to 0.91) were observed. There was no association with underweight, prosocial behavior, depressive symptoms and self-rated health.

**Table 2:** The coefficients of the association between child allowance status and child health before and after matching

Variables	Before matching							After matching			
		Unadjusted			Adjusted						
	N	OR	95%CI	P-value	OR	95%CI	P-value	N	OR	95%CI	P-value
Physical health											
Body mass index	5595							124			
Underweight		1.03	0.53 to 2.00	0.928	0.91	0.46 to 1.80	0.788		0.70	0.25 to 1.99	0.501
Normal weight		(Ref.)			(Ref.)				(Ref.)		
Overweight		0.67	0.48 to 0.94	**0.020**	0.62	0.44 to 0.87	**0.006**		0.51	0.29 to 0.91	**0.024**
Mental health	N	B	95%CI	P-value	B	95%CI	P-value	N	B	95%CI	P-value
SDQ total difficulties score	8102	−0.07	−0.79 to 0.66	0.857	−0.93	−0.61 to −0.27	**0.006**	433	−1.29	−2.32 to −0.25	**0.015**
SDQ prosocial score	8108	−0.02	−0.29 to 0.26	0.900	− 0.01	− 0.28 to 0.27	0.957	434	−0.08	− 0.50 to 0.34	0.695
Depression	5628	− 0.24	− 0.84 to 0.36	0.432	− 0.16	− 0.76 to 0.43	0.586	365	0.33	−0.46 to 1.11	0.414
Self-rated health	5616	−0.03	−0.18 to 0.12	0.697	0.00	−0.15 to 0.16	0.977	360	0.21	−0.01 to 0.42	0.066

Adjusted model is adjusted for family size, number of younger and older siblings, number of cohabiting grandparents, marital status, place of residence, parental ages, parental educational attainments, parental occupational status, parental working times, household income from employment, respondent’s K6 score, respondent’s self-rated health, parental smoking habits, child’s sex and child’s grade

Bold signified p < 0.05

Depression and self-rated health score are not available for 1st grade children

Table 3 shows the comparison of household investment between child allowance non-recipients and recipients. 14.9% of recipients lacked the goods for child, 24.9% did not hold family events, 29.1% payed 0 to 9999 JPY for child extracurricular activities and 27.5% maltreated child. Mean parent-child positive interaction score was 21.7 and 20.9 for non-recipients and recipients respectively. There were no differences in household investments. Further sensitivity analysis applying multiple imputation showed similar results (supplementary table 2).

**Table 3:** The description of household expenditure of child allowance non-recipients and recipients before and after matching

Variables	Before matching					After matching				
	Child allowance (−) N = 219		Child allowance (+) N = 7988		P-value	Child allowance (−) N = 217		Child allowance (+) N = 217		P-value
	N / Mean	% / SD	N / Mean	% / SD		N / Mean	% / SD	N / Mean	% / SD	
Financial investment										
Goods for child										
Yes	186	86.5	6909	88.4		184	86.4	177	85.1	
No	29	13.5	911	11.7	0.408	29	13.6	31	14.9	0.705
Family events										
Yes	179	81.7	5436	68.1		177	81.6	163	75.1	
No	40	18.3	2552	32.0	**< 0.001**	40	18.4	54	24.9	0.103
Extracurricular activities										
0 JPY	35	16.7	1547	20.2		35	16.8	33	15.7	
0–9999 JPY	65	31.0	2956	38.7		64	30.8	61	29.1	
10,000–19,999 JPY	42	20.0	1633	21.4		42	20.2	50	23.8	
20,000–29,999 JPY	28	13.3	839	11.0		27	13.0	37	17.6	
≧ 30,000 JPY	40	19.1	667	8.7	**< 0.001**	40	19.2	29	13.8	0.388
Time investment										
Parent-child positive interaction score (1-45)										
	21.66	4.9	22.32	5.0	0.060	21.70	4.9	20.86	4.8	0.079
No maltreatment										
Yes	157	75.1	5057	66.1		155	74.9	153	72.5	
No	52	24.9	2592	33.9	**0.007**	52	25.1	58	27.5	0.583

Bold signified *p* < 0.050

## Discussion

The current study explored the impact of child allowance on children’s physical and mental health (i.e., BMI, behavior problems, depression, and self-rated health), and parental investment in their children (i.e., financial investment such as material goods for children, family events, and extracurricular activities, and time investment such as interaction with children, and involvement in child maltreatment). We found that children showed less behavior problems and overweight, and they tended to rate themselves heathier, possibly due to receiving the child allowance. However, no impacts of the child allowance on household investments were observed.

Our study showed that child allowance payments might improve child behavior problems and self-rated health, which was consistent with results from a previous study in Canada showing the reduction in behavior problems, especially conduct and hyperactivity problems, and improvement in self-rated general health [23]. Also, the current study showed the association between child allowance status and child overweight, which was in accordance with the previous findings [61] although we could not find any link with underweight. The previous study revealed that determinants of behavior problems were household socioeconomic status, child characteristics, family characteristics such as parental psychiatric disorder and home environment quality [62]. Self-rated health in childhood was related to low family support, missing meals and fewer outdoor activities [63]. Those studies supported the theoretical hypothesis of the resource channel and the family process channel. However, we could not observe any increase in parental investment in children. One possible reason is that we could not capture the variation such as an increase in the number of child goods or family events or quality of investment, for example, more expensive materials or family trips. Also, due to the difficulties in assessing mental distress related to family financial issues, we could not fully examine how the family process changed. Behavior problems at school was related to academic achievements [64], future earnings [65] and adult health [66], and self-rated health was shown to predict future health [67], mortality [68] and quality of life [67]. Child overweight is one of the serious global problems. Therefore, although we could not demonstrate the mechanisms hypothesized as resource channel and family process channel, our findings on the relationship of child allowance with behavior problems, self-rated health and overweight may be important from a child health perspective. Understanding how child allowance affects child health is beneficial to developing more effective policy within a limited budget. Thus, future longitudinal studies and more thorough assessments of household investment and child health are warranted.

The current study has several limitations. First, the response rate in Kochi city was relatively low due to the use of postal mail. Although we weighted the response rates, sampling bias might exist. Second, since the targeted community was based in Kochi prefecture, where the commodity price is a little lower [69], and the proportion of elderly people is increasing [70], the population characteristics might not be generalizable to all Japanese. In addition, since propensity score matching selects the population to be analyzed, there might be selection bias resulting in affecting generalizability as well. Third, the exposure of interest—child allowance status—might be misclassified because it was self-reported. However, our data showed a reasonable take-up rate. The official percentage of families receiving child allowance in Japan was 92% [46]. Additionally, thanks to a special benefit (*tokurei kyufu*), we could expect that the child allowance take-up rate would lie between 92 and 100%. Our data showed that 97.3% of the population received child allowance, which was reliable. In addition, we considered that those who declared any allowance income were recipients of the allowance. Although this dichotomization reduced the quantity of information, considering the situation that child allowance status is often under-reported [71] and there is no reason to believe that a person would declare to be a recipient when they are not (though the contrary is possible [72]), our dichotomization is justified. Even if child allowance status is underreported, our results were underestimated and we could expect more benefits on child health. Fourth, there might be a potential misspecification in model for propensity score due to unobservable confounders, which might lead to biased estimates of propensity score and prevent from assessing the association between child allowance status and child health properly. Fifth, since the current study was cross-sectional in design, it was possible some findings were due to reverse causation, e.g., parents who have children with a good sense of well-being may spend more money on their children from the child allowance. In addition, considering evaluation studies with the experimental design in developing countries, future studies employing longitudinal design, including pre- and post-intervention periods at best, are warranted. Sixth, outcome measurements were self-reported, which may have systematic measurement error due to social desirability bias [73] or psychological distress among child allowance recipients, although we adjusted potential covariates at most, such as mental health of caregivers. Further research assessing the well-being of children by teachers, school psychologists, pediatricians, or psychiatrists is needed.

## Conclusions

This study revealed that child allowance recipients reported fewer child behavior problems, and fewer children in recipient households were over-weighted. Although we could not find any differences in parental investments in children between recipients and non-recipients, it may provide a useful rationale on the effects of child allowance on child health and well-being since child allowance might be beneficial in decreasing child behavior problems and improving child physical health. Further follow-up studies and longitudinal studies are needed to assess a wider range of child health outcomes and child allowance usage, and to show the effectiveness of the allowance on child health and well-being.

## Supplementary information

**Additional file 1: Table S1.** The number of missing data. **Table S2.** The results of sensitivity analysis. **Method 1**. Measurements. **Method 2**. Sensitivity analysis. **Figure S1.** Histogram of propensity scores for recipients and non-recipients. **Appendix 1**. Child allowance system in Japan.

**Additional file 2.** Questionnaire.

## Data Availability

The datasets analyzed during the current study are not publicly available since it is the part of population-based study, i.e., Kochi Child Health Impact of Living Difficulty (K-CHILD) study, conducted by corresponding author, but are available from the corresponding author on reasonable request.

## Abbreviations

BMI: Body mass index
SDQ: Strengths and difficulties questionnaire
DSRS: Depression self-rating scale
SRH: Self-rated health
