# Supplementary material for: Is Japan’s child allowance effective for the well-being of children? A statistical evaluation using data from K-CHILD study
Source: BMC Public Health. 2020 Oct 6;20:1503. doi: 10.1186/s12889-020-09367-0 (PMC7542372; doi:10.1186/s12889-020-09367-0)
Supplement: Supplementary file 1 — Additional file 1: Table S1. The number of missing data. Table S2. The results of sensitivity analysis. Method 1. Measurements. Method 2. Sensitivity analysis. Figure S1. Histogram of propensity scores for recipients and non-recipients. Appendix 1. Child allowance system in Japan. [file 12889_2020_9367_MOESM1_ESM.docx]

Supplementary table 1. The number of missing data

|  | Amount of missing | |
| --- | --- | --- |
|  | N | % |
| Respondents | 54 | 0.7 |
| Family size | 44 | 0.5 |
| Number of young siblings | 0 | 0.0 |
| Number of old siblings | 0 | 0.0 |
| Number of grandparents | 0 | 0.0 |
| Marital status | 56 | 0.7 |
| Residential place | 52 | 0.6 |
| Mother's age | 376 | 4.6 |
| Father's age | 1232 | 15.0 |
| Mother's education | 406 | 5.0 |
| Father's education | 1231 | 15.0 |
| Mother's occupation | 360 | 4.4 |
| Father's occupation | 1209 | 14.7 |
| Mother's working time | 1519 | 18.5 |
| Father's working time | 1491 | 18.2 |
| Income from employment | 1033 | 12.6 |
| Respondent's K6 score | 178 | 2.2 |
| Parental self-rated health | 279 | 3.4 |
| Maternal smoking habit | 396 | 4.8 |
| Paternal smoking habit | 1217 | 14.8 |
| Child's sex | 186 | 2.3 |
| Child's grade | 0 | 0.0 |
| Financial investment |  |  |
| Goods for child | 172 | 2.1 |
| Family events | 0 | 0.0 |
| Extracurricular activities | 355 | 4.3 |
| Time investment |  |  |
| Parent-child positive interaction score | 327 | 4.0 |
| No maltreatment | 349 | 4.3 |

Supplementary table 2. The results of sensitivity analysis

| Variables | Before matching | | | After matching | | |
| --- | --- | --- | --- | --- | --- | --- |
| Physical health | OR | 95%CI | P-value | OR | B | SE |
| Body mass index |  |  |  |  |  |  |
| Underweight | 1.03 | 0.53 to 2.00 | 0.928 | 0.94 | -0.06 | 0.70 |
| Normal weight | (ref.) |  |  | (ref.) | (ref.) |  |
| Overweight | 0.67 | 0.48 to 0.94 | 0.020 | 0.56 | -0.58 | 0.38 |
| Mental health | B | 95%CI | P-value |  | B | SE |
| SDQ total difficulties score | -0.07 | -0.79 to 0.66 | 0.857 |  | -1.09 | 0.62 |
| SDQ prosocial score | -0.02 | -0.29 to 0.26 | 0.900 |  | -0.03 | 0.26 |
| Depression | -0.24 | -0.84 to 0.36 | 0.432 |  | 0.01 | 0.55 |
| Self-rated health | -0.03 | -0.18 to 0.12 | 0.697 |  | 0.04 | 0.14 |

Supplementary method 1. Measurements

*Child allowance*

Child allowance status was assessed by asking parents: “How much does your household receive in child allowance per payment? Please fill in the blank with numbers”. Because we are interested in finding out whether caregivers are receiving child allowance or not, we dichotomized the responses if caregivers answered with a value more than ‘0 JPY’ (i.e. receiving) or ‘0 JPY’ (i.e. not receiving).

*Child health outcomes*

Children’s BMI was calculated with self-reported heights and weights obtained from children themselves (for 8^th^ graders) and caregivers (for 1^st^ and 5^th^ graders). BMI was expressed as z-scores representing the deviation in standard deviation units from the mean of a standard normal distribution of BMI specific to age and sex according to the WHO Child Growth Standards [1]. We categorized BMI as underweight (<1SD), normal weight (-1SD to 1SD) and overweight (+1SD).

Problem behavior was assessed by caregivers using the Japanese version of the Strengths and Difficulties Questionnaire (SDQ) [2], which was generated from the original version [3]. The SDQ scores were calculated using the scale of total difficulties scores and prosocial behavior scores. The Cronbach’s alpha for total difficulties score and prosocial behavior were 0.81 and 0.72 in the current study, respectively. Higher total difficulties scores and prosocial behavior scores indicated more problematic behavior and more prosocial behavior, respectively.

Child depressive symptoms were assessed by children (5^th^ and 8^th^ graders) using the Japanese version of the Depression Self-Rating Scale (DSRS) [4], which was translated and modified from the English version by omitting two items (i.e., “I can stick up for myself” and “I think life isn’t worth living”) to avoid psychological invasion [5]. We additionally excluded the item “I like talking with my family” from the original Japanese version to avoid biased answers, because children might respond to their questionnaire at home and might be influenced by their caregiver’s presence. In the current study, children answered a total of 15 items with the scale from “0 = never” to “2 = most of the time”, and we calculated the sum of the rated values (Cronbach’s alpha = 0.84). A higher score indicates children have more depressive symptoms.

Child’s self-rated health (SRH) was assessed among 5^th^ and 8^th^ grade children with the following question: “How do you feel about your health condition?” and responses were selected from a five-point Likert scale (1 = not good; 2 = not very good; 3 = normal; 4 = somewhat good; 5 = good). Child’s SRH was confirmed as an indicator of physical and psychological health [6].

*Household expenditure for children*

Household expenditure for children was assessed as financial investment and time investment in children [7, 8]. Caregivers were asked whether they could not afford to have the following materials in their home due to financial reasons: room or space for doing homework at home, books for children, sporting goods, stuffed animals or toys for children, and electronic game devices. Caregivers could also select an option stating that none of these materials were lacking. If at least one of them was not affordable, they were coded as “0 = not having goods for child”, otherwise they were coded as “1 = having goods for child”. They were also asked whether they participated in the following events as a family: having a birthday party, taking an annual family trip, and giving presents to each other for Christmas and New Year’s Day. If at least one event was not held due to financial difficulties, they were coded as “0 = not having family events”, otherwise they were coded as “1 = having family events”. For investment in extracurricular activities, caregivers were asked: “how much do you pay for the following activities: cram school or extracurricular educational fees, extracurricular lessons and club activities or sports clubs?” and they answered by writing the actual monthly payment. The sum of the fees was calculated and categorized as “0 JPY”, “1–10,000 JPY”, “10,001–20,000 JPY”, “20,001–30,000 JPY”, and “+ 30,001 JPY”.

Time investment was assessed by the frequency of caregivers’ interaction with children and no maltreatment of children to account for both quantity and quality of investment. Parental interaction with children was assessed with the question: “do you have any opportunity to do the following activities: helping your child with homework; exercising with your child; playing computer games with your child; talking about school life; talking about social news; talking about TV programs; cooking together; and going out together?” with a 5-point Likert scale, then the arithmetic sum of the answer was calculated (Cronbach’s alpha = 0.65) for the parent-child positive interaction score (ranged from 1 to 40) [9]. Child maltreatment was assessed by parental response to 8 items on child maltreatment, such as beating or kicking [10, 11] with a 4-point Likert scale, where “1 = often”, “2 = sometimes”, “3 = rarely”, and “4 = not at all” and all the responses were dichotomized with “1 = yes” and “0 = no” regarding the expert review based on the frequency and severity of child abuse[12]. Caregivers who reported no maltreatment were coded as “1 = no maltreatment”, otherwise “0 = maltreatment”.

*Covariates*

Respondents were asked to choose their relationship with children and categorized as follows; mother, father and others (including maternal grandmother, maternal grandfather, paternal grandmother, paternal grandfather, siblings, others, office staffs).

Parental educational attainment was categorized as follows; high school or lower (junior high school, high school dropout and high school), technical/ junior college/ university dropout, university or higher (university, graduate university) and missing (other, unknown, missing).

Parental occupation was categorized as follows; full-time employee (company executive, full employee in private company, government employee), part-time/ temporary employee, self-employed/ others (independent business, freelance professional, association staff member, other work), not working (full-time housewife, student, retirement, job seeking, other reason not to work) and missing.

Household income was asked to choose from the categories from “JPY 0”, “JPY 1-499,999”, “JPY 500,000-999,999”, “JPY 1,000,000-1,999,999”, “JPY 2,000,000-2,999,999”, “JPY 3,000,000-3,999,999”, “JPY 4,000,000-4,999,999”, “JPY 5,000,000-5,999,999”, “JPY 6,000,000-6,999,999”, “JPY 7,000,000-7,999,999”, “JPY 8,000,000-8,999,999”, “JPY 9,000,000 or more”, and “Unknown”. We converted it into “JPY < 3,000,000”, “JPY 3,000,000-5,999,999”, “JPY 6,000,000-8,999,999”, “JPY 9,000,000 or more” and “missing” considering the poverty line and average income in Japan.

Supplementary method 2. Sensitivity analysis

*Multiple imputation*

In order to take into account the missing in covariates, we analyzed by imputing the missing with multiple imputation. Percentage of missing in our variables are ranged from 0.5% to 18.5% and complete case is 53% of total cases. Missing pattern was assumed to be missing at random. We imputed missing variables using multivariate normality distribution based on the previous studies[13, 14] and relatively large sample size. All covariates including child allowance status were included in imputation model and totally 50 imputation datasets were created considering the proportion of incomplete cases[15]. Imputation was conducted with command “mi impute mvn” in STATA 15.

*Propensity score estimation and matching*

Based on the previous study, propensity score was estimated for each imputed dataset[16]. Analysis model for propensity score of child allowance and matching procedure were same as the one used main analysis. Imputed values for categorical variables (respondent relationship with children, marital status, residential area, parental educational attainment, parental occupation, parental working time, income, self-rated health, parental smoking status and child sex) were rounded to the nearest value before included into analysis model[14]. Also, as for family size, number of younger siblings, number of older siblings, number of grandparents, parental working time and K6 score, imputed values less than 0 were replaced with the smallest observed values, and values greater than the maximum values of each observed values were truncated at the maximum values.

Estimation of final coefficients was obtained by pooling parameter estimate in each imputed dataset using Rubin's Rules.

Supplementary figure 1. Histogram of propensity scores for recipients and non-recipients

Appendix 1. Child allowance system in Japan

Under the child allowance system, 15,000 JPY will be monthly given to a child younger than 3 years old; 10,000 JPY for the first- and second-born child who is older than 3 and up to 6^th^ grade; 15,000 JPY for the thirdborn and younger children aged 3 years old to graded 6 years, and 10,000 JPY for 7^th^ to 9^th^ grade children. However, any family where annual income of either parents exceeds 9.6 million JPY will lose eligibility. Because of the special benefit (*tokurei kyufu*), 5,000 JPY will be monthly given for each child in those households.

| Criteria | | Monthly allowance | Note |
| --- | --- | --- | --- |
| Age | Birth order |  |  |
| 0-3 years old | N.A. | 15,000 JPY | Income of any family members do not exceed the income threshold |
| 3 years old to 6th grade | 1^st^ / 2^nd^ | 10,000 JPY |  |
|  | 3^rd^ /later | 15,000 JPY |  |
| 7th - 9th grade | N.A. | 10,000 JPY |  |
| N.A. | N.A. | 5,000 JPY | Special benefits for family whose income exceeds the threshold |

References

1. Cole, T.J., *The LMS method for constructing normalized growth standards.* European journal of clinical nutrition, 1990. **44**(1): p. 45-60.

2. Matsuishi, T., et al., *Scale properties of the Japanese version of the Strengths and Difficulties Questionnaire (SDQ): A study of infant and school children in community samples.* Brain and Development, 2008. **30**(6): p. 410-415.

3. Goodman, R., *The Strengths and Difficulties Questionnaire: a research note.* Journal of child psychology and psychiatry, 1997. **38**(5): p. 581-586.

4. Sato Hiroshi, A.K., *The investigation of factor structure and normative data for Depression Self-Rating Scale for Children(DSRS)Japanese version [in Japanese].* Bulletin of Tsukuba Developmental and Clinical Psychology, 2002(14): p. 85-91.

5. Birleson, P., *The validity of depressive disorder in childhood and the development of a self‐rating scale: a research report.* Journal of Child Psychology and Psychiatry, 1981. **22**(1): p. 73-88.

6. Fosse, N.E. and S.A. Haas, *Validity and stability of self-reported health among adolescents in a longitudinal, nationally representative survey.* Pediatrics, 2009. **123**(3): p. e496-e501.

7. Jones, L.E., K.S. Milligan, and M. Stabile, *Child cash benefits and family expenditures: Evidence from the National Child Benefit*. 2015, National Bureau of Economic Research.

8. Milligan, K. and M. Stabile, *Do child tax benefits affect the well-being of children? Evidence from Canadian child benefit expansions.* American Economic Journal: Economic Policy, 2011. **3**(3): p. 175-205.

9. Kizuki, M., et al., *Parental time of returning home from work and child mental health among first-year primary school students in Japan: result from A-CHILD Study.* Frontiers in pediatrics, 2018. **6**: p. 179.

10. Fujiwara, T., et al., *Association of maternal developmental disorder traits with child mistreatment: a prospective study in Japan.* Child abuse & neglect, 2014. **38**(8): p. 1283-1289.

11. Tokunaga, M., et al., *Survey of child maltreatment among general population in Greater Tokyo.* Kosei no Shihyo, 2000. **47**(15): p. 3-10.

12. Isumi, A., et al., *Mediating effects of parental psychological distress and individual-level social capital on the association between child poverty and maltreatment in Japan.* Child Abuse & Neglect, 2018. **83**: p. 142-150.

13. Demirtas, H., S.A. Freels, and R.M. Yucel, *Plausibility of multivariate normality assumption when multiply imputing non-Gaussian continuous outcomes: a simulation assessment.* Journal of Statistical Computation and Simulation, 2008. **78**(1): p. 69-84.

14. Lee, K.J. and J.B. Carlin, *Multiple imputation for missing data: fully conditional specification versus multivariate normal imputation.* American journal of epidemiology, 2010. **171**(5): p. 624-632.

15. White, I.R., P. Royston, and A.M. Wood, *Multiple imputation using chained equations: issues and guidance for practice.* Statistics in medicine, 2011. **30**(4): p. 377-399.

16. Leyrat, C., et al., *Propensity score analysis with partially observed covariates: How should multiple imputation be used?* Statistical methods in medical research, 2019. **28**(1): p. 3-19.
