# Supplementary material for: Is Japan’s child allowance effective for the well-being of children? A statistical evaluation using data from K-CHILD study
Source: BMC Public Health. 2020 Oct 6;20:1503. doi: 10.1186/s12889-020-09367-0 (PMC7542372; doi:10.1186/s12889-020-09367-0)
Supplement: Supplementary file 2 — Additional file 2. Questionnaire. [file 12889_2020_9367_MOESM2_ESM.pdf]

Questionnaire

Kochi Child Health Impact of Living Difficulty (K-CHILD) study

To gardians of first-year elementary school students

- This questionnaire is for the parents of the children to whom the questionnaire was distributed
- Survey is anonymous and contains any questions on the personal information that identifies the individual. All the answers will be statistically processed. Therefore, other people will not be able to find out who wrote what.
- Please fold the completed questionnaire into three, put into a white envelope, and seal it. Together with the child's brown envelope sealed in the same way, put them in a large envelope that was enclosed in the package when it was distributed. Close it up tightly with glue or tape, then submit it to school.
- The school or homeroom teacher will not open the envelope. Only those companies commissioned by the Kochi Children's Family Division at the Kochi Prefectural Office unseal the envelope.
- There are some personal inquiries about yourself or your family. Although you do not have to answer questions that is difficult to answer or questions that you do not want to answer, please tell us your answers as they are since the privacy is protected.
- The term "child" in this questionnaire signifies the child to whom a questionnaire has been distributed. Even if the child has siblings, it refers only those children to whom a questionnaire has been distributed.

-If you have any questions, please call us. -
Division of Children and Family Affairs of the Kochi Prefectural Government's Regional Welfare Department
Phone number: 088-823-9655
(Phone reception desk is open: Monday-Friday: 8:30 - 17:15)

- ❖ Excluding December 29th to January 3rd and holidays.

Q1. Please tell us about your child's gender and date of birth.

(1) What is your gender? (circle one of them.)

1. Male	2. Female

(2) When is your birth day?

(please answer with a number in parentheses.)

Year ( ) Month ( )

Q2. What is your relationship with the child. Please answer the relationship from the child's point of view.

1. Mother	5. Father's mother	9. Staffs of institution
2. Father	6. Father's father	(→To staff of institution, please
3. Mater's mother	7. Siblings	answer the following questions as
4. Mater's father	8. Others(Be specific:)	much as you can.)

Q3. Tell us the current marital status of the child's parents.

1. Married	3. Bereaved
2. Divorced	4. Unmarried

Q4. Who are living with the child with? Please tell us each number and the total number. Please include those who are living outside of home due to business.

1. Mother	5. Father's mother	9. Younger brother (people)
2. Father	6. Father's father	10. Younger sister (people)
3. Mother's mother	7. Older brother (people)	11. Other relatives (people)
4. Mother's father	8. Older sister (people)	12. Others (people)

→ ( ) **people in total * please write the number including you and the child**

Q5. Tell us about your residence.

(1) Where are you living currently?

1. Outside of Kochi Prefecture	2. Inside of Kochi Prefecture; the name of cities ( )

(2) How many times have you moved after the child were born?

(a)	Before entering the elementary school	( ) times
(b)	After entering the elementary school	( ) times

Q6. Please tell us all the institutions your child has attended before entering elementary school.

1. Certified nursery schools<br>2. Uncertified nursery schools<br>3. Certified child care center	4. Kindergarten<br>5. There is no institution

Q7. Tell us about your future education.

Untile which level of education are you willing to let your child to get?

1. To junior high school	3. To vocational school *	5. To university or higher
2. To high school	4. To technical collage or university	6. I do not know

*This school is the one you are going to after graduating high school.

Q8. Tell us about your child's health condition.

(1) Please write down the child's height and weight as accurately as possible.

(a) Height	（　　　　　　）cm
(b) Weight	（　　　　　　）kg
(c) The time of measurement	（　　　　　　　）year（　　　　）month

(2) Which describes your child's health condition the most?

1. Good	2. Somewhat good	3. Normal	4. Not very good	5. Not good

(3) Have you ever thought your child should be seen by doctors but did not actually have your child seen within the last one year?

1. Yes	2. No

(a) To who answered "1. Yes" in Q9. What is the reason of that? Please choose the answer most closest to your situation.

1. I could not afford payments since I did not have public medical insurance.
2. I do not think I could afford the co-payments at hospital although I have joined public medical insurance.
3. Your child did not want to go to hospital.
4. The hospital is located too far to go to.
5. I was too busy to bring my child to hospital.
6. I thought I should let my child seen by doctors but on second thought I could not find any necessities.
7. My health condition was too but to bring my child to hospital.
8. Others（Be specific：　　　　　　　　　　　　　　　　　　　　　　）

(4) Tell us about your child's condition on vaccination.

* Regular vaccination (Tubeculosis (BCG), Measles, Rubella, Polio, etc.) is free of charge. Optional vaccination (Flu, Mumps, etc.) is for fees.

	Vaccinated	Not vaccinated	I do not know
(1) Regular vaccination	1	2	3
(2) Optional vaccination (Flu) * the past one year	1	2	3
(3) Optional vaccination (Mumps)	1	2	3

(a) To who answered "2. Not vaccinated" in (4) about regular vaccination.
What is the reason you do not let your child take?

1. I did not know it is for free.
2. My child did not want to be vaccinated.
3. The hospital is located too far to go to.
4. I was too busy to bring my child to hospital.
5. I thought I did not need to let my child vaccinated considering the child's condition.
6. I did not know where and when the child should be vaccinated.
7. My child had a fever at the timing of vaccination.
8. My health condition was too bad to bring child to hospital.
9. Others（Be specific：　　　　　　　　　　　　　　　　）

Q9. Tell us about your child's teeth condition.

(1) How many times does the child brush the teeth a day? (circle the number applies.)

1. More than two times a day　2. One time a day　3. Not brushing every day

(2) How long does the child usually brush the teeth a time? (circle the number applies.)

1. Less than 1 minute　2. 1 to 2 minutes　3. 3 to 5 minutes　4. More than 5 minutes

(3) Do the parents/ guardians finish brushing after child brush the teeth by them selves?

1. Yes　2. No

(4) Does your child have any cavities currently/ have your child ever had any cavities in the past?

1. Yes →（　teeth）　2. Yes（treatment is completed）→（　teeth）　3. No　4. I do not know

(a) To who answered "1. Yes" in Q10.
Does your child currently take any treatment?

1. Yes　2. No　3. I do not know

(b) To who answered “2. No” in (a).

What is the reason your child does not take any treatment although he/she has cavities.

1. Cannot pay treatment fees.
2. Since the child refused to go.
3. The dentist is located too far from home to go.
4. I am too busy to take the child to dentist.
5. Others（Be specific：　　　　　　　　　　　　　　）

Q10. Tell us about the child’s livelihood. About the school days and the behavior outside of home, please ask to children and respond together.

(1) **With whom** does your child usually spend time after school **during weekdays**?

1. Parents, siblings
2. Grandparents, relatives
3. Teachers at club activities or any other extracurricular activities
4. Other adults
5. Friends of the school
6. Friends of the different school (friend she/he met at a local sport club, neighbors, friends from the same elementary and junior high school)
7. Spending alone
8. I do not know

(2) Where does your child spend the time after school on weekdays and for how many days a week? (circle the number applies to each.)

		Every day	3 to 4 days a week	1 to 2 days a week	Never
(a)	Home	1	2	3	4
(b)	Grandparents’ or relative’s house	1	2	3	4
(c)	Friend’s house	1	2	3	4
(d)	Extracarricular activities or cram school	1	2	3	4
(e)	Extracarricular activities at school	1	2	3	4
(f)	Sport club（base ball stadium, soccer stadium, etc.）	1	2	3	4
(g)	Work places for part-time jobs	1	2	3	4
(h)	Park	1	2	3	4
(i)	Library	1	2	3	4
(j)	Restaurant, shopping street or supermarket	1	2	3	4
(k)	Game center	1	2	3	4
(l)	Others（Be specific：　　　　　　　　　）	1	2	3	4

(3) Have your child ever stayed at home after school without adults for longer than one hour?

1. Have never stayed.	2. 1 to 11 times a year	3. 1 to 3 times a month	4. More than once a week

(a) To who answered "2. 1 to 11 times a year" "3. 1 to 3 times a month" "4. Less than once a week" in (3).

How many hours do you usually stay at home per one time?

Approximately (   )hours

(4) How long does your child sleep on weekdays? (circle the number applies.)

1. More than 10 hours	3. 8 to 9 hours	5. 6 to 7 hours
2. 9 to 10 hours	4. 7 to 8 hours	6. Less than 6 hours

(5) What time does your child wake up and go to sleep? (circle the number applies to each.)

Time you wake up	Weekdays	Weekends
Earlier than 6 am	1	1
6 am to 7 am	2	2
7 am to 8 am	3	3
8 am to 9 am	4	4
9 am to 10 am	5	5
Later than 10 am	6	6

Time you go to sleep	Days except for Fri. and Sat.	Fridays and Saturdays
Ealier than 8 pm	1	1
8 pm to 9 pm	2	2
9 pm to 10 pm	3	3
10 pm to 11 pm	4	4
11 pm to 12 pm	5	5
Later than 12 pm	6	6

(6) How many hours does your child spend time for exercise that you actively move bodies for longer than 30 minutes per week?

(e.g., playing with ball, riding bicycle, soccier, baseball, karate, Kendo, gymnastic, ballet, swimming, etc.)

1. Almost no time	4. 5 to 6 days
2. 1 to 2 days	5. Almost every day
3. 3 to 4 days	

(7) How many hours does your child watch television or DVDs or movies on the internet with smartphone or tablet? Please answer considering the situation outside of home by asking children and responding together.

In addition, how many hours does your child play computer games (e.g., with television, computer, mobile gaming device, etc.) a day?

(a)Watching television or movies	
1. Do not watch	5. 3 to 4 hours
2. Less than 1 hour	6. 4 to 5 hours
3. 1 to 2 hours	7. More than 5 hours
4. 2 to 3 hours	

(b)Playing computer games	
1. Do not play	5. 3 to 4 hours
2. Less than 1 hour	6. 4 to 5 hours
3. 1 to 2 hours	7. More than 5 hours
4. 2 to 3 hours	

(8) How many books does your child read within this one month? Please answer with your child.

In addition, how many books do the mother and father read?

(a)Children	(b)Mother	(c)Father
1. Did not read	1. Did not read	1. Did not read
2. 1 book	2. 1 book	2. 1 book
3. 2 to 3 books	3. 2 to 3 books	3. 2 to 3 books
4. 4 to 7 books	4. 4 to 7 books	4. 4 to 7 books
5. 8 to 11 books	5. 8 to 11 books	5. 8 to 11 books
6. 12 books	6. 12 books	6. 12 books

* If mother and father do not cohabit with the child, please leave it as a blunk.
* You can include electronic books as well.
* Please exclude any journals or comics regardless of the contents.

Q11. Tell us about your child's diet and health.

(1) Does your child usually eat breakfast everyday on weekdays? (circle the number applies.)

1. Every day (5 days a week)	3. Few days (1 to 2 days a week)
2. Most days (3 to 4 days a week)	4. Not eat

1. To who answered "2. Most days", "3. Few days", "4. Not eat" in (1). Which of the reasons is closest to your reason why you do not eat breakfast? (circle the number applies.)

1. There is no time to eat.	4. There is no specific reason.
2. The meal is not ready.	5. Others（Be specific： ）
3. There is no appetite in the morning.	

(2) With whom does your child eat breakfast on weekdays? (circle all the numbers apply.)

1. Family members (parents)	4. Eating alone
2. Family members (siblings)	5. Do not eat breakfast
3. Other family members (grandparents, etc.)	6. Others（Be specific： ）

(3) With whom does your child eat supper on weekdays? (circle all the numbers apply.)

1. Family members (parents)	4. Eating alone
2. Family members (siblings)	5. Do not eat breakfast
3. Other family members (grandparents, etc.)	6. Others (Be specific： ）

(4) How much does your child usually eat/ drink the following foods/ drinks except for school meal?

(circle the number applies to each.)

		Evey day	4 to 5 days a week	2 to 3 days a week	Less than one day a week	Do not eat/ drink
(a)	Vegetable	1	2	3	4	5
(b)	Fruit	1	2	3	4	5
(c)	Meat or fish	1	2	3	4	5
(d)	Cup and instant noodles	1	2	3	4	5
(e)	Rice balls and bento boxes bought at convenience stores	1	2	3	4	5
(f)	Junk food	1	2	3	4	5
(g)	Snacks	1	2	3	4	5
(h)	Juices	1	2	3	4	5

(5) What does your child eat first at a meal? (circle the number applies.)

1. Rice or bread (staple foods)<br>2. Meal or fish	3. Vegetable<br>4. Soup	5. Do not decide<br>6. Do not know

(6) How often do you cook your child's meal in your family? (including easy meal like stirred eggs)

1. Almost every day<br>2. 4 to 5 days a week<br>3. 2 to 3 days a week	4. A few days a month (on weekeds/ holidays)<br>5. Rarely cook

(7) Does your child cook by yourself or help cooking at home?

1. Almost every day (more than 3 days a week)
2. Sometimes (1 to 2 days a week)
3. Rarely (1 to 3 days a month)
4. Never

(8) Does your child help house work except for cooking (tidying up, cleaning dishes, etc.)

1. Almost every day (more than 3 days a week)
2. Sometimes (1 to 2 days a week)
3. Rarely (1 to 3 days a month)
4. Never

Q12. Tell us about your child's school life and study

(1) Which statement is closest to the situation about your child and your child's school? (circle one the numbers apllies.)

	Do not agree at all.	Not agree so much.	Neigher yes or no.	Agree to some extent.	Agree so much.
(a) I like the atmosphere at class.	1	2	3	4	5
(b) I like my school teacher.	1	2	3	4	5
(c) I enjoy school life.	1	2	3	4	5
(d) I say hello to school teachers and classmates.	1	2	3	4	5
(e) I trust my teacher.	1	2	3	4	5
(f) I trust my classmates.	1	2	3	4	5
(g) I actively participate in the school events.	1	2	3	4	5
(h) I like to meet my teacher.	1	2	3	4	5
(i) I like to meet my classmates.	1	2	3	4	5

(2) Does your child understand the school lessons well? (circle one of the numbers apllies.)

1. Understand all the lessons.<br>2. Understand most of the lessons.	3. Do not understand much.<br>4. Not understand most of the lessons.	5. Not understand almost all the lessons.

(3) Whom does your child ask to help when he/she has questions about the study? (circle the number applies.)

1. Your parents	5. Teachers at cram school
2. Other family members (siblings, grandparents, etc.)	6. Other adults
3. School teachers	7. Friends of yours
4. School teachers at extracurricular activities	8. There is no one to ask

(4) How high do you think your child's grades are in your class? (circle the number applies.)

1. High	3. Middle	5. Low
2. Middle-high	4. Middle-low	6. I do not know

(5) How long does your child study except for class lessons. Please tell us your study hours a day. (circle the number applies.) * please include the time the child spends at cram school.

1. Not at all.	4. 1 hours to 2 hours.
2. Less than 30 minutes.	5. 2 hours to 3 hours.
3. 30 minutes to 1 hours.	6. More than 3 hours.

(6) Does your child go to tutoring schools for a fee, or does your child have paid tutors? If yes, how many days does your child go to or do paid tutors come to your place? (circle the number applies.)

1. No.	4. Three days a week.	7. Six days a week.

2. A day a week.<br>3. Two days a week.	5. Four days a week.<br>6. Five days a week.	8. Every day.

(7) Does your child join the after-school class * in school?

* the extracurricular class after school or during long cavation in school

1. Always participating	2. Sometimes participating	3. Rarely participating	4. Have never participated

(a) To who answered “3. Rarely participate” and “4. Have never participated” in (7).

What is the reason you do not participate so often?

① It is not held in school.<br>② I have to do house work.<br>③ I have no time due to extracurricular activities.<br>④ I am not interested in it.	⑤ There is no need to participate.<br>⑥ I did not know about it.<br>⑦ Others（Be specific： ）

(8) When did you do your summer homework this year?

(circle the number applies.)

1. Did all the homework at the beginning of summer vacation.<br>2. If anything, did it at the beginning of summer vacation.	3. Did it little by little almost every day.<br>4. If anything, did it at the end of summer vacation.<br>5. Did all the homework at the end of summer vacation.

Q13. Choose the number closest to the condition of the child during the past one month.

		Exactly true	Almost true	Somewhat true	Rarely true	Not true at all
(1)	The child could say good thing about the future.	1	2	3	4	5
(2)	The child tried to do her/his best.	1	2	3	4	5
(3)	The child could cope well with insults and mean comments.	1	2	3	4	5
(4)	The child could greet a person in a polite way.	1	2	3	4	5
(5)	The child could offer to help somebody.	1	2	3	4	5
(6)	The child could seek advice when it is necessary.	1	2	3	4	5
(7)	The child could pass up something he/she wanted, or do something he/she did not like, to get something better in the future.	1	2	3	4	5
(8)	The child could ask questions to clarify what he/she did not understand.	1	2	3	4	5

Q14. Please choose the number closest to the conditions to each of the following sections regarding your child's behavior during the past 6 months.

Please answer all the following questions even if you are unsure.

	True	Somewhat true	Certianly true
(1) Considerate of other people's feelings.	1	2	3
(2) Restless, everactive, cannot stay still for long.	1	2	3
(3) Often complains of headaches, stomachaches or sickness.	1	2	3
(4) Shares readily with other children (treats, toys, pencils etc.)	1	2	3
(5) Often has temper tantrums or hot tempers.	1	2	3
(6) Rather solitary, tends to play alone.	1	2	3
(7) Generally obedient, usually does what dults request	1	2	3
(8) Many worries, often seems worried.	1	2	3
(9) Helpful if somone is hurt, upset or feeling ill.	1	2	3
(10) Constantly fidgeting or squirming.	1	2	3
(11) Has at least one good friend.	1	2	3
(12) Often fights with other children or bullies them.	1	2	3
(13) Often unhappy, down-hearted or tearful.	1	2	3
(14) Generally liked by other children.	1	2	3
(15) Easily distracted, concentration wanders.	1	2	3
(16) Nervous or clingy in new situations, easily loses confindence.	1	2	3
(17) Kind to younger children.	1	2	3
(18) Often lies or cheats.	1	2	3
(19) Picked on or bullied by other children.	1	2	3
(20) Often volunteers to help others (parents, teachers, other children).	1	2	3
(21) Thinks things out before acting.	1	2	3
(22) Steals from home, school or elsewhere.	1	2	3
(23) Gets on better with adults than with other children.	1	2	3
(24) Many fears, easily scared.	1	2	3
(25) Sees tasks through to the end, good attention span.	1	2	3

Q15. Tell us about health condition of the child's mother and father.

*** Please write answer separately for both parents in your response form. If the parent is absent, please leave your response blank.**

(1) Tell us about the ages, heights, and weights of the child's mother and father.

* The response to this question will be used to find the correlations with physique of the child. Please respond as accurately as possible.

Mother	Father
Age ( ) years old	Age ( ) years old
Height ( ) cm	Height ( ) cm
Wight ( ) kg	Wight ( ) kg

(2) Do the child's mother and father smoke habitually. Or, Have they ever smoked?

Mother	Father
1. Currently smoking habitually.	1. Currently smoking habitually.
2. Smoked before but is not currenly.	2. Smoked before but is not currenly.
3. Have never smoked before.	3. Have never smoked before.

To who answered "1. Currently smoking habitually." And "2. Smoked before but is not currently." In (2).

(a) Do they smoke/ have they smoked inside of house?

Mother	Father
1. Yes	1. Yes
2. No	2. No

(b) Do they smoke/ have they smoked in front of the child?

Mother	Father
1. Always	1. Always
2. Sometimes	2. Sometimes
3. Never	3. Never

Q16. Tell us about the occupation and experience of the child's mother and father.

*** Please write answer separately for both parents in your response form. If the parent is absent, please leave your response blank.**

(1) What is the current occupation of the child's mother and father?

Mother	Father
1. Company executives	1. Company executives
2. Full-time employees of a private company	2. Full-time employees of a private company
3. Regular employees such as civil servants	3. Regular employees such as civil servants
4. Contract, temporary, and part-time employees	4. Contract, temporary, and part-time employees
5. Part-time employees	5. Part-time employees
6. Self-employed (including family business)	6. Self-employed (including family business)
7. Free enterprise	7. Free enterprise
8. Staff members	8. Staff members
9. Working in a way other than 1 to 8	9. Working in a way other than 1 to 8
10. Full-time housewife	10. Full-time housewife
11. Student	11. Student
12. Retired	12. Retired
13. Seeking employment	13. Seeking employment
14. Other unemployed	14. Other unemployed

(2) To who answered "1" to "9" in (1).

(a) Are you allowed to return home from work if the child got sick suddenly.

Mother		Father	
1.	Can go back immediately	1.	Can go back immediately
2.	Can go back	2.	Can go back
3.	Cannot go back easily	3.	Cannot go back easily
4.	Cannot go back ever	4.	Cannot go back ever

(b) How many hours does the child's mother and father work a week? * if they work in several workplaces, please sum up all the working time.

Mother	( ) hours a week on average
Father	( ) hours a week on average

(c) Please choose the time the child's mother and father usually arrive home. * if they work at home, please choose the time you finish to work.

Mother	Father
1. Until 6 pm	1. Until 6 pm
2. 6pm to 8pm	2. 6pm to 8pm
3. 8pm to 10pm	3. 8pm to 10pm
4. After 10pm (including early morning)	4. After 10pm (including early morning)
5. There is no fixed time	5. There is no fixed time

(3) Tell us about the last school the child's mother and father went/ is going.
(if they graduated high school at the highest, please choose "3. Graduated high school"; if they dropped out of school, please choose "2. Dropped out of high school".)

Mother	Father
1. Graduated junior high school	1. Graduated junior high school
2. dropped out of high school	2. dropped out of high school
3. Graduated high school	3. Graduated high school
4. Graduated a vocational school (after high school)	4. Graduated a vocational school (after high school)
5. Graduated a junior college/ technical college	5. Graduated a junior college/ technical college
6. Dropped out of university	6. Dropped out of university
7. Graduated university	7. Graduated university
8. Graduated graduate university	8. Graduated graduate university
9. Others（　　　　　）	9. Others（　　　　　）
10. I do not know	10. I do not know

Q17. Tell us child's home economics.

(1) How do you feel about the current living conditions?

1. Very affordable	3. Normal	5. In very financial difficulty
2. Somewhat affordable	4. In financial difficulty	

(2) How do you rate of your family's social status within a whole society?

1. High	3. Middle	5. Low
2. Middle-high	4. Middle-low	6. I do not know

(3) Among the following items, is there anything that you do not have due to financial reasons.

1. The book suited to the child's age	10.
2. The sport goods or play goods	11. A computer connected to the internet
3. The place the child can study at home	12. A bathtub for household
4. A washing machine	13. A bed per person
5. A rick cooker	14. A curtain
6. A vaccuum	15. A car
7. A hearter/ hearting appliance	16. A savings (more than 50,000 JPY)
8. An air-conditioner	17. There is nothing applicable
9. A microwave	

18.

(4) Among the following items, is there anything that you could not pay or buy due to financial reasons?

	Could not pay/buy	Could pay/buy	Not applicable (no need to pay/buy)
1. School field trips/ extracurricular activities	1	2	3
2. School textbooks	1	2	3
3. School lunch fees	1	2	3
4. Rent	1	2	3
5. Housing loans	1	2	3
6. Electricity bills	1	2	3
7. Gas bills	1	2	3
8. Water bills	1	2	3
9. Phone bills (including both lanllines and moblies)	1	2	3
10. Insurance fees for public pension, national health insurance, and/or public nursing care	1	2	3
11. Bus or train fees for commuting	1	2	3
12. Food	1	2	3
13. Clothes or bags necessary for child	1	2	3

(5) Tell us about the living costs and educational fees for the child whom the questionnaire was distributed to. How much do you spend to the following each item per month? Please answer the average cost.

* If there is no cost, please fill "0" in the parentheses.

	Monthly cost
(a) Tuition/ school fees	Approximately ( ) JPY
(b) Extracurricular educational fees such as cram school	Approximately ( ) JPY
(c) Lesson fees (excluding sport club)	Approximately ( ) JPY
(d) Sport club	Approximately ( ) JPY
(e) Allowances	Approximately ( ) JPY
(f) Mobile phone and smart phone bills	Approximately ( ) JPY

(6) Tell us about the cost you spent when the child entered elementary school (Admission fees, payment for clothes and bags, shoes and teaching materials). Did you feel it is difficult to pay?

1. Difficult	2. Somewhat difficult	3. Not so much difficult	4. Not difficult

(a) To who answered “1. Difficult” or “2. Somewhat difficult” in (6).

How did you deal with it? Please choose the closest answers.

1. Made ends meet by saving money.<br>2. Got a few second-hand items.<br>3. Borrowed money from friends and relatives.<br>4. Borrowed money from local municipalities.	5. Borrowed money from credit cards.<br>6. Could not prepare before entering.<br>7. Others（Be specific：　　　　　　）

(7) Are you receiving scholarship for high school students in your household for the sake of the child?

* Scholarship for high school students is available to household that are exempt from tax on income and aims to support educational fees except for tuition fees.

1. Receiving	2. Not receiving	3. I do not know

(a) To who answered “2. Not receiving” in (7). What is the reason you are not receiving it?

1. Not applied (necessary but not meet the requirement)
2. Not applied (necessary but not know how to apply it)
3. Not applied (necessary but feel resistence to application)
4. Not applied (feel no necessities)
5. Applied but not approved
6. Not knowing the scholarship for high school students
7. Others（Be specific：　　　　　　）

(8) How much of the following public pension and social security benefits is your household receiving at any one time?

If there is any corresponding income, please fill "0" in the parentheses.

It includes any pension/ benefits related to siblings.

Child support allowance is the allowance for household whose income is below the threshold and has only one parent.

	Payment at a time
(a) Child allowance	( ) JPY (Payed once per four months)
(b) Child support allowance	( ) JPY (Payed once per four months)
(c) Special child support allowance	( ) JPY (Payed once per four months)
(d) Annuities (survivor's pensions, age pensions)	( ) JPY (Payed once per two months)
(e) Public Allowance	( ) JPY

(9) Tell us about the income excluding public pension and social security benefits you described in (8).

How much is the approximate total annual income of all the members who share the livelihood?

* income includes income from work (including part-time jobs), business income (self-employment, etc.), agricultural income, real estate income, interest and dividends and personal pensions. Please exclude the income from public pension and social security you answered in (8).

* If there is several income resources (income from work by father, business income by mother for example), please tell us the approximate total income.

1. There is no income (0 JPY)	5. 2.00 – 2.99M JPY	9. 6.00 – 6.99M JPY
2. 1 - 49,999 JPY	6. 3.00 – 3.99M JPY	10. 7.00 – 7.99M JPY
3. 50,000 – 99,999 JPY	7. 4.00 – 4.99M JPY	11. 8.00 – 8.99M JPY
4. 1M – 1.99M JPY	8. 5.00 – 5.99M JPY	12. More than 9.00M JPY
		13. I do not know

Q18. Tell us the relationship between the child and family members including you.

(1) Do you do the following activities in your family?

	Almost every day	3-4 times a week	1-2 times a week	1-2 times a month	Rarely
a) Helping the child's study	1	2	3	4	5
b) Exercising with the child	1	2	3	4	5
c) Playing with computer games	1	2	3	4	5
d) Playing with card games/ board games	1	2	3	4	5
e) Talking about school activities with the child	1	2	3	4	5
f) Talking about social/political/economical problems with the child	1	2	3	4	5
g) Talking about TV (excluding news) with the child	1	2	3	4	5
h) Cooking with the child	1	2	3	4	5
i) Going out with the child	1	2	3	4	5

(2) Do you have opportunities to think or talk about the child's future plan (such as dreams, carrer path, and occupation) with the child in your family?

1. Often do	2. Sometimes do	3. Not so much	4. Have never done before

(3) Do you do the following activities to the child in your family?

	Yes, we do	No, we do not	
		Do not want to （Not willing to）	Cannot afford
(a) Celebrating the birth day	1	2	3
(b) Going to family trip once a year	1	2	3
(c) Giving Christmans present/ new year's gift (e.g., money)	1	2	3

(4) Do you do the following reactions to the child in your family?

	Often	Sometimes	Parely	Never
a) Hitting the child's body (e.g., bottom, hands, head, face, etc.)	1	2	3	4
b) Yelling at the child	1	2	3	4
c) Beating the child	1	2	3	4
d) Shutting the child outside	1	2	3	4
e) Neglecting the child	1	2	3	4
f) Not feeding the child	1	2	3	4
g) Insulting the child repeatedly	1	2	3	4
h) Leaving the child alone in the house at night	1	2	3	4
i) Having a big fight in front of the child	1	2	3	4

(5) **To whose child is not living together with grandparnts.**

(a) How many hours does it take to go to grandparents' house? Please tell us how to go usually and how many hours to take.

① To the mother's parents' house

1. on foot 2. By bike 3. By car 4. By bus 5. By train 6. By air plane ( ) Hours ( ) Minutes

② To the father's parents' house

1. on foot 2. By bike 3. By car 4. By bus 5. By train 6. By air plane ( ) Hours ( ) Minutes

(b) How much help of child rearing do you receive from the child's grandparents?

Parents of the mother	Parents of the father
1. On a daily basis	1. On a daily basis
2. Frequently	2. Frequently
3. Sometimes	3. Sometimes
4. Rarely	4. Rarely
5. I do not know	5. I do not know

(c) In the past one year, how often do you meet your grandparents?

	Every day/ almost every day	1-3 days a week	2 -3 days a month	Once a month	A few days a year	Never	Do not have grandparents
Mother's parents	1	2	3	4	5	6	7
Father's parents	1	2	3	4	5	6	7

Q19. Tell us about you, who are answering this questionnaire.

(1) Do you think you are happy? When we set "very happy" as 10-point and "very unhappy" as 0-point, what score do you give?

Very unhappy	Very unhappy
0---1---2---3---4----5---6---7---8---9---10	

(2) How long have you lived in the current community?

( ) years ( ) months

(3) Do you socialize with your neighbors in your family?

1. Having a very close relationship.<br>2. Having a close relationship.	3. Socializing but do not have close relationship.<br>4. Almost no/ no relationship.

(4) How often do you receive food from or share food with people in your community?

Receving food	Sharing food
1. On a daily basis	1. On a daily basis
2. Frequently	2. Frequently
3. Sometimes	3. Sometimes
4. Rarely	4. Rarely
5. Never	5. Never

(5) Are you belonging to some groups, sport club, or private organizations such as NPO, and neighborhood associations, and doing any activities?

1. Yes	2. No

(6) Have your child ever joined the following events in your community? (circle the number applies.)

1. Summer festival in neighborhood
2. Events such as a sport festival or Christmas party held by neighborhood association
3. Clearning up parks and roads, evacuating training in the community
4. Courses and classes held by child centers and community centers
5. None of 1-4 events

(7) Do you have any person you can consult with if you are in trouble or having any worries?

1. No	2. 1 to 2 people	3. 3 to 4 people	4. 5 to 7 people	5. More than 7 people

(a) To who answered any numbers except for “1. No” in (6).

Who/where is that?

1. Partners	5. Friends living nearby
2. Own parents	6. Friends living far
3. Partners’ parents	7. Staff at work places
4. Siblings/ other relatives	8. Others（Be specific：　　　　　）

(8) Have you ever consulted with the following public institutions? If not, please choose the most suitable resons.

	Yes, I have consulted.	No, I have never consulted.			
		Have never feel like to consult with.	Want to consult but felt resistant.	Time and place was not suitable.	I did not know how to consult.
1. Municipal offices (including welfare offices, health center, etc.)	1	2	3	4	5
2. Child and family support center	1	2	3	4	5
3. Teachers in schools, nursery schoos and kindergartens	1	2	3	4	5
4. School cousellors	1	2	3	4	5
5. School social workers	1	2	3	4	5
6. Demogracy and childre’s committee members	1	2	3	4	5
7. The county welfare and health department	1	2	3	4	5
8. Child guidance centers	1	2	3	4	5
9. Police	1	2	3	4	5
10. Labour information centers	1	2	3	4	5
11. Consultation sites on the internet	1	2	3	4	5
12. Other public institutions	1	2	3	4	5

(9) How do you feel about your health condition?

1. Good	2. Somewhat good	3. Normal	4. Not very good	5. Not good

(10) During the past thirty days, about how often did you feel…

	All of the time	Most of the time	Some of the time	A little fo the time	None of the time
a) … nervous?	1	2	3	4	5
b) … hopeless?	1	2	3	4	5
c) … restless or fidgety?	1	2	3	4	5
d) … so depressed that nothing could cheer you up?	1	2	3	4	5
e) … that everything was an effort?	1	2	3	4	5
f) … worthless?	1	2	3	4	5

(11) Have you ever experienced the following events during childhood?

1. My mother died.	5. My parnets played violence to me.
2. My father died.	6. I was neglected (diets, clothes).
3. My parents divorced.	7. My parents said something hurt me.
4. My father played violence to my mother.	8. We experienced financial hardships.

(12) How do you rate of your family's social status within a whole society <u>in your childhood</u>?

1. High	3. Middle	5. Low
2. Middle-high	4. Middle-low	6. I do not know

Q20. Tell us about how you collect the information and the conditions that you utilize the support.

(1) Where do you get the information on the child's education, health and medicine?

Please choose the main three sources.

1. Partner	10. SNS (facebook, Twitter, LINE, etc.)
2. My family or relatives	11. Internet or blog
3. My friends and neighbors	12. Municipal publicity
4. Teachers in school	13. News papers
5. Teachers in club activities	14. Books or magazines
6. Municipal survice office	15. Others（Be specific： ）
7. Health center	16. There is no sources.
8. Hospital	
9. Television/ radio	

(2) Have you ever received any support for the child, whom the questionnaire was distributed, from the following resources?
If not, please choose the most suitable reasons.

	I have ever used.	I have never used.					
		I did not feel like to be supported.	I want to use but did not know how to use.	Time or other system was not usable.	I do not know how to use it.	I do not know about the system.	There is no system available.
a) Community child rearing support center	1	2	3	4	5	6	7
b) Short-term childcare support center	1	2	3	4	5	6	7
c) Family support center	1	2	3	4	5	6	7
d) Children's cafeteria	1	2	3	4	5	6	7
e) Food assistance by food bank	1	2	3	4	5	6	7
f) A place where children in elementary school can spend their free time	1	2	3	4	5	6	7
g) Educational support from school	1	2	3	4	5	6	7
h) Educational support outside of school	1	2	3	4	5	6	7

＜references＞

(a) Community child rearing support center	A place where parents/ guardians of children between the ages of 0 to 3 years old can come and talk about their concerns and worries while enjoying a relaxing time together with their children.
(b) Short-term childcare support center	If you are having trouble taking care of your child at the time of hospitalization, childbirth, nursing care or work, etc., you can leave your child in a child welfare institution.
(c) Family support center	The system registers both those who want to help with childcare and those who want to help with childcare so that they can balance work and childcare and give their families more time to raise their children through support activities.
(d) Children's cafeteria	A place where private organizations provide free or discounted meals to children for the purpose of supporting their food.
(e) food bank	Activities in which NPOs and other organizations retrieve food from food manufacturers and provide it to welfare facilities and people in need of support, free of charge, for food that would otherwise be disposed of when it is still edible.

(a) Are you currently interested in using the following support system?

1. Community child rearing support center	5. Food assistance from food bank
2. Short-term childcare support center	6. A place where children in junior high school and above can spend their free time
3. Family support center	7. Educational support from school
4. Children's cafeteria	8. Educational support outside of school

(3) If there is a system that you can leave the child until the mother and father return from work after the time the child can stay in the current support system finished (approximately until 8pm)?

1. Want to use	3. I do not ned to use	5. Other（Be specific：　　　）
2. Do not want to use	4. I do not know	

(4) Have you ever used the following support system in your family? If not, please choose the most suitable rasons.

	I have used.	I have never used.				
		I have never felt like using the system (at the first place, I was not eligible to the system.)	I would like to use but did not know how to use it.	I would like to use but it was difficult to use.	I did not know how to use it.	I did not know anyting about the system.
a) Public welfare funds	1	2	3	4	5	6
b) Public Assistance	1	2	3	4	5	6
c) Mother/ father and child welfare fund	1	2	3	4	5	6
d) Child support allowance	1	2	3	4	5	6

＜References＞

a) Public welfare funds	Low-interest or interst-free loans for households with income below a certain level. You can contact with your local social welfare council.
b) Public Assisntace	If you have no other way to pay for living expenses or medical care due to illness or unemployment, etc. and have no other choice, you may be able to get a living allowance under certain conditions. This system provides assistance in eight areas: education, housing, medical care, nursing care, childbirth, occupation and funerals. Please contact your local government office for assistance.
c) Mother/ father and child welfare fund	A low-interest or interest-free loan for single-parent households. Please contact your local government office.

d) Child support allowance	An allowance to support single-parent households with incomes below a certain level.<br>Municipal offices are open to the public.

<u>This is the end of the question. Thank you for answering the questions.</u>

One last time, make sure that there are no unfilled items and put it in a brown envelope, seal it, and then along with the white envelope of your gardians, please submit it to the school in a large envelope.

Questionnaire

Kochi Child Health Impact of Living Difficulty (K-CHILD) study

To gardians of fifth-year elementary school students

- This questionnaire is for the parents of the children to whom the questionnaire was distributed
- Survey is anonymous and contains any questions on the personal information that identifies the individual. All the answers will be statistically processed. Therefore, other people will not be able to find out who wrote what.
- Please fold the completed questionnaire into three, put into a white envelope, and seal it. Together with the child's brown envelope sealed in the same way, put them in a large envelope that was enclosed in the package when it was distributed. Close it up tightly with glue or tape, then submit it to school.
- The school or homeroom teacher will not open the envelope. Only those companies commissioned by the Kochi Children's Family Division at the Kochi Prefectural Office unseal the envelope.
- There are some personal inquiries about yourself or your family. Although you do not have to answer questions that is difficult to answer or questions that you do not want to answer, please tell us your answers as they are since the privacy is protected.
- The term "child" in this questionnaire signifies the child to whom a questionnaire has been distributed. Even if the child has siblings, it refers only those children to whom a questionnaire has been distributed.

-If you have any questions, please call us. -
Division of Children and Family Affairs of the Kochi Prefectural Government's Regional Welfare Department
Phone number: 088-823-9655
(Phone reception desk is open: Monday-Friday: 8:30 - 17:15)

❖ Excluding December 29th to January 3rd and holidays.

Q1. Please tell us about your child's gender and date of birth.

(1) What is your gender? (circle one of them.)

1. Male	2. Female

(2) When is your birth day?
(please answer with a number in parentheses.)

Year ( ) Month ( )

Q2. What is your relationship with the child. Please answer the relationship from the child's point of view.

1. Mother	5. Father's mother	9. Staffs of institution
2. Father	6. Father's father	（→To staff of institution, please answer the following questions as much as you can.）
3. Mater's mother	7. Siblings	
4. Mater's father	8. Others（Be specific： ）	

Q3. Tell us the current marital status of the child's parents.

1. Married	3. Bereaved
2. Divorced	4. Unmarried

Q4. Who are living with the child with? Please tell us each number and the total number. Please include those who are living outside of home due to business.

1. Mother	5. Father's mother	9. Younger brother （ people）
2. Father	6. Father's father	10. Younger sister （ people）
3. Mother's mother	7. Older brother （ people）	11. Other relatives （ people）
4. Mother's father	8. Older sister （ people）	12. Others （ people）

→ （ ） people in total * please write the number including you and the child

Q5. Tell us about your residence.

(1) Where are you living currently?

1. Outside of Kochi Prefecture	2. Inside of Kochi Prefecture; the name of cities （ ）

(2) How many times have you moved after the child were born?

(a) Before entering the elementary school	（ ） times
(b) After entering the elementary school	（ ） times

Q6. Please tell us all the institutions your child has attended before entering elementary school.

1. Certified nursery schools	4. Kindergarten
2. Uncertified nursery schools	5. There is no institution
3. Certified child care center	

Q7. Tell us about after-school class in school *.

*** the extracurricular class after school or during long vacation in school.**

(1) If there is after-school in the school the child go to, do you want to let the child join the class?

1. Yes	2. No	3. I do not know

(2) What do you expect in after-school class?

1. Establishing basic academic skills
2. Developing study habits
3. Further developing the current academic skills
4. Overcoming challenges through detailed guidance in small groups
5. Helping studying during long vacations
6. Other（Be specific： ）
7. I do not know

(3) If there is supporting staffs coming outside of school in after-school class, what do you expect in the supporting staffs?

1. Continuing to support from the class
2. Having the ability to develop the academic skills
3. Continuing to stay for one year
4. Teaching in collaboration with teachers
5. Possessing teaching licenses
6. Others（Be specific： ）
7. I do not know

Q8. Tell us about your future education.

(1) Untile which level of education are you willing to let your child to get?

1. To junior high school	3. To vocational school *	5. To university or higher
2. To high school	4. To technical collage or university	6. I do not know

*This school is the one you are going to after graduating high school.

(2) Why is your reason of your anwer in (1)?

1. That is what my child wants to.
2. I think most people would go to that level of school.
3. I think that level is enough.
4. Considering my child’s academic ability.
5. Lack of financial resources at home.
6. Others（Be specific： ）
7. There is no specific reason.

Q9. Tell us about your child's health condition.

(1) Please write down the child's height and weight as accurately as possible.

(a) Height	( ) cm
(b) Weight	( ) kg
(c) The time of measurement	( ) year ( ) month

(2) Which describes your child's health condition the most?

1. Good	2. Somewhat good	3. Normal	4. Not very good	5. Not good

Q10. Tell us about your child's teeth condition.

Does your child have any cavities currently/ have your child ever had any cavities in the past?

1. Yes →(teeth)	2. Yes（treatment is completed） → (teeth)	3. No	4. I do not know

(a) To who answered "1. Yes" in Q10.

Does your child currently take any treatment?

1. Yes	2. No	3. I do not know

(b) To who answered "2. No" in (a).

What is the reason your child does not take any treatment although he/she has cavities.

1. Cannot pay treatment fees.
2. Since the child refused to go.
3. The dentist is located too far from home to go.
4. I am too busy to take the child to dentist.
5. Others（Be specific： ）

Q11. Have you ever thought your child should be seen by doctors but did not actually have your child seen within the last one year?

1. Yes	2. No

(a) To who answered "1. Yes" in Q9. What is the reason of that? Please choose the answer most closest to your situation.

1. I could not afford payments since I did not have public medical insurance.
2. I do not think I could afford the co-payments at hospital although I have joined public medical insurance.
3. Your child did not want to go to hospital.
4. The hospital is located too far to go to.
5. I was too busy to bring my child to hospital.
6. I thought I should let my child seen by doctors but on second thought I could not find any necessities.
7. My health condition was too but to bring my child to hospital.

8. Others（Be specific： ）

Q12. Tell us about your child's condition on vaccination.

*** Regular vaccination (Tubeculosis (BCG), Measles, Rubella, Polio, etc.) is free of charge. Optional vaccination (Flu, Mumps, etc.) is for fees.**

	Vaccinated	Not vaccinated	I do not know
(1) Regular vaccination	1	2	3
(2) Optional vaccination (Flu) * the past one year	1	2	3
(3) Optional vaccination (Mumps)	1	2	3

(a) To who answered "2. Not vaccinated" in Q10 about regular vaccination.
What is the reason you do not let your child take?

1. I did not know it is for free.
2. My child did not want to be vaccinated.
3. The hospital is located too far to go to.
4. I was too busy to bring my child to hospital.
5. I thought I did not need to let my child vaccinated considering the child's condition.
6. I did not know where and when the child should be vaccinated.
7. My child had a fever at the timing of vaccination.
8. My health condition was too bad to bring child to hospital.
9. Others（Be specific： ）

Q13. Choose the number closest to the condition of the child during the past one month.

	Exactly true	Almost true	Somewhat true	Rarely true	Not true at all
(1) The child could say good thing about the future.	1	2	3	4	5
(2) The child tried to do her/his best.	1	2	3	4	5
(3) The child could cope well with insults and mean comments.	1	2	3	4	5
(4) The child could greet a person in a polite way.	1	2	3	4	5
(5) The child could offer to help somebody.	1	2	3	4	5
(6) The child could seek advice when it is necessary.	1	2	3	4	5
(7) The child could pass up something he/she wanted, or do something he/she did not like, to get something better in the future.	1	2	3	4	5
(8) The child could ask questions to clarify what he/she did not understand.	1	2	3	4	5

Q14. Please choose the number closest to the conditions to each of the following sections regarding your child's behavior during the past 6 months.

Please answer all the following questions even if you are unsure.

	True	Somewhat true	Certianly true
(1) Considerate of other people's feelings.	1	2	3
(2) Restless, everactive, cannot stay still for long.	1	2	3
(3) Often complains of headaches, stomachaches or sickness.	1	2	3
(4) Shares readily with other children (treats, toys, pencils etc.)	1	2	3
(5) Often has temper tantrums or hot tempers.	1	2	3
(6) Rather solitary, tends to play alone.	1	2	3
(7) Generally obedient, usually does what dults request	1	2	3
(8) Many worries, often seems worried.	1	2	3
(9) Helpful if somone is hurt, upset or feeling ill.	1	2	3
(10) Constantly fidgeting or squirming.	1	2	3
(11) Has at least one good friend.	1	2	3
(12) Often fights with other children or bullies them.	1	2	3
(13) Often unhappy, down-hearted or tearful.	1	2	3
(14) Generally liked by other children.	1	2	3
(15) Easily distracted, concentration wanders.	1	2	3
(16) Nervous or clingy in new situations, easily loses confindence.	1	2	3
(17) Kind to younger children.	1	2	3
(18) Often lies or cheats.	1	2	3
(19) Picked on or bullied by other children.	1	2	3
(20) Often volunteers to help others (parents, teachers, other children).	1	2	3
(21) Thinks things out before acting.	1	2	3
(22) Steals from home, school or elsewhere.	1	2	3
(23) Gets on better with adults than with other children.	1	2	3
(24) Many fears, easily scared.	1	2	3
(25) Sees tasks through to the end, good attention span.	1	2	3

Q15. Tell us about health condition of the child's mother and father.

*** Please write answer separately for both parents in your response form. If the parent is absent, please leave your response blank.**

(1) Tell us about the ages, heights, and weights of the child's mother and father.

* The response to this question will be used to find the correlations with physique of the child. Please respond as accurately as possible.

Mother	
Age	( ) years old
Height	( ) cm
Wight	( ) kg

Father	
Age	( ) years old
Height	( ) cm
Wight	( ) kg

(2) Do the child's mother and father smoke habitually. Or, Have they ever smoked?

Mother	Father
1. Currently smoking habitually.	1. Currently smoking habitually.
2. Smoked before but is not currenly.	2. Smoked before but is not currenly.
3. Have never smoked before.	3. Have never smoked before.

To who answered "1. Currently smoking habitually." And "2. Smoked before but is not currently." In (2).

(a) Do they smoke/ have they smoked inside of house?

Mother	Father
1. Yes	1. Yes
2. No	2. No

(b) Do they smoke/ have they smoked in front of the child?

Mother	Father
1. Always	1. Always
2. Sometimes	2. Sometimes
3. Never	3. Never

Q16. Tell us about the occupation and experience of the child's mother and father.

*** Please write answer separately for both parents in your response form. If the parent is absent, please leave your response blank.**

(1) What is the current occupation of the child's mother and father?

Mother	Father
1. Company executives	1. Company executives
2. Full-time employees of a private company	2. Full-time employees of a private company
3. Regular employees such as civil servants	3. Regular employees such as civil servants
4. Contract, temporary, and part-time employees	4. Contract, temporary, and part-time employees
5. Part-time employees	5. Part-time employees
6. Self-employed (including family business)	6. Self-employed (including family business)
7. Free enterprise	7. Free enterprise
8. Staff members	8. Staff members
9. Working in a way other than 1 to 8	9. Working in a way other than 1 to 8
10. Full-time housewife	10. Full-time housewife
11. Student	11. Student
12. Retired	12. Retired
13. Seeking employment	13. Seeking employment
14. Other unemployed	14. Other unemployed

(2) To who answered "1" to "9" in (1).

(a) Are you allowed to return home from work if the child got sick suddenly.

Mother	Father
1. Can go back immediately	1. Can go back immediately
2. Can go back	2. Can go back
3. Cannot go back easily	3. Cannot go back easily
4. Cannot go back ever	4. Cannot go back ever

(b) How many hours does the child's mother and father work a week? * if they work in several workplaces, please sum up all the working time.

Mother	( ) hours a week on average
Father	( ) hours a week on average

(c) Please choose the time the child's mother and father usually arrive home. * if they work at home, please choose the time you finish to work.

Mother	Father
1. Until 6 pm	1. Until 6 pm
2. 6pm to 8pm	2. 6pm to 8pm
3. 8pm to 10pm	3. 8pm to 10pm
4. After 10pm (including early morning)	4. After 10pm (including early morning)
5. There is no fixed time	5. There is no fixed time

(3) Tell us about the last school the child's mother and father went/ is going.
(if they graduated high school at the highest, please choose "3. Graduated high school"; if they dropped out of school, please choose "2. Dropped out of high school".)

Mother	Father
1. Graduated junior high school	1. Graduated junior high school
2. dropped out of high school	2. dropped out of high school
3. Graduated high school	3. Graduated high school
4. Graduated a vocational school (after high school)	4. Graduated a vocational school (after high school)
5. Graduated a junior college/ technical college	5. Graduated a junior college/ technical college
6. Dropped out of university	6. Dropped out of university
7. Graduated university	7. Graduated university
8. Graduated graduate university	8. Graduated graduate university
9. Others（　　　　　）	9. Others（　　　　　）
10. I do not know	10. I do not know

Q17. Tell us child's home economics.

(1) How do you feel about the current living conditions?

1. Very affordable	3. Normal	5. In very financial difficulty
2. Somewhat affordable	4. In financial difficulty	

(2) How do you rate of your family's social status within a whole society?

1. High	3. Middle	5. Low
2. Middle-high	4. Middle-low	6. I do not know

(3) Among the following items, is there anything that you do not have due to financial reasons.

1. A washing machine	8. A computer connected to the internet
2. A rick cooker	9. A bathtub for household
3. A vaccuum	10. A bed per person
4. A hearter/ hearting appliance	11. A curtain
5. An air-conditioner	12. A car
6. A microwave	13. A savings (more than 50,000 JPY)
7. A phone (including both landlines and mobiles)	14. There is nothing applicable

(4) Among the following items, is there anything that you could not pay or buy due to financial reasons?

	Could not pay/buy	Could pay/buy	Not applicable (no need to pay/buy)
1. School field trips/ extracurricular activities	1	2	3
2. School textbooks	1	2	3
3. School lunch fees	1	2	3
4. Rent	1	2	3
5. Housing loans	1	2	3
6. Electricity bills	1	2	3
7. Gas bills	1	2	3
8. Water bills	1	2	3
9. Phone bills (including both lanllines and moblies)	1	2	3
10. Insurance fees for public pension, national health insurance, and/or public nursing care	1	2	3
11. Bus or train fees for commuting	1	2	3
12. Food	1	2	3
13. Clothes or bags necessary for child	1	2	3

(5) Tell us about the living costs and educational fees for the child whom the questionnaire was distributed to. How much do you spend to the following each item per month? Please answer the average cost.

* If there is no cost, please fill "0" in the parentheses.

	Monthly cost
(a) Tuition/ school fees	Approximately ( ) JPY
(b) Extracurricular educational fees such as cram school	Approximately ( ) JPY
(c) Lesson fees (excluding sport club)	Approximately ( ) JPY
(d) Sport club	Approximately ( ) JPY
(e) Allowances	Approximately ( ) JPY
(f) Mobile phone and smart phone bills	Approximately ( ) JPY

(6) Are you receiving scholarship for high school students in your household for the sake of the child?

* Scholarship for high school students is available to household that are exempt from tax on income and aims to support educational fees except for tuition fees.

1. Receiving	2. Not receiving	3. I do not know

(a) To who answered “2. Not receiving” in (7). What is the reason you are not receiving it?

1. Not applied (necessary but not meet the requirement)
2. Not applied (necessary but not know how to apply it)
3. Not applied (necessary but feel resistence to application)
4. Not applied (feel no necessities)
5. Applied but not approved
6. Not knowing the scholarship for high school students
7. Others（Be specific：　　　　　　　　　　　　　　　　　　　　　　）

(7) How much of the following public pension and social security benefits is your household receiving at any one time?

If there is any corresponding income, please fill “0” in the parentheses.

It includes any pension/ benefits related to siblings.

Child support allowance is the allowance for household whose income is below the threshold and has only one parent.

	Payment at a time
(a) Child allowance	(　　　　　) JPY （Payed once per four months）
(b) Child support allowance	(　　　　　) JPY （Payed once per four months）
(c) Special child support allowance	(　　　　　) JPY （Payed once per four months）
(d) Annuities (survivor’s pensions, age pensions)	(　　　　　) JPY （Payed once per two months）
(e) Public Allowance	(　　　　　) JPY

(8) Tell us about the income excluding public pension and social security benefits you described in (7).

How much is the approximate total annual income of all the members who share the livelihood?

* income includes income from work (including part-time jobs), business income (self-employment, etc.), agricultural income, real estate income, interest and dividends and personal pensions. Please exclude the income from public pension and social security you answered in (7).

* If there is several income resources (income from work by father, business income by mother for example), please tell us the approximate total income.

1. There is no income (0 JPY)	5. 2.00 – 2.99M JPY	9. 6.00 – 6.99M JPY
2. 1 - 49,999 JPY	6. 3.00 – 3.99M JPY	10. 7.00 – 7.99M JPY
3. 50,000 – 99,999 JPY	7. 4.00 – 4.99M JPY	11. 8.00 – 8.99M JPY
4. 1M – 1.99M JPY	8. 5.00 – 5.99M JPY	12. More than 9.00M JPY
		13. I do not know

Q18. Tell us the relationship between the child and family members including you.

(1) Do you do the following activities in your family?

	Almost every day	3-4 times a week	1-2 times a week	1-2 times a month	Rarely
a) Helping the child's study	1	2	3	4	5
b) Exercising with the child	1	2	3	4	5
c) Talking about school activities with the child	1	2	3	4	5
d) Talking about social/political/economical problems with the child	1	2	3	4	5
e) Talking about TV (excluding news) with the child	1	2	3	4	5
f) Cooking with the child	1	2	3	4	5
g) Going out with the child	1	2	3	4	5

(2) Do you have opportunities to think or talk about the child's future plan (such as dreams, carrer path, and occupation) with the child in your family?

1. Often do	2. Sometimes do	3. Not so much	4. Have never done before

(3) Do you have high expectations for your child's potential in your family?

1. Have high expectations	3. Have few expectations	5. I do not know
2. Have a few expectations	4. Have no expectations	

(4) How often do you cook your child's meal in your family? (including easy meal like stirred eggs)

1. Almost every day	4. A few days a month (on weekeds/ holidays)
2. 4 to 5 days a week	5. Rarely cook
3. 2 to 3 days a week	

(5) Do you do the following activities to the child in your family?

	Yes, we do	No, we do not	
		Do not want to（Not willing to）	Cannot afford
(a) Celebrating the birth day	1	2	3
(b) Going to family trip once a year	1	2	3
(c) Giving Christmans present/ new year's gift (e.g., money)	1	2	3

(6) Do you do the following reactions to the child in your family?

	Often	Sometimes	Parely	Never
a) Hitting the child's body (e.g., bottom, hands, head, face, etc.)	1	2	3	4
b) Yelling at the child	1	2	3	4
c) Beating the child	1	2	3	4
d) Shutting the child outside	1	2	3	4
e) Neglecting the child	1	2	3	4
f) Not feeding the child	1	2	3	4
g) Insulting the child repeatedly	1	2	3	4
h) Leaving the child alone in the house at night	1	2	3	4
i) Having a big fight in front of the child	1	2	3	4

(7) **<u>To whose child is not living together with grandparnts.</u>**

(a) How many hours does it take to go to grandparents' house? Please tell us how to go usually and how many hours to take.

① To the mother's parents' house

1. on foot 2. By bike 3. By car 4. By bus 5. By train 6. By air plane （ ）Hours（ ）Minutes

② To the father's parents' house

1. on foot 2. By bike 3. By car 4. By bus 5. By train 6. By air plane （ ）Hours（ ）Minutes

(b) How much help of child rearing do you receive from the child's grandparents?

Parents of the mother	Parents of the father
1. On a daily basis	1. On a daily basis
2. Frequently	2. Frequently
3. Sometimes	3. Sometimes
4. Rarely	4. Rarely
5. I do not know	5. I do not know

Q19. Tell us about you, who are answering this questionnaire.

(1) Do you think you are happy? When we set "very happy" as 10-point and "very unhappy" as 0-point, what score do you give?

Very unhappy	Very unhappy
0---1---2---3---4----5---6---7---8---9---10	

(2) How long have you lived in the current community?

( ) years ( ) months

(3) Do you socialize with your neighbors in your family?

1. Having a very close relationship.	3. Socializing but do not have close relationship.
2. Having a close relationship.	4. Almost no/ no relationship.

(4) How often do you receive food from or share food with people in your community?

Receving food	Sharing food
1. On a daily basis	1. On a daily basis
2. Frequently	2. Frequently
3. Sometimes	3. Sometimes
4. Rarely	4. Rarely
5. Never	5. Never

(5) Are you belonging to some groups, sport club, or private organizations such as NPO, and neighborhood associations, and doing any activities?

1. Yes	2. No

(6) Do you have any person you can consult with if you are in trouble or having any worries?

1. No	2. 1 to 2 people	3. 3 to 4 people	4. 5 to 7 people	5. More than 7 people

(a) To who answered any numbers except for “1. No” in (6).

Who/where is that?

1. Partners	5. Friends living nearby
2. Own parents	6. Friends living far
3. Partners’ parents	7. Staff at work places
4. Siblings/ other relatives	8. Others（Be specific：　　　　　　）

(7) Have you ever consulted with the following public institutions? If not, please choose the most suitable resons.

	Yes, I have consulted.	No, I have never consulted.			
		Have never feel like to consult with.	Want to consult but felt resistant.	Time and place was not suitable.	I did not know how to consult.
1. Municipal offices (including welfare offices, health center, etc.)	1	2	3	4	5
2. Child and family support center	1	2	3	4	5
3. Teachers in schools, nursery schoos and kindergartens	1	2	3	4	5
4. School cousellors	1	2	3	4	5
5. School social workers	1	2	3	4	5
6. Demogracy and childre’s committee members	1	2	3	4	5
7. The county welfare and health department	1	2	3	4	5
8. Child guidance centers	1	2	3	4	5
9. Police	1	2	3	4	5
10. Labour information centers	1	2	3	4	5
11. Consultation sites on the internet	1	2	3	4	5
12. Other public institutions	1	2	3	4	5

(8) How do you feel about your health condition?

1. Good	2. Somewhat good	3. Normal	4. Not very good	5. Not good

(9) During the past thirty days, about how often did you feel…

	All of the time	Most of the time	Some of the time	A little fo the time	None of the time
a) … nervous?	1	2	3	4	5
b) … hopeless?	1	2	3	4	5
c) … restless or fidgety?	1	2	3	4	5
d) … so depressed that nothing could cheer you up?	1	2	3	4	5
e) … that everything was an effort?	1	2	3	4	5
f) … worthless?	1	2	3	4	5

(10) Have you ever experienced the following events during childhood?

① My mother died.	⑤ My parnets played violence to me.
② My father died.	⑥ I was neglected (diets, clothes).
③ My parents divorced.	⑦ My parents said something hurt me.
④ My father played violence to my mother.	⑧ We experienced financial hardships.

(11) How do you rate of your family's social status within a whole society <u>in your childhood</u>?

1. High	3. Middle	5. Low
2. Middle-high	4. Middle-low	6. I do not know

Q20. Tell us about how you collect the information and the conditions that you utilize the support.

(1) Where do you get the information on the child's education, health and medicine?

Please choose the main three sources.

1. Partner	10. SNS (facebook, Twitter, LINE, etc.)
2. My family or relatives	11. Internet or blog
3. My friends and neighbors	12. Municipal publicity
4. Teachers in school	13. News papers
5. Teachers in club activities	14. Books or magazines
6. Municipal survice office	15. Others（Be specific： ）
7. Health center	16. There is no sources.
8. Hospital	
9. Television/ radio	

(2) Have you ever received any support for the child, whom the questionnaire was distributed, from the following resources?

If not, please choose the most suitable reasons.

	I have ever used.	I have never used.					
		I did not feel like to be supported.	I want to use but did not know how to use.	Time or other system was not usable.	I do not know how to use it.	I do not know about the system.	There is no system available.
a) Community child rearing support center	1	2	3	4	5	6	7
b) Short-term childcare support center	1	2	3	4	5	6	7
c) Family support center	1	2	3	4	5	6	7
d) Children's cafeteria	1	2	3	4	5	6	7
e) Food assistance by food bank	1	2	3	4	5	6	7
f) A place where children in elementary school can spend their free time	1	2	3	4	5	6	7
g) Educational support from school	1	2	3	4	5	6	7
h) Educational support outside of school	1	2	3	4	5	6	7

＜references＞

(a) Community child rearing support center	A place where parents/ guardians of children between the ages of 0 to 3 years old can come and talk about their concerns and worries while enjoying a relaxing time together with their children.
(b) Short-term childcare support center	If you are having trouble taking care of your child at the time of hospitalization, childbirth, nursing care or work, etc., you can leave your child in a child welfare institution.
(c) Family support center	The system registers both those who want to help with childcare and those who want to help with childcare so that they can balance work and childcare and give their families more time to raise their children through support activities.
(d) Children's cafeteria	A place where private organizations provide free or discounted meals to children for the purpose of supporting their food.
(e) food bank	Activities in which NPOs and other organizations retrieve food from food manufacturers and provide it to welfare facilities and people in need of support, free of charge, for food that would otherwise be disposed of when it is still edible.

(a) Are you currently interested in using the following support system?

1. Community child rearing support center	5. Food assistance from food bank
2. Short-term childcare support center	6. A place where children in junior high school and above can spend their free time
3. Family support center	7. Educational support from school
4. Children's cafeteria	8. Educational support outside of school

(3) If there is a system that you can leave the child until the mother and father return from work after the time the child can stay in the current support system finished (approximately until 8pm)?

1. Want to use	3. I do not ned to use	5. Other（Be specific：　　　　）
2. Do not want to use	4. I do not know	

(4) Have you ever used the following support system in your family? If not, please choose the most suitable rasons.

	I have used.	I have never used.				
		I have never felt like using the system (at the first place, I was not eligible to the system.)	I would like to use but did not know how to use it.	I would like to use but it was difficult to use.	I did not know how to use it.	I did not know anyting about the system.
a) Public welfare funds	1	2	3	4	5	6
b) Public Assistance	1	2	3	4	5	6
c) Mother/ father and child welfare fund	1	2	3	4	5	6
d) Child support allowance	1	2	3	4	5	6

＜References＞

a) Public welfare funds	Low-interest or interst-free loans for households with income below a certain level. You can contact with your local social welfare council.
b) Public Assisntace	If you have no other way to pay for living expenses or medical care due to illness or unemployment, etc. and have no other choice, you may be able to get a living allowance under certain conditions. This system provides assistance in eight areas: education, housing, medical care, nursing care, childbirth, occupation and funerals. Please contact your local government office for assistance.
c) Mother/ father and child welfare fund	A low-interest or interest-free loan for single-parent households. Please contact your local government office.

d) Child support allowance	An allowance to support single-parent households with incomes below a certain level.<br>Municipal offices are open to the public.

<u>This is the end of the question. Thank you for answering the questions.</u>

One last time, make sure that there are no unfilled items and put it in a brown envelope, seal it, and then along with the white envelope of your gardians, please submit it to the school in a large envelope.

Questionnaire

Kochi Child Health Impact of Living Difficulty (K-CHILD) study

To fifth-year elementary school students

Purpose of the survey

- This is the questionnaire aiming to find out the dreams, worries and living situations of the children in Kochi Prefecture.

A request to you

- Please complete this servey on your own. There is no need for your family to see your answer.
- Please do not write down your name.
- You do not need to questions that you would not like to answer. Also, there are no wrong or correct answers. Please write your thoughts on this question.
- When you are asked about "parents" in the question, please respond as a parent or a guardian of you.

How to submit your questionnaire

- When you have finished answering, fold it into three and put it in a brown envelop by yourself. Then close it with glue or tape.
- Put your envelope and your guardian's envelope in a large envelope together.
- Close the large envelope tightly with glue or tape, and take it to school.

-If you have any questions, please call us. -
Division of Children and Family Affairs of the Kochi Prefectural Government's Regional Welfare Department
Phone number: 088-823-9655
(Phone reception desk is open: Monday-Friday: 8:30 - 17:15)

- ❖ Excluding December 29th to January 3rd and holidays.

Q1. Do you have any of the following items? Please choose the one that is closest to your situation. (please circle one of the numbers that apply to each.)

	I have.	I do not have	
		I want to have but do not.	I do not want.
(1) My own book (except for textbooks)	1	2	3
(2) My own room (including shared room with your siblings)	1	2	3
(3) A computer connected to the internet at home	1	2	3
(4) The place where you can study at home	1	2	3
(5) My own study desk	1	2	3
(6) Sport equipments (the glove for baseball, soccer ball, etc.)	1	2	3
(7) Electronic gaming device	1	2	3
(8) Bicycle	1	2	3
(9) Allowance to buy some snacks and goods	1	2	3
(10) A cell phone, a smart phone	1	2	3
(11) Portable music player etc.	1	2	3

Q2. Do you have an occupation that you want to be in the future?

1. Yes
2. No

(a) <u>To who answered "no" in Q4.</u>

What is the reason you do not have any occupation that you want to be?

1. I am satisfied in the current situation alrealy.
2. I do not think I am able to be what I want to be.
3. I cannot think of anything specific.
4. I do not know.

Q3. Please tell us about your friends and family

(1) How many friends like the following do you have in total? If you do not have, please answer "0".

(please answer with a number in parentheses.)

a) A good friend in a same school ( ) people

b) A good friend in a different school ( ) people

c) A friend you can consult with if you have any worries ( ) people

(2) How do you think about the relationship with the friends of yours?

		Strongly agree	Agree	Not agree	Disagree
a)	I think I am playing with my friends so much.	1	2	3	4
b)	I think I am getting along with my friends.	1	2	3	4
c)	I think my friends like me.	1	2	3	4
d)	I think I am different from other friends.	1	2	3	4

(3) How much influence do you think does your opinions and behaviors have on your classmates?

1. Very much	2. To some extent	3. Not very much	4. Not at all

(4) Please tell us about your relationship with your parents. Please choose every answer apply.

1. Talking about school events and my friends.
2. Talking about my future and carrer.
3. Talking about social events and news.
4. Helping me learn.
5. Listening to me.

(5) In the past one year, how often do you meet your grandparents?

	Every day/ almost every day	1-3 days a week	2 -3 days a month	Once a month	A few days a year	Never	Do not have grandparents
Mother's parents	1	2	3	4	5	6	7
Father's parents	1	2	3	4	5	6	7

Q4. Please tell us about your daily life.

(1) **With whom** do you usually spend time after school **during weekdays.** (circle one of the numbers applies.)

1. Your family (including grandparents and relatives)
2. Adults except for your family (neighbors, teachers at a cram school, coaches at a sport club, etc.)
3. Friends of my school
4. Fiends of the different school (friends you met at a local sport club, neighbors, friends from the same elementary and junior high school)
5. Coworkers such as part-time jobs
6. Spending alone

(2) Where do you spend your time after school on weekdays and for how many days a week? (circle the number applies to each.)

		Every day	3 to 4 days a week	1 to 2 days a week	Never
(a)	Home	1	2	3	4
(b)	Grandparents' or relative's house	1	2	3	4
(c)	Friend's house	1	2	3	4
(d)	Extracarricular activities or cram school	1	2	3	4
(e)	Extracarricular activities at school	1	2	3	4
(f)	Sport club（base ball stadium, soccer stadium, etc.）	1	2	3	4
(g)	Work places for part-time jobs	1	2	3	4
(h)	Park	1	2	3	4
(i)	Library	1	2	3	4
(j)	Restaurant, shopping street or supermarket	1	2	3	4
(k)	Game center	1	2	3	4
(l)	Others（Be specific：　　　　　　　）	1	2	3	4

(3) Which place are you relaxed the most from (a) to (i) in (2)? Please choose the one and write down the alphabet in the parenthesis. If you do not have place you are relaxed, please circle "no, there is no place I feel relaxed."

Alphabet（　　　　　）	No, there is no place I feel relaxed.

(4) Have you ever stayed at home after school without adults for longer than one hour?

1. I have never stayed.	2. 1 to 11 times a year	3. 1 to 3 times a month	4. Less than once a week

(a) To who answered "2. 1 to 11 times a year" "3. 1 to 3 times a month" "4. Less than once a week" in (4).

How many hours do you usually stay at home per one time?

Approximately (　　　　)hours

(5) How long do you spend time for each activity? (circle the number applies to each.)

		More than 2 hours a day	1 to 2 hours a day	Less than 1 hour a day	4 to 5 days a week	2 to 3 days a week	One day a week	Merely/ never
(a)	Playing with a computer game	1	2	3	4	5	6	7
(b)	Watching TV or DVDs	1	2	3	4	5	6	7
(c)	Using a cell phone, tablet or smart phone	1	2	3	4	5	6	7
(d)	Reading a book	1	2	3	4	5	6	7
(e)	Indoor activity except for (a) to (d)	1	2	3	4	5	6	7
(f)	Houseworks (laundry, clearning, cooking, tyding up, etc.) or supporting your siblings.	1	2	3	4	5	6	7
(g)	Exercise or physical activities	1	2	3	4	5	6	7

(6) How many books did you read within the last one month? (circle the number applies.)

※Please count the books you read outside of home. Also, count the electronic books that you can read with a laptop, tablet or smartphone. Please exclude any journals or comics regardless of their contents.

1. Did not read	3. 2 to 3 books	5. 8 to 11 books
2. 1 book	4. 4 to 7 books	6. More than 11 books

(7) How long do you sleep on weekdays? (circle the number applies.)

1. More than 10 hours	3. 8 to 9 hours	5. 6 to 7 hours
2. 9 to 10 hours	4. 7 to 8 hours	6. Less than 6 hours

(8) What time do you wake up and go to sleep? (circle the number applies to each.)

Time you wake up	Weekdays	Weekends
Earlier than 6 am	1	1
6 am to 7 am	2	2
7 am to 8 am	3	3
8 am to 9 am	4	4
9 am to 10 am	5	5
Later than 10 am	6	6

Time you go to sleep	Days except for Fri. and Sat.	Fridays and Saturdays
Ealier than 8 pm	1	1
8 pm to 9 pm	2	2
9 pm to 10 pm	3	3
10 pm to 11 pm	4	4
11 pm to 12 pm	5	5
Later than 12 pm	6	6

Q8. Please tell us about your diet and health.

(1) Do you usually eat breakfast everyday on weekdays? (circle the number applies.)

1. Every day (5 days a week)	3. Few days (1 to 2 days a week)
2. Most days (3 to 4 days a week)	4. Not eat

(a) To who answered "2. Most days", "3. Few days", "4. Not eat" in (1). Which of the reasons is closest to your reason why you do not eat breakfast? (circle the number applies.)

1. There is no time to eat.	4. There is no specific reason.
2. The meal is not ready.	5. Others（Be specific： ）
3. There is no appetite in the morning.	

(2) With whom do you eat breakfast on weekdays? (circle all the numbers apply.)

1. Family members (parents)	4. Eating alone
2. Family members (siblings)	5. Do not eat breakfast
3. Other family members (grandparents, etc.)	6. Others（Be specific： ）

(3) With whom do you eat supper on weekdays? (circle all the numbers apply.)

1. Family members (parents)	4. Eating alone
2. Family members (siblings)	5. Do not eat breakfast
3. Other family members (grandparents, etc.)	6. Others (Be specific： ）

(4) How much do you usually eat/ drink the following foods/ drinks except for school meal?

(circle the number applies to each.)

		Evey day	4 to 5 days a week	2 to 3 days a week	Less than one day a week	Do not eat/ drink
(a)	Vegetable	1	2	3	4	5
(b)	Fruit	1	2	3	4	5
(c)	Meat or fish	1	2	3	4	5
(d)	Cup and instant noodles	1	2	3	4	5
(e)	Rice balls and bento boxes bought at convenience stores	1	2	3	4	5
(f)	Junk food	1	2	3	4	5
(g)	Snacks	1	2	3	4	5
(h)	Juices	1	2	3	4	5

(5) What do you eat first at a meal? (circle the number applies.)

1. Rice or bread (staple foods)	3. Vegetable	5. Do not decide
2. Meal or fish	4. Soup	6. Do not know

(6) Are you currently on a diet? (circle the number applies.)

1. Yes. 2. Not currently but was on a diet in the past.	3. No.

↓

(6) To who answered “1. Yes”, “2. Not currently but was on a diet in the past” in (6). What are you doing/ have you done to reduce your weight? (circle all the numbers apply.)

1. Reducing the amount of meal.	6. Calculating calory intakes.
2. Cutting back on snacks and evening meals.	7. Doing exercise.
3. Skipping a meal.	8. Taking a medicine.
4. Eating only certain foods (e.g., vegetables, tofu).	9. Spitting out what you eat.
5. Not eating certain foods (e.g., carbohydrate such as rice or bread).	10. Others（Be specific：　　　　　　）

(7) Do you cook by yourself or help cooking at home? (circle the number applies.)

1. Almost every day (more than 3 days a week)	2. Often (1 to 2 days a week)
3. Sometimes (1 to 3 days a month)	4. Never

(8) How do you feel about your health condition? (circle the number applies.)

1. Good	2. Somewhat good	3. Normal	4. Not very good	5. Not good

(9) How many times do you brush your teeth a day? (circle the number applies.)

1. More than two times a day	2. One time a day	3. Not brushing every day

(10) How long do you usually brush your teeth a time? (circle the number applies.)

1. Less than 1 minute	2. 1 to 2 minutes	3. 3 to 5 minutes	4. More than 5 minutes

(11) Approximately how many cavities do you have right now? Please include the cavities currently under treatment.

* please write down “0” if you do not have any cavity. Please do no include the cavities complete the treatment.

（　　　　）teeth

(a) To who answered any number except for “0” in (11) (who have any cavities currenty).

Are you currently under treatment of cavities? (circle the number applies.)

1. Yes	2. No	3. I do not know

(b) To who answered “2. No” in (a).

What is the reason? (circle the number applies.)

1. Because treatment at dentist is painful.
2. Because treatment at dentist is scary.
3. Because there is no time to go to dentist.
4. Because there is no dentist nearby.
5. Because cavities do not hurt.
6. Because I have never been to dentist.
7. Because parents do not bring me to dentist.
8. Other reasons（Be specific：　　　　　　　　　　　　　　　　　　）

Q9. Please tell us about your school and study.

(1) Which statement is closest to the situation about you and your school? (circle one the numbers apllies.)

	Do not agree at all.	Not agree so much.	Neigher yes or no.	Agree to some extent.	Agree so much.
(a) I like the atmosphere at class.	1	2	3	4	5
(b) I like my school teacher.	1	2	3	4	5
(c) I enjoy school life.	1	2	3	4	5
(d) I say hello to school teachers and classmates.	1	2	3	4	5
(e) I trust my teacher.	1	2	3	4	5
(f) I trust my classmates.	1	2	3	4	5
(g) I actively participate in the school events.	1	2	3	4	5
(h) I like to meet my teacher.	1	2	3	4	5
(i) I like to meet my classmates.	1	2	3	4	5

(2) Do you understand your school lessons well? (circle one of the numbers apllies.)

1. Understand all the lessons.<br>2. Understand most of the lessons.	3. Do not understand much.<br>4. Not understand most of the lessons.	5. Not understand almost all the lessons.

(3) Do you understand your school lessons well? (circle one of the numbers apllies.)

1. Understand all the lessons.<br>2. Understand most of the lessons.	3. Do not understand much.<br>4. Not understand most of the lessons.	5. Not understand almost all the lessons.

(4) Whom do you ask to help when you have questions about your study? (circle the number applies.)

1. Your parents<br>2. Other family members (siblings, grandparents, etc.)<br>3. School teachers<br>4. School teachers at extracurricular activities	5. Teachers at cram school<br>6. Other adults<br>7. Friends of yours<br>8. There is no one to ask

(5) How high do you think your grades are in your class? (circle the number applies.)

1. High<br>2. Middle-high	3. Middle<br>4. Middle-low	5. Low<br>6. I do not know

(6) What subject are you good at? (circle all the numbers apply.)

1. Algebra<br>2. Language<br>3. Science	4. Society<br>5. P.E.<br>6. Art	7. Music<br>8. Home economics<br>9. None is applicable

(8) How long do you study except for class lessons. Please tell us your study hours a day. (circle the number applies.) * please include the time you spend at cram school.

1. Not at all.<br>2. Less than 30 minutes.<br>3. 30 minutes to 1 hours.	4. 1 hours to 2 hours.<br>5. 2 hours to 3 hours.<br>6. More than 3 hours.

(9) Do you go to tutoring schools for a fee, or do you have paid tutors? If yes, how many days do you go to or do paid tutors come to your place? (circle the number applies.)

1. No.<br>2. A day a week.<br>3. Two days a week.	4. Three days a week.<br>5. Four days a week.<br>6. Five days a week.	7. Six days a week.<br>8. Every day.

(8) Are you participating the after-school class in school*? Choose the number closest to your condition.

* The educational support conducted after school or during long vacation in school.

1. Participating always	2. Sometimes participating	3. Rarely participate	4. Have never participated

(a) To who answered "3. Rarely participate" and "4. Have never participated" in (8).

What is the reason you do not participate so often?

1. It is not held in school.<br>2. I have to do house work.<br>3. I have no time due to extracurricular activities.<br>4. I am not interested in it.	5. There is no need to participate.<br>6. I did not know about it.<br>7. Others（Be specific：　　　　　　　　　）

(9) If you participate in after-school class in school, what do you want to do?

1. I want to ask what I could not understand at class.<br>2. I want to ask where I could not understand about homework (including homework during summer vacation).<br>3. I want to listen to what I am interested in or something difficult.	4. Others（Be specific：　　　　　　　　　　　　）<br>5. I do not know.

(10) When did you do your summer homework this year?

(circle the number applies.)

1. Did all the homework at the beginning of summer vacation.<br>2. If anything, did it at the beginning of summer vacation.	3. Did it little by little almost every day.<br>4. If anything, did it at the end of summer vacation.<br>5. Did all the homework at the end of summer vacation.

Q7. Please tell us about your communy.

(1) Have you ever joined the following events in your community? (circle the number applies.)

1. Summer festival in neighborhood
2. Events such as a sport festival or Christmas party held by neighborhood association
3. Clearning up parks and roads, evacuating training in the community
4. Courses and classes held by child centers and community centers
5. None of 1-4 events

(2) Are there any adults around you, other than your parents, who are like the following? (circle the numbers applies.)

1. Whom you can trust<br>2. Whom you can consult with no difficulties<br>3. Whom you look up<br>4. Whom you would like to be like.	5. Who take care of you<br>6. Who say hello to you when you meet<br>7. There is no one like 1-6.

Q8. Tell us about yourself.

(1) Do you think you are happy? When we set "very happy" as 10-point and "very unhappy" as 0-point, what score do you give? (circle the number applies.)

Very unhappy	Very unhappy
0---1---2---3---4----5---6---7---8---9---10	

(2) Please choose one number which is closet to your usual condition.
(circle the number applies to each.)

	No	Somewhat no	Somewhat yes	Yes
(a) Do you have confidence in yourself?	1	2	3	4
(b) Do you think you can do most things better than others?	1	2	3	4
(c) Do you think you have a lot to be proud of?	1	2	3	4
(d) Do you feel nothing you do seems to work?	1	2	3	4
(e) Are you satisfied with yourself?	1	2	3	4
(f) Do you think you will become a great person?	1	2	3	4
(g) Do you think you are not a very useful person?	1	2	3	4
(h) Are you confident in your opinion?	1	2	3	4
(i) Do you think you do not have much to offer yourself?	1	2	3	4

(3) Have you ever experienced the following situations? (circle the number applies.)

		A lot	Sometimes	Rarely	Never	I do not know
(a)	I did not want to go to school.	1	2	3	4	5
(b)	I was absent from school (due not to illness).	1	2	3	4	5
(c)	I was bullied.	1	2	3	4	5

(4) We do not have happy days all the time; there are days when we feel a little longely and days when we are not happy. How you have felt in the past week? There is no good or bad answer. Please answer as honestly as you can. (circle one of the numbers applies to each.)

		Mostly	Sometimes	Never
(a)	I look forward to thing as much as I used to.	1	2	3
(b)	I sleep very well.	1	2	3
(c)	I feel like crying.	1	2	3
(d)	I like to go out to play.	1	2	3
(e)	I feel like running away.	1	2	3
(f)	I get tummy aches.	1	2	3
(g)	I have lots of energy.	1	2	3
(h)	I enjoy my food.	1	2	3
(i)	I can stick up for myself.	1	2	3
(j)	I enjoy the things I do as much as I used to.	1	2	3
(k)	I have bad dreams.	1	2	3
(l)	I feel very lonely.	1	2	3
(m)	I am easily cheered up.	1	2	3
(n)	I feel so sad I can hardly stand it.	1	2	3
(o)	I feel very bored.	1	2	3

Q12. Tell us about your worries.

(1) Do you have any worries or concerns currenty? (circle the number applies.)

1. Yes.	2. No.

(a) To who answered “1. Yes” in (1).

Whom do you consult with? (circle all the numbers apply.)

1. Mother	11. Health teacher at school
2. Father	12. School social workers
3. Siblings	13. School counsellors
4. Grandparents	14. Counsellors (except for 13) including phone call
5. Other family members or relatives	15. Staffs at neighborhood center, community center or child center
6. Friends	16. Neighbors
7. Seniors or juniors	17. Internet consultation sites
8. School teachers	18. Others（Be specific：　　　　　）
9. School teachers at extracurricular activities	19. Not consult with any one
10. Teachers at cram school	

(2) Tell us how do you think or behave to your stressful events. Please choose the number closest to your current way of thinking or behaving currently.
(circle the number applies to each.)

	Not at all	Sometimes	Often	Always
a) Trying to change the current situation.	1	2	3	4
b) Cheer up by yourself.	1	2	3	4
c) Trying to see the positive side of things.	1	2	3	4
d) Leaving it as it is.	1	2	3	4
e) Asking others' help to solve the problems.	1	2	3	4
f) Thinking problems as not a big deal.	1	2	3	4
g) Trying to find out the cause of the problems.	1	2	3	4
h) Trying to explain the situation you are in.	1	2	3	4
i) Trying to assume the current experience beneficial.	1	2	3	4

This is the end of the question. Thank you for answering the questions.

One last time, make sure that there are no unfilled items and put it in a brown envelope, seal it, and then along with the white envelope of your gardians, please submit it to the school in a large envelope.

Questionnaire

Kochi Child Health Impact of Living Difficulty (K-CHILD) study

To gardians of second-year junior high school students

- This questionnaire is for the parents of the children to whom the questionnaire was distributed
- Survey is anonymous and contains any questions on the personal information that identifies the individual. All the answers will be statistically processed. Therefore, other people will not be able to find out who wrote what.
- Please fold the completed questionnaire into three, put into a white envelope, and seal it. Together with the child's brown envelope sealed in the same way, put them in a large envelope that was enclosed in the package when it was distributed. Close it up tightly with glue or tape, then submit it to school.
- The school or homeroom teacher will not open the envelope. Only those companies commissioned by the Kochi Children's Family Division at the Kochi Prefectural Office unseal the envelope.
- There are some personal inquiries about yourself or your family. Although you do not have to answer questions that is difficult to answer or questions that you do not want to answer, please tell us your answers as they are since the privacy is protected.
- The term "child" in this questionnaire signifies the child to whom a questionnaire has been distributed. Even if the child has siblings, it refers only those children to whom a questionnaire has been distributed.

-If you have any questions, please call us. -
Division of Children and Family Affairs of the Kochi Prefectural Government's Regional Welfare Department
Phone number: 088-823-9655
(Phone reception desk is open: Monday-Friday: 8:30 - 17:15)

❖ Excluding December 29th to January 3rd and holidays.

Q1. What is your relationship with the child. Please answer the relationship from the child's point of view.

1. Mother<br>2. Father<br>3. Mater's mother<br>4. Mater's father	5. Father's mother<br>6. Father's father<br>7. Siblings<br>8. Others（Be specific：　　　　）	9. Staffs of institution<br>（→To staff of institution, please answer the following questions as much as you can.）

Q2. Tell us the current marital status of the child's parents.

1. Married<br>2. Divorced	3. Bereaved<br>4. Unmarried

Q3. Who are living with the child with? Please tell us each number and the total number. Please include those who are living outside of home due to business.

1. Mother	5. Father's mother	9. Younger brother （　　people）
2. Father	6. Father's father	10. Younger sister （　　people）
3. Mother's mother	7. Older brother （　　people）	11. Other relatives （　　people）
4. Mother's father	8. Older sister （　　people）	12. Others （　　people）

→（　　　　　）**people in total * please write the number including you and the child**

Q4. Tell us about your residence.

(1) Where are you living currently?

1. Outside of Kochi Prefecture　　2. Inside of Kochi Prefecture; the name of cities（　　　　　　）

(2) How many times have you moved after the child were born?

(a) Before entering the elementary school	（　　　）times
(b) During the elementary school	（　　　）times
(c) After entering the junior high school	（　　　）times

Q5. Please tell us all the institutions your child has attended before entering elementary school.

1. Certified nursery schools<br>2. Uncertified nursery schools<br>3. Certified child care center	4. Kindergarten<br>5. There is no institution

Q6. Tell us about after-school class in school *.

*** the extracurricular class after school or during long vacation in school.**

(1) If there is after-school in the school the child go to, do you want to let the child join the class?

1. Yes	2. No	3. I do not know

(2) What do you expect in after-school class?

1. Establishing basic academic skills
2. Developing study habits
3. Further developing the current academic skills
4. Overcoming challenges through detailed guidance in small groups
5. Helping studying during long vacations
6. Other（Be specific：　　　　　　　　　　　　）
7. I do not know

(3) If there is supporting staffs coming outside of school in after-school class, what do you expect in the supporting staffs?

1. Continuing to support from the class
2. Having the ability to develop the academic skills
3. Continuing to stay for one year
4. Teaching in collaboration with teachers
5. Possessing teaching licenses
6. Others（Be specific：　　　　　　　　　　）
7. I do not know

Q7. Tell us about your future education.

(1) Untile which level of education are you willing to let your child to get?

1. To junior high school	3. To vocational school *	5. To university or higher
2. To high school	4. To technical collage or university	6. I do not know

*This school is the one you are going to after graduating high school.

(2) Why is your reason of your anwer in (1)?

1. That is what my child wants to.
2. I think most people would go to that level of school.
3. I think that level is enough.
4. Considering my child’s academic ability.
5. Lack of financial resources at home.
6. Others（Be specific：　　　　　　　　　　　　　　　　　　　　　　　　）
7. There is no specific reason.

Q8. Which describes your child’s health condition the most?

1. Good	2. Somewhat good	3. Normal	4. Not very good	5. Not good

Q9. Tell us about your child's teeth condition.

Does your child have any cavities currently/ have your child ever had any cavities in the past?

1. Yes →（ teeth）	2. Yes（treatment is completed） →（ teeth）	3. No	4. I do not know

(a) To who answered "1. Yes" in Q8.

Does your child currently take any treatment?

1. Yes	2. No	3. I do not know

(b) To who answered "2. No" in (a).

What is the reason your child does not take any treatment although he/she has cavities.

1. Cannot pay treatment fees.
2. Since the child refused to go.
3. The dentist is located too far from home to go.
4. I am too busy to take the child to dentist.
5. Others（Be specific： ）

Q10. Have you ever thought your child should be seen by doctors but did not actually have your child seen within the last one year?

1. Yes	2. No

(a) To who answered "1. Yes" in Q9. What is the reason of that? Please choose the answer most closest to your situation.

1. I could not afford payments since I did not have public medical insurance.
2. I do not think I could afford the co-payments at hospital although I have joined public medical insurance.
3. Your child did not want to go to hospital.
4. The hospital is located too far to go to.
5. I was too busy to bring my child to hospital.
6. I thought I should let my child seen by doctors but on second thought I could not find any necessities.
7. My health condition was too but to bring my child to hospital.
8. Others（Be specific： ）

Q11. Tell us about your child's condition on vaccination.

*** Regular vaccination (Tubeculosis (BCG), Measles, Rubella, Polio, etc.) is free of charge. Optional vaccination (Flu, Mumps, etc.) is for fees.**

	Vaccinated	Not vaccinated	I do not know
(1) Regular vaccination	1	2	3
(2) Optional vaccination (Flu) * the past one year	1	2	3

(3) Optional vaccination (Mumps)	1	2	3

(a) To who answered "2. Not vaccinated" in Q10 about regular vaccination.
What is the reason you do not let your child take?

1. I did not know it is for free.
2. My child did not want to be vaccinated.
3. The hospital is located too far to go to.
4. I was too busy to bring my child to hospital.
5. I thought I did not need to let my child vaccinated considering the child's condition.
6. I did not know where and when the child should be vaccinated.
7. My child had a fever at the timing of vaccination.
8. My health condition was too bad to bring child to hospital.
9. Others（Be specific：　　　　　　　　　　　　　　　　　　　　）

Q12. Choose the number closest to the condition of the child during the past one month.

	Exactly true	Almost true	Somewhat true	Rarely true	Not true at all
(1) The child could say good thing about the future.	1	2	3	4	5
(2) The child tried to do her/his best.	1	2	3	4	5
(3) The child could cope well with insults and mean comments.	1	2	3	4	5
(4) The child could greet a person in a polite way.	1	2	3	4	5
(5) The child could offer to help somebody.	1	2	3	4	5
(6) The child could seek advice when it is necessary.	1	2	3	4	5
(7) The child could pass up something he/she wanted, or do something he/she did not like, to get something better in the future.	1	2	3	4	5
(8) The child could ask questions to clarify what he/she did not understand.	1	2	3	4	5

Q13. Please choose the number closest to the conditions to each of the following sections regarding your child's behavior during the past 6 months.

Please answer all the following questions even if you are unsure.

	True	Somewhat true	Certianly true
(1) Considerate of other people's feelings.	1	2	3
(2) Restless, everactive, cannot stay still for long.	1	2	3
(3) Often complains of headaches, stomachaches or sickness.	1	2	3
(4) Shares readily with other children (treats, toys, pencils etc.)	1	2	3
(5) Often has temper tantrums or hot tempers.	1	2	3
(6) Rather solitary, tends to play alone.	1	2	3
(7) Generally obedient, usually does what dults request	1	2	3
(8) Many worries, often seems worried.	1	2	3
(9) Helpful if somone is hurt, upset or feeling ill.	1	2	3
(10) Constantly fidgeting or squirming.	1	2	3
(11) Has at least one good friend.	1	2	3
(12) Often fights with other children or bullies them.	1	2	3
(13) Often unhappy, down-hearted or tearful.	1	2	3
(14) Generally liked by other children.	1	2	3
(15) Easily distracted, concentration wanders.	1	2	3
(16) Nervous or clingy in new situations, easily loses confindence.	1	2	3
(17) Kind to younger children.	1	2	3
(18) Often lies or cheats.	1	2	3
(19) Picked on or bullied by other children.	1	2	3
(20) Often volunteers to help others (parents, teachers, other children).	1	2	3
(21) Thinks things out before acting.	1	2	3
(22) Steals from home, school or elsewhere.	1	2	3
(23) Gets on better with adults than with other children.	1	2	3
(24) Many fears, easily scared.	1	2	3
(25) Sees tasks through to the end, good attention span.	1	2	3

Q14. Tell us about health condition of the child's mother and father.

*** Please write answer separately for both parents in your response form. If the parent is absent, please leave your response blank.**

(1) Tell us about the ages, heights, and weights of the child's mother and father.

* The response to this question will be used to find the correlations with physique of the child. Please respond as accurately as possible.

Mother		Father	
Age	( ) years old	Age	( ) years old
Height	( ) cm	Height	( ) cm
Wight	( ) kg	Wight	( ) kg

(2) Do the child's mother and father smoke habitually. Or, Have they ever smoked?

Mother	Father
1. Currently smoking habitually.	1. Currently smoking habitually.
2. Smoked before but is not currenly.	2. Smoked before but is not currenly.
3. Have never smoked before.	3. Have never smoked before.

→ To who answered "1. Currently smoking habitually." And "2. Smoked before but is not currently." In (2).

(a) Do they smoke/ have they smoked inside of house?

Mother	Father
1. Yes	1. Yes
2. No	2. No

(b) Do they smoke/ have they smoked in front of the child?

Mother	Father
1. Always	1. Always
2. Sometimes	2. Sometimes
3. Never	3. Never

Q15. Tell us about the occupation and experience of the child's mother and father.

*** Please write answer separately for both parents in your response form. If the parent is absent, please leave your response blank.**

(1) What is the current occupation of the child's mother and father?

Mother	Father
1. Company executives	1. Company executives
2. Full-time employees of a private company	2. Full-time employees of a private company
3. Regular employees such as civil servants	3. Regular employees such as civil servants
4. Contract, temporary, and part-time employees	4. Contract, temporary, and part-time employees
5. Part-time employees	5. Part-time employees
6. Self-employed (including family business)	6. Self-employed (including family business)
7. Free enterprise	7. Free enterprise
8. Staff members	8. Staff members
9. Working in a way other than 1 to 8	9. Working in a way other than 1 to 8
10. Full-time housewife	10. Full-time housewife
11. Student	11. Student
12. Retired	12. Retired
13. Seeking employment	13. Seeking employment
14. Other unemployed	14. Other unemployed

(2) To who answered "1" to "9" in (1).

(a) Are you allowed to return home from work if the child got sick suddenly.

Mother	Father
1. Can go back immediately	1. Can go back immediately
2. Can go back	2. Can go back
3. Cannot go back easily	3. Cannot go back easily
4. Cannot go back ever	4. Cannot go back ever

(b) How many hours does the child's mother and father work a week? * if they work in several workplaces, please sum up all the working time.

Mother	( ) hours a week on average
Father	( ) hours a week on average

(c) Please choose the time the child's mother and father usually arrive home. * if they work at home, please choose the time you finish to work.

Mother	Father
1. Until 6 pm	1. Until 6 pm
2. 6pm to 8pm	2. 6pm to 8pm
3. 8pm to 10pm	3. 8pm to 10pm
4. After 10pm (including early morning)	4. After 10pm (including early morning)
5. There is no fixed time	5. There is no fixed time

(3) Tell us about the last school the child's mother and father went/ is going.
(if they graduated high school at the highest, please choose "3. Graduated high school"; if they dropped out of school, please choose "2. Dropped out of high school".)

Mother	Father
1. Graduated junior high school	1. Graduated junior high school
2. dropped out of high school	2. dropped out of high school
3. Graduated high school	3. Graduated high school
4. Graduated a vocational school (after high school)	4. Graduated a vocational school (after high school)
5. Graduated a junior college/ technical college	5. Graduated a junior college/ technical college
6. Dropped out of university	6. Dropped out of university
7. Graduated university	7. Graduated university
8. Graduated graduate university	8. Graduated graduate university
9. Others（　　　　　）	9. Others（　　　　　）
10. I do not know	10. I do not know

Q16. Tell us child's home economics.

(1) How do you feel about the current living conditions?

1. Very affordable	3. Normal	5. In very financial difficulty
2. Somewhat affordable	4. In financial difficulty	

(2) How do you rate of your family's social status within a whole society?

1. High	3. Middle	5. Low
2. Middle-high	4. Middle-low	6. I do not know

(3) Among the following items, is there anything that you do not have due to financial reasons.

1. A washing machine	8. A computer connected to the internet
2. A rick cooker	9. A bathtub for household
3. A vaccuum	10. A bed per person
4. A hearter/ hearting appliance	11. A curtain
5. An air-conditioner	12. A car
6. A microwave	13. A savings (more than 50,000 JPY)
7. A phone (including both landlines and mobiles)	14. There is nothing applicable

(4) Among the following items, is there anything that you could not pay or buy due to financial reasons?

	Could not pay/buy	Could pay/buy	Not applicable (no need to pay/buy)
1. School field trips/ extracurricular activities	1	2	3
2. School textbooks	1	2	3
3. Rent	1	2	3
4. Housing loans	1	2	3
5. Electricity bills	1	2	3
6. Gas bills	1	2	3
7. Water bills	1	2	3
8. Phone bills (including both lanllines and moblies)	1	2	3
9. Insurance fees for public pension, national health insurance, and/or public nursing care	1	2	3
10. Bus or train fees for commuting	1	2	3
11. Food	1	2	3
12. Clothes or bags necessary for child	1	2	3

(5) Tell us about the living costs and educational fees for the child whom the questionnaire was distributed to. How much do you spend to the following each item per month? Please answer the average cost.

* If there is no cost, please fill “0” in the parentheses.

	Monthly cost
(a) Tuition/ school fees	Approximately（ ）JPY
(b) Extracurricular educational fees such as cram school	Approximately（ ）JPY
(c) Lesson fees (excluding sport club)	Approximately（ ）JPY
(d) Sport club	Approximately（ ）JPY
(e) Allowances	Approximately（ ）JPY
(f) Mobile phone and smart phone bills	Approximately（ ）JPY

(6) Tell us about the cost you spent when the child entered high school (Admission fees, payment for clothes and bags, shoes and teaching materials). Did you feel it is difficult to pay?

1. Difficult	2. Somewhat difficult	3. Not so much difficult	4. Not difficult

(a) To who answered “1. Difficult” or “2. Somewhat difficult” in (6).

How did you deal with it? Please choose the closest answers.

1. Made ends meet by saving money.<br>2. Got a few second-hand items.<br>3. Borrowed money from friends and relatives.<br>4. Borrowed money from local municipalities.	5. Borrowed money from credit cards.<br>6. Could not prepare before entering.<br>7. Others（Be specific：　　　　　）

(7) Are you receiving scholarship for high school students in your household for the sake of the child?

* Scholarship for high school students is available to household that are exempt from tax on income and aims to support educational fees except for tuition fees.

1. Receiving	2. Not receiving	3. I do not know

(a) To who answered “2. Not receiving” in (7). What is the reason you are not receiving it?

1. Not applied (necessary but not meet the requirement)
2. Not applied (necessary but not know how to apply it)
3. Not applied (necessary but feel resistence to application)
4. Not applied (feel no necessities)
5. Applied but not approved
6. Not knowing the scholarship for high school students
7. Others（Be specific：　　　　　）

(8) How much of the following public pension and social security benefits is your household receiving at any one time?

If there is any corresponding income, please fill "0" in the parentheses.

It includes any pension/ benefits related to siblings.

Child support allowance is the allowance for household whose income is below the threshold and has only one parent.

	Payment at a time
(a) Child support allowance	( ) JPY （Payed once per four months）
(b) Special child support allowance	( ) JPY （Payed once per four months）
(c) Annuities (survivor's pensions, age pensions)	( ) JPY （Payed once per two months）
(d) Public Allowance	( ) JPY

(9) Tell us about the income excluding public pension and social security benefits you described in (8).

How much is the approximate total annual income of all the members who share the livelihood?

* income includes income from work (including part-time jobs), business income (self-employment, etc.), agricultural income, real estate income, interest and dividends and personal pensions. Please exclude the income from public pension and social security you answered in (8).

* If there is several income resources (income from work by father, business income by mother for example), please tell us the approximate total income.

1. There is no income (0 JPY)	5. 2.00 – 2.99M JPY	9. 6.00 – 6.99M JPY
2. 1 - 49,999 JPY	6. 3.00 – 3.99M JPY	10. 7.00 – 7.99M JPY
3. 50,000 – 99,999 JPY	7. 4.00 – 4.99M JPY	11. 8.00 – 8.99M JPY
4. 1M – 1.99M JPY	8. 5.00 – 5.99M JPY	12. More than 9.00M JPY
		13. I do not know

Q17. Tell us the relationship between the child and family members including you.

(1) Do you do the following activities in your family?

	Almost every day	3-4 times a week	1-2 times a week	1-2 times a month	Rarely
a) Helping the child's study	1	2	3	4	5
b) Exercising with the child	1	2	3	4	5
c) Talking about school activities with the child	1	2	3	4	5
d) Talking about social/political/economical problems with the child	1	2	3	4	5
e) Talking about TV (excluding news) with the child	1	2	3	4	5
f) Cooking with the child	1	2	3	4	5
g) Going out with the child	1	2	3	4	5

(2) Do you have opportunities to think or talk about the child's future plan (such as dreams, carrer path, and occupation) with the child in your family?

1. Often do	2. Sometimes do	3. Not so much	4. Have never done before

(3) Do you have high expectations for your child's potential in your family?

1. Have high expectations	3. Have few expectations	5. I do not know
2. Have a few expectations	4. Have no expectations	

(4) How often do you cook your child's meal in your family? (including easy meal like stirred eggs)

1. Almost every day	4. A few days a month (on weekeds/ holidays)
2. 4 to 5 days a week	5. Rarely cook
3. 2 to 3 days a week	

(5) Do you do the following activities to the child in your family?

	Yes, we do	No, we do not	
		Do not want to（Not willing to）	Cannot afford
(a) Celebrating the birth day	1	2	3
(b) Going to family trip once a year	1	2	3
(c) Giving Christmans present/ new year's gift (e.g., money)	1	2	3

(6) Do you do the following reactions to the child in your family?

	Often	Sometimes	Parely	Never
a) Hitting the child's body (e.g., bottom, hands, head, face, etc.)	1	2	3	4
b) Yelling at the child	1	2	3	4
c) Beating the child	1	2	3	4
d) Shutting the child outside	1	2	3	4
e) Neglecting the child	1	2	3	4
f) Not feeding the child	1	2	3	4
g) Insulting the child repeatedly	1	2	3	4
h) Leaving the child alone in the house at night	1	2	3	4
i) Having a big fight in front of the child	1	2	3	4

(7) **<u>To whose child is not living together with grandparnts.</u>**

(a) How many hours does it take to go to grandparents' house? Please tell us how to go usually and how many hours to take.

① To the mother's parents' house

1. on foot	2. By bike	3. By car	4. By bus	5. By train	6. By air plane	(　　) Hours (　　) Minutes

② To the father's parents' house

1. on foot	2. By bike	3. By car	4. By bus	5. By train	6. By air plane	(　　) Hours (　　) Minutes

(b) How much help of child rearing do you receive from the child's grandparents?

Parents of the mother	Parents of the father
1. On a daily basis	1. On a daily basis
2. Frequently	2. Frequently
3. Sometimes	3. Sometimes
4. Rarely	4. Rarely
5. I do not know	5. I do not know

Q18. Tell us about you, who are answering this questionnaire.

(1) Do you think you are happy? When we set "very happy" as 10-point and "very unhappy" as 0-point, what score do you give?

Very unhappy	Very unhappy
0---1---2---3---4----5---6---7---8---9---10	

(2) How long have you lived in the current community?

(   ) years (   ) months

(3) Do you socialize with your neighbors in your family?

1. Having a very close relationship.	3. Socializing but do not have close relationship.
2. Having a close relationship.	4. Almost no/ no relationship.

(4) How often do you receive food from or share food with people in your community?

Receving food	Sharing food
1. On a daily basis	1. On a daily basis
2. Frequently	2. Frequently
3. Sometimes	3. Sometimes
4. Rarely	4. Rarely
5. Never	5. Never

(5) Are you belonging to some groups, sport club, or private organizations such as NPO, and neighborhood associations, and doing any activities?

1. Yes	2. No

(6) Do you have any person you can consult with if you are in trouble or having any worries?

1. No	2. 1 to 2 people	3. 3 to 4 people	4. 5 to 7 people	5. More than 7 people

(a) To who answered any numbers except for “1. No” in (6).

Who/where is that?

1. Partners	5. Friends living nearby
2. Own parents	6. Friends living far
3. Partners’ parents	7. Staff at work places
4. Siblings/ other relatives	8. Others（Be specific：　　　　　）

(7) Have you ever consulted with the following public institutions? If not, please choose the most suitable resons.

	Yes, I have consulted.	No, I have never consulted.			
		Have never feel like to consult with.	Want to consult but felt resistant.	Time and place was not suitable.	I did not know how to consult.
1. Municipal offices (including welfare offices, health center, etc.)	1	2	3	4	5
2. Child and family support center	1	2	3	4	5
3. Teachers in schools, nursery schoos and kindergartens	1	2	3	4	5
4. School cousellors	1	2	3	4	5
5. School social workers	1	2	3	4	5
6. Demogracy and childre's committee members	1	2	3	4	5
7. The county welfare and health department	1	2	3	4	5
8. Child guidance centers	1	2	3	4	5
9. Police	1	2	3	4	5
10. Labour information centers	1	2	3	4	5
11. Consultation sites on the internet	1	2	3	4	5
12. Other public institutions	1	2	3	4	5

(8) How do you feel about your health condition?

1. Good	2. Somewhat good	3. Normal	4. Not very good	5. Not good

(9) During the past thirty days, about how often did you feel…

	All of the time	Most of the time	Some of the time	A little fo the time	None of the time
a) … nervous?	1	2	3	4	5
b) … hopeless?	1	2	3	4	5
c) … restless or fidgety?	1	2	3	4	5
d) … so depressed that nothing could cheer you up?	1	2	3	4	5
e) … that everything was an effort?	1	2	3	4	5
f) … worthless?	1	2	3	4	5

(10) Have you ever experienced the following events during childhood?

1. My mother died.	5. My parnets played violence to me.
2. My father died.	6. I was neglected (diets, clothes).
3. My parents divorced.	7. My parents said something hurt me.
4. My father played violence to my mother.	8. We experienced financial hardships.

(11) How do you rate of your family's social status within a whole society in your childhood?

1. High	3. Middle	5. Low
2. Middle-high	4. Middle-low	6. I do not know

Q19. Tell us about how you collect the information and the conditions that you utilize the support.

(1) Where do you get the information on the child's education, health and medicine?

Please choose the main three sources.

1. Partner	10. SNS (facebook, Twitter, LINE, etc.)
2. My family or relatives	11. Internet or blog
3. My friends and neighbors	12. Municipal publicity
4. Teachers in school	13. News papers
5. Teachers in club activities	14. Books or magazines
6. Municipal survice office	15. Others（Be specific： ）
7. Health center	16. There is no sources.
8. Hospital	
9. Television/ radio	

(2) Have you ever received any support for the child, whom the questionnaire was distributed, from the following resources?

If not, please choose the most suitable reasons.

	I have ever used.	I have never used.					
		I did not feel like to be supported.	I want to use but did not know how to use.	Time or other system was not usable.	I do not know how to use it.	I do not know about the system.	There is no system available.
a) Community child rearing support center	1	2	3	4	5	6	7
b) Short-term childcare support center	1	2	3	4	5	6	7
c) Family support center	1	2	3	4	5	6	7
d) Children's cafeteria	1	2	3	4	5	6	7
e) Food assistance by food bank	1	2	3	4	5	6	7
f) A place where children in junior high school and above can spend their free time	1	2	3	4	5	6	7
g) Educational support from school	1	2	3	4	5	6	7
h) Educational support outside of school	1	2	3	4	5	6	7

＜references＞

(a) Community child rearing support center	A place where parents/ guardians of children between the ages of 0 to 3 years old can come and talk about their concerns and worries while enjoying a relaxing time together with their children.
(b) Short-term childcare support center	If you are having trouble taking care of your child at the time of hospitalization, childbirth, nursing care or work, etc., you can leave your child in a child welfare institution.
(c) Family support center	The system registers both those who want to help with childcare and those who want to help with childcare so that they can balance work and childcare and give their families more time to raise their children through support activities.
(d) Children's cafeteria	A place where private organizations provide free or discounted meals to children for the purpose of supporting their food.
(e) food bank	Activities in which NPOs and other organizations retrieve food from food manufacturers and provide it to welfare facilities and people in need of support, free of charge, for food that would otherwise be disposed of when it is still edible.

(a) Are you currently interested in using the following support system?

1. Community child rearing support center
2. Short-term childcare support center
3. Family support center
4. Children's cafeteria
5. Food assistance from food bank
6. A place where children in junior high school and above can spend their free time
7. Educational support from school
8. Educational support outside of school

(3) If there is a system that you can leave the child until the mother and father return from work after the time the child can stay in the current support system finished (approximately until 8pm)?

1. Want to use
2. Do not want to use
3. I do not ned to use
4. I do not know
5. Other（Be specific：　　　　　　）

(4) Have you ever used the following support system in your family? If not, please choose the most suitable rasons.

	I have used.	I have never used.				
		I have never felt like using the system (at the first place, I was not eligible to the system.)	I would like to use but did not know how to use it.	I would like to use but it was difficult to use.	I did not know how to use it.	I did not know anyting about the system.
a) Public welfare funds	1	2	3	4	5	6
b) Public Assistance	1	2	3	4	5	6
c) Mother/ father and child welfare fund	1	2	3	4	5	6
d) Child support allowance	1	2	3	4	5	6

＜References＞

a) Public welfare funds	Low-interest or interst-free loans for households with income below a certain level. You can contact with your local social welfare council.
b) Public Assisntace	If you have no other way to pay for living expenses or medical care due to illness or unemployment, etc. and have no other choice, you may be able to get a living allowance under certain conditions. This system provides assistance in eight areas: education, housing, medical care, nursing care, childbirth, occupation and funerals. Please contact your local government office for assistance.
c) Mother/ father and child welfare fund	A low-interest or interest-free loan for single-parent households. Please contact your local government office.

d) Child support allowance	An allowance to support single-parent households with incomes below a certain level.<br>Municipal offices are open to the public.

<u>This is the end of the question. Thank you for answering the questions.</u>

One last time, make sure that there are no unfilled items and put it in a brown envelope, seal it, and then along with the white envelope of your gardians, please submit it to the school in a large envelope.

Questionnaire

Kochi Child Health Impact of Living Difficulty (K-CHILD) study

To second-year junior high school students

Purpose of the survey

- This is the questionnaire aiming to find out the dreams, worries and living situations of the children in Kochi Prefecture.

A request to you

- Please complete this servey on your own. There is no need for your family to see your answer.
- Please do not write down your name.
- You do not need to questions that you would not like to answer. Also, there are no wrong or correct answers. Please write your thoughts on this question.
- When you are asked about "parents" in the question, please respond as a parent or a guardian of you.

How to submit your questionnaire

- When you have finished answering, fold it into three and put it in a brown envelop by yourself. Then close it with glue or tape.
- Put your envelope and your guardian's envelope in a large envelope together.
- Close the large envelope tightly with glue or tape, and take it to school.

-If you have any questions, please call us. -
Division of Children and Family Affairs of the Kochi Prefectural Government's Regional Welfare Department
Phone number: 088-823-9655
(Phone reception desk is open: Monday-Friday: 8:30 - 17:15)

- ❖ Excluding December 29th to January 3rd and holidays.

Q1. Please tell us about your gender and date of birth.

(1) What is your gender? (circle one of them.)

1. Male 2. Female

(2) When is your birth day?

(please answer with a number in parentheses.)

Year ( ) Month ( )

Q2. Please tell us your height and weight. (please answer with a number in parentheses.)

(1) Height	( ) cm
(2) Weight	( ) kg
(3) Month of measurement	Year ( ) Month ( )

Q3. Do you have any of the following items? Please choose the one that is closest to your situation. (please circle one of the numbers that apply to each.)

	I have.	I do not have	
		I want to have but do not.	I do not want.
(1) My own book (except for textbooks)	1	2	3
(2) My own room (including shared room with your siblings)	1	2	3
(3) A computer connected to the internet at home	1	2	3
(4) The place where you can study at home	1	2	3
(5) My own study desk	1	2	3
(6) Sport equipments (the glove for baseball, soccer ball, etc.)	1	2	3
(7) Electronic gaming device	1	2	3
(8) Bicycle	1	2	3
(9) Allowance to buy some snacks and goods	1	2	3
(10) A cell phone, a smart phone	1	2	3
(11) Portable music player etc.	1	2	3

Q4. Do you have an occupation that you want to be in the future?

1. Yes	2. No

(a) <u>To who answered "no" in Q4.</u>

What is the reason you do not have any occupation that you want to be?

1. I am satisfied in the current situation alrealy.	3. I cannot think of anything specific.
2. I do not think I am able to be what I want to be.	4. I do not know.

Q5. Which level of education do you want to get in the future?

Please choose the answer closest to your hope.

1. To junior high school<br>2. To high school<br>3. To vocational school *	4. To technical college or university<br>5. To university or higher<br>6. I do not know

*This school is the one you are going to after graduating high school.

Q6. Please tell us about your friends and family

(1) How many friends like the following do you have in total? If you do not have, please answer “0”.

(please answer with a number in parentheses.)

a) A good friend in a same school （ ） people

b) A good friend in a different school （ ） people

c) A friend you can consult with if you have any worries （ ） people

(2) How do you think about the relationship with the friends of yours?

		Strongly agree	Agree	Not agree	Disagree
a)	I think I am playing with my friends so much.	1	2	3	4
b)	I think I am getting along with my friends.	1	2	3	4
c)	I think my friends like me.	1	2	3	4
d)	I think I am different from other friends.	1	2	3	4

(3) How much influence do you think does your opinions and behaviors have on your classmates?

1. Very much	2. To some extent	3. Not very much	4. Not at all

(4) Please tell us about your relationship with your parents. Please choose every answer apply.

1. Talking about school events and my friends.<br>2. Talking about my future and carrer.<br>3. Talking about social events and news.	4. Helping me learn.<br>5. Listening to me.

Q7. Please tell us about your daily life.

(1) **With whom** do you usually spend time after school **during weekdays.** (circle one of the numbers applies.)

1. Your family (including grandparents and relatives)
2. Adults except for your family (neighbors, teachers at a cram school, coaches at a sport club, etc.)
3. Friends of my school
4. Fiends of the different school (friends you met at a local sport club, neighbors, friends from the same elementary and junior high school)
5. Coworkers such as part-time jobs
6. Spending alone

(2) Where do you spend your time after school on weekdays and for how many days a week? (circle the number applies to each.)

		Every day	3 to 4 days a week	1 to 2 days a week	Never
(a)	Home	1	2	3	4
(b)	Grandparents' or relative's house	1	2	3	4
(c)	Friend's house	1	2	3	4
(d)	Extracarricular activities or cram school	1	2	3	4
(e)	Extracarricular activities at school	1	2	3	4
(f)	Sport club（base ball stadium, soccer stadium, etc.）	1	2	3	4
(g)	Work places for part-time jobs	1	2	3	4
(h)	Park	1	2	3	4
(i)	Library	1	2	3	4
(j)	Restaurant, shopping street or supermarket	1	2	3	4
(k)	Game center	1	2	3	4
(l)	Others（Be specific：　　　　　　　　）	1	2	3	4

(3) Which place are you relaxed the most from (a) to (i) in (2)? Please choose the one and write down the alphabet in the parenthesis. If you do not have place you are relaxed, please circle "no, there is no place I feel relaxed."

Alphabet（　　　　　　）	No, there is no place I feel relaxed.

(4) How long do you spend time for each activity? (circle the number applies to each.)

		More than 2 hours a day	1 to 2 hours a day	Less than 1 hour a day	4 to 5 days a week	2 to 3 days a week	One day a week	Merely/ never
(a)	Playing with a computer game	1	2	3	4	5	6	7
(b)	Watching TV or DVDs	1	2	3	4	5	6	7
(c)	Using a cell phone, tablet or smart phone	1	2	3	4	5	6	7
(d)	Reading a book	1	2	3	4	5	6	7
(e)	Indoor activity except for (a) to (d)	1	2	3	4	5	6	7
(f)	Houseworks (laundry, clearning, cooking, tyding up, etc.) or supporting your siblings.	1	2	3	4	5	6	7
(g)	Exercise or physical activities	1	2	3	4	5	6	7

(5) How many books did you read within the last one month? (circle the number applies.)

※Please count the books you read outside of home. Also, count the electronic books that you can read with a laptop, tablet or smartphone. Please exclude any journals or comics regardless of their contents.

1. Did not read	3. 2 to 3 books	5. 8 to 11 books
2. 1 book	4. 4 to 7 books	6. More than 11 books

(6) How long do you sleep on weekdays? (circle the number applies.)

1. More than 10 hours	3. 8 to 9 hours	5. 6 to 7 hours
2. 9 to 10 hours	4. 7 to 8 hours	6. Less than 6 hours

(7) What time do you wake up and go to sleep? (circle the number applies to each.)

Time you wake up	Weekdays	Weekends
Earlier than 6 am	1	1
6 am to 7 am	2	2
7 am to 8 am	3	3
8 am to 9 am	4	4
9 am to 10 am	5	5
Later than 10 am	6	6

Time you go to sleep	Days except for Fri. and Sat.	Fridays and Saturdays
Ealier than 8 pm	1	1
8 pm to 9 pm	2	2
9 pm to 10 pm	3	3
10 pm to 11 pm	4	4
11 pm to 12 pm	5	5
Later than 12 pm	6	6

Q8. Please tell us about your diet and health.

(1) Do you usually eat breakfast everyday on weekdays? (circle the number applies.)

1. Every day (5 days a week)	3. Few days (1 to 2 days a week)
2. Most days (3 to 4 days a week)	4. Not eat

(a) To who answered “2. Most days”, “3. Few days”, “4. Not eat” in (1). Which of the reasons is closest to your reason why you do not eat breakfast? (circle the number applies.)

1. There is no time to eat.	4. There is no specific reason.
2. The meal is not ready.	5. Others（Be specific： ）
3. There is no appetite in the morning.	

(2) With whom do you eat breakfast on weekdays? (circle all the numbers apply.)

1. Family members (parents)	4. Eating alone
2. Family members (siblings)	5. Do not eat breakfast
3. Other family members (grandparents, etc.)	6. Others（Be specific： ）

(3) With whom do you eat supper on weekdays? (circle all the numbers apply.)

1. Family members (parents)	4. Eating alone
2. Family members (siblings)	5. Do not eat breakfast
3. Other family members (grandparents, etc.)	6. Others (Be specific： ）

(4) How much do you usually eat/ drink the following foods/ drinks except for school meal?

(circle the number applies to each.)

		Evey day	4 to 5 days a week	2 to 3 days a week	Less than one day a week	Do not eat/ drink
(a)	Vegetable	1	2	3	4	5
(b)	Fruit	1	2	3	4	5
(c)	Meat or fish	1	2	3	4	5
(d)	Cup and instant noodles	1	2	3	4	5
(e)	Rice balls and bento boxes bought at convenience stores	1	2	3	4	5
(f)	Junk food	1	2	3	4	5
(g)	Snacks	1	2	3	4	5
(h)	Juices	1	2	3	4	5

(5) What do you eat first at a meal? (circle the number applies.)

(1) Rice or bread (staple foods)	(3) Vegetable	(5) Do not decide
(2) Meal or fish	(4) Soup	(6) Do not know

(6) Are you currently on a diet? (circle the number applies.)

1. Yes.	2. Not currently but was on a diet in the past.	3. No.

↓

a) To who answered "1. Yes", "2. Not currently but was on a diet in the past" in (6). What are you doing/ have you done to reduce your weight? (circle all the numbers apply.)

1. Reducing the amount of meal.	6. Calculating calory intakes.
2. Cutting back on snacks and evening meals.	7. Doing exercise.
3. Skipping a meal.	8. Taking a medicine.
4. Eating only certain foods (e.g., vegetables, tofu).	9. Spitting out what you eat.
5. Not eating certain foods (e.g., carbohydrate such as rice or bread).	10. Others（Be specific：　　　　　　　）

(7) Do you cook by yourself or help cooking at home? (circle the number applies.)

1. Almost every day (more than 3 days a week)	2. Often (1 to 2 days a week)
3. Sometimes (1 to 3 days a month)	4. Never

(8) How do you feel about your health condition? (circle the number applies.)

1. Good	2. Somewhat good	3. Normal	4. Not very good	5. Not good

(9) How many times do you brush your teeth a day? (circle the number applies.)

1. More than two times a day	2. One time a day	3. Not brushing every day

(10) How long do you usually brush your teeth a time? (circle the number applies.)

1. Less than 1 minute	2. 1 to 2 minutes	3. 3 to 5 minutes	4. More than 5 minutes

(11) Approximately how many cavities do you have right now? Please include the cavities currently under treatment.

* please write down “0” if you do not have any cavity. Please do no include the cavities complete the treatment.

（　　　　）teeth

(a) To who answered any number except for “0” in (11) (who have any cavities curreny).

Are you currently under treatment of cavities? (circle the number applies.)

1. Yes	2. No	3. I do not know

↓

(b) To who answered “2. No” in (a).

What is the reason? (circle the number applies.)

1. Because treatment at dentist is painful.
2. Because treatment at dentist is scary.
3. Because there is no time to go to dentist.
4. Because there is no dentist nearby.
5. Because cavities do not hurt.
6. Because I have never been to dentist.
7. Because parents do not bring me to dentist.
8. Other reasons（Be specific：　　　　　　　　　　　　　　　　　　　　）

Q9. Please tell us about your school and study.

(1) Which statement is closest to the situation about you and your school? (circle one the numbers apllies.)

	Do not agree at all.	Not agree so much.	Neigher yes or no.	Agree to some extent.	Agree so much.
(a) I like the atmosphere at class.	1	2	3	4	5
(b) I like my school teacher.	1	2	3	4	5
(c) I enjoy school life.	1	2	3	4	5
(d) I say hello to school teachers and classmates.	1	2	3	4	5
(e) I trust my teacher.	1	2	3	4	5
(f) I trust my classmates.	1	2	3	4	5
(g) I actively participate in the school events.	1	2	3	4	5
(h) I like to meet my teacher.	1	2	3	4	5
(i) I like to meet my classmates.	1	2	3	4	5

(2) Do you understand your school lessons well? (circle one of the numbers apllies.)

1. Understand all the lessons.<br>2. Understand most of the lessons.	3. Do not understand much.<br>4. Not understand most of the lessons.	5. Not understand almost all the lessons.

(3) Whom do you ask to help when you have questions about your study? (circle the number applies.)

1. Your parents<br>2. Other family members (siblings, grandparents, etc.)<br>3. School teachers<br>4. School teachers at extracurricular activities	5. Teachers at cram school<br>6. Other adults<br>7. Friends of yours<br>8. There is no one to ask

(4) How high do you think your grades are in your class? (circle the number applies.)

1. High<br>2. Middle-high	3. Middle<br>4. Middle-low	5. Low<br>6. I do not know

(5) What subject are you good at? (circle all the numbers apply.)

1. Math<br>2. Language<br>3. Science<br>4. Society	5. English<br>6. P.E.<br>7. Art<br>8. Information	9. Home economics<br>10. Vocational training<br>11. None is applicable.

(6) How long do you study except for class lessons. Please tell us your study hours a day. (circle the number applies.) * please include the time you spend at cram school.

1. Not at all.<br>2. Less than 30 minutes.<br>3. 30 minutes to 1 hours.	4. 1 hours to 2 hours.<br>5. 2 hours to 3 hours.<br>6. More than 3 hours.

(7) Do you go to tutoring schools for a fee, or do you have paid tutors? If yes, how many days do you go to or do paid tutors come to your place? (circle the number applies.)

1. No.<br>2. A day a week.<br>3. Two days a week.	4. Three days a week.<br>5. Four days a week.<br>6. Five days a week.	7. Six days a week.<br>8. Every day.

(8) Are you participating the after-school class in school*? Choose the number closest to your condition.

* The educational support conducted after school or during long vacation in school.

1. Participating always	2. Sometimes participating	3. Rarely participate	4. Have never participated

(a) To who answered "3. Rarely participate" and "4. Have never participated" in (8).

What is the reason you do not participate so often?

1. It is not held in school.	5. There is no need to participate.
2. I have to do house work.	6. I did not know about it.
3. I have no time due to extracurricular activities.	7. Others（Be specific： ）
4. I am not interested in it.	

(9) If you participate in after-school class in school, what do you want to do?

1. I want to ask what I could not understand at class.	4. Others（Be specific： ）
2. I want to ask where I could not understand about homework (including homework during summer vacation).	5. I do not know.
3. I want to listen to what I am interested in or something difficult.	

(10) When did you do your summer homework this year?

(circle the number applies.)

1. Did all the homework at the beginning of summer vacation.	3. Did it little by little almost every day.
2. If anything, did it at the beginning of summer vacation.	4. If anything, did it at the end of summer vacation.
	5. Did all the homework at the end of summer vacation.

Q 10. Please tell us about your communy.

(1) Have you ever joined the following events in your community? (circle the number applies.)

1. Summer festival in neighborhood
2. Events such as a sport festival or Christmas party held by neighborhood association
3. Clearning up parks and roads, evacuating training in the community
4. Courses and classes held by child centers and community centers
5. None of 1-4 events

(2) Are there any adults around you, other than your parents, who are like the following? (circle the numbers applies.)

1. Whom you can trust	5. Who take care of you
2. Whom you can consult with no difficulties	6. Who say hello to you when you meet
3. Whom you look up	7. There is no one like 1-6.
4. Whom you would like to be like.	

Q11. Tell us about yourself.

(1) Do you think you are happy? When we set “very happy” as 10-point and “very unhappy” as 0-point, what score do you give? (circle the number applies.)

Very unhappy	Very unhappy
0---1---2---3---4----5---6---7---8---9---10	

(2) Please choose one number which is closet to your usual condition.
(circle the number applies to each.)

	No	Somewhat no	Somewhat yes	Yes
(a) Do you have confidence in yourself?	1	2	3	4
(b) Do you think you can do most things better than others?	1	2	3	4
(c) Do you think you have a lot to be proud of?	1	2	3	4
(d) Do you feel nothing you do seems to work?	1	2	3	4
(e) Are you satisfied with yourself?	1	2	3	4
(f) Do you think you will become a great person?	1	2	3	4
(g) Do you think you are not a very useful person?	1	2	3	4
(h) Are you confident in your opinion?	1	2	3	4
(i) Do you think you do not have much to offer yourself?	1	2	3	4

(3) Have you ever experienced the following situations? (circle the number applies.)

		A lot	Sometimes	Rarely	Never	I do not know
(a)	I did not want to go to school.	1	2	3	4	5
(b)	I was absent from school (due not to illness).	1	2	3	4	5
(c)	I was bullied.	1	2	3	4	5

(4) We do not have happy days all the time; there are days when we feel a little longely and days when we are not happy. How you have felt in the past week? There is no good or bad answer. Please answer as honestly as you can. (circle one of the numbers applies to each.)

		Mostly	Sometimes	Never
(a)	I look forward to thing as much as I used to.	1	2	3
(b)	I sleep very well.	1	2	3
(c)	I feel like crying.	1	2	3
(d)	I like to go out to play.	1	2	3
(e)	I feel like running away.	1	2	3
(f)	I get tummy aches.	1	2	3
(g)	I have lots of energy.	1	2	3
(h)	I enjoy my food.	1	2	3
(i)	I can stick up for myself.	1	2	3
(j)	I enjoy the things I do as much as I used to.	1	2	3

(k)	I have bad dreams.	1	2	3
(l)	I feel very lonely.	1	2	3
(m)	I am easily cheered up.	1	2	3
(n)	I feel so sad I can hardly stand it.	1	2	3
(o)	I feel very bored.	1	2	3

Q12. Tell us about your worries.

(1) Do you have any worries or concerns currenty? (circle the number applies.)

1. Yes.	2. No.

(a) To who answered "1. Yes" in (1).

Whom do you consult with? (circle all the numbers apply.)

1. Mother	11. Health teacher at school
2. Father	12. School social workers
3. Siblings	13. School counsellors
4. Grandparents	14. Counsellors (except for 13) including phone call
5. Other family members or relatives	15. Staffs at neighborhood center, community center or child center
6. Friends	16. Neighbors
7. Seniors or juniors	17. Internet consultation sites
8. School teachers	18. Others（Be specific：　　　　　　）
9. School teachers at extracurricular activities	19. Not consult with any one
10. Teachers at cram school	

(2) Tell us how do you think or behave to your stressful events. Please choose the number closest to your current way of thinking or behaving currently.

(circle the number applies to each.)

	Not at all	Sometimes	Often	Always
a) Trying to change the current situation.	1	2	3	4
b) Cheer up by yourself.	1	2	3	4
c) Trying to see the positive side of things.	1	2	3	4
d) Leaving it as it is.	1	2	3	4
e) Asking others' help to solve the problems.	1	2	3	4
f) Thinking problems as not a big deal.	1	2	3	4
g) Trying to find out the cause of the problems.	1	2	3	4
h) Trying to explain the situation you are in.	1	2	3	4
i) Trying to assume the current experience beneficial.	1	2	3	4

This is the end of the question. Thank you for answering the questions.

One last time, make sure that there are no unfilled items and put it in a brown envelope, seal it, and then along with the white envelope of your gardians, please submit it to the school in a large envelope.

Questionnaire

Kochi Child Health Impact of Living Difficulty (K-CHILD) study

To gardians of second-year high school students

- This questionnaire is for the parents of the children to whom the questionnaire was distributed
- Survey is anonymous and contains any questions on the personal information that identifies the individual. All the answers will be statistically processed. Therefore, other people will not be able to find out who wrote what.
- Please fold the completed questionnaire into three, put into a white envelope, and seal it. Together with the child's brown envelope sealed in the same way, put them in a large envelope that was enclosed in the package when it was distributed. Close it up tightly with glue or tape, then submit it to school.
- The school or homeroom teacher will not open the envelope. Only those companies commissioned by the Kochi Children's Family Division at the Kochi Prefectural Office unseal the envelope.
- There are some personal inquiries about yourself or your family. Although you do not have to answer questions that is difficult to answer or questions that you do not want to answer, please tell us your answers as they are since the privacy is protected.
- The term "child" in this questionnaire signifies the child to whom a questionnaire has been distributed. Even if the child has siblings, it refers only those children to whom a questionnaire has been distributed.

-If you have any questions, please call us. -
Division of Children and Family Affairs of the Kochi Prefectural Government's Regional Welfare Department
Phone number: 088-823-9655
(Phone reception desk is open: Monday-Friday: 8:30 - 17:15)

- ❖ Excluding December 29th to January 3rd and holidays.

Q1. What is your relationship with the child. Please answer the relationship from the child's point of view.

1. Mother	5. Father's mother	9. Staffs of institution
2. Father	6. Father's father	（→To staff of institution, please answer the following questions as much as you can.）
3. Mater's mother	7. Siblings	
4. Mater's father	8. Others（Be specific：　　　　）	

Q2. Tell us the current marital status of the child's parents.

1. Married	3. Bereaved
2. Divorced	4. Unmarried

Q3. Who are living with the child with? Please tell us each number and the total number. Please include those who are living outside of home due to business.

1. Mother	5. Father's mother	9. Younger brother （　　people）
2. Father	6. Father's father	10. Younger sister （　　people）
3. Mother's mother	7. Older brother （　　people）	11. Other relatives （　　people）
4. Mother's father	8. Older sister （　　people）	12. Others （　　people）

→（　　　　　）people in total * please write the number including you and the child

Q4. Tell us about your residence.

(1) Where are you living currently?

1. Outside of Kochi Prefecture　　2. Inside of Kochi Prefecture; the name of cities（　　　　　　）

(2) How many times have you moved after the child were born?

(a) Before entering the elementary school	（　　　）times
(b) During the elementary school	（　　　）times
(c) During the junior high school	（　　　）times
(d) After entering the high school	（　　　）times

Q5. Please tell us all the institutions your child has attended before entering elementary school.

1. Certified nursery schools	4. Kindergarten
2. Uncertified nursery schools	5. There is no institution
3. Certified child care center	

Q6. Tell us about your future education.

(1) Untile which level of education are you willing to let your child to get?

1. To high school	6. To university (in Kochi)
2. To vocational school * (in Kochi)	7. To university (out of Kochi)
3. To vocational school * (out of Kochi)	8. To graduate university (in Kochi)
4. To technical college or university (in Kochi)	9. To graduate university (out of Kochi)
5. To technical college or university (out of Kochi)	10. Others（Be specific： ）

*This school is the one you are going to after graduating high school.

(2) Until which level of education do you think you will let your children to get in reality?

1. To high school	6. To university (in Kochi)
2. To vocational school * (in Kochi)	7. To university (out of Kochi)
3. To vocational school * (out of Kochi)	8. To graduate university (in Kochi)
4. To technical college or university (in Kochi)	9. To graduate university (out of Kochi)
5. To technical college or university (out of Kochi)	10. Others（Be specific： ）

*This school is the one you are going to after graduating high school.

(3) Why is your reason of your anwer in (2)?

1. That is what my child wants to.
2. I think most people would go to that level of school.
3. I think that level is enough.
4. Considering my child's academic ability.
5. Lack of financial resources at home.
6. Others（Be specific： ）
7. There is no specific reason.

(a) To who answered "4. Lack of financial resources at home" in (3). In which situation, can you consider you could let your children to go to the school of your child's choice?

1. Only if we get scholarship that you do not need to repay.
2. Only if we get any interest-free scholarship.
3. Only if we get any scholarship.
4. I cannot consider to continue educated since we need to work for earning living costs.
5. Others（Be specific： ）

Q7. Which describes your child's health condition the most?

1. Good	2. Somewhat good	3. Normal	4. Not very good	5. Not good

Q8. Tell us about your child's teeth condition.

Does your child have any cavities currently/ have your child ever had any cavities in the past?

1. Yes →（ teeth）	2. Yes（treatment is completed） →（ teeth）	3. No	4. I do not know

(a) To who answered "1. Yes" in Q8.

Does your child currently take any treatment?

1. Yes	2. No	3. I do not know

(b) To who answered "2. No" in (a).

What is the reason your child does not take any treatment although he/she has cavities.

1. Cannot pay treatment fees.
2. Since the child refused to go.
3. The dentist is located too far from home to go.
4. I am too busy to take the child to dentist.
5. Others（Be specific： ）

Q9. Have you ever thought your child should be seen by doctors but did not actually have your child seen within the last one year?

1. Yes	2. No

(a) To who answered "1. Yes" in Q9. What is the reason of that? Please choose the answer most closest to your situation.

1. I could not afford payments since I did not have public medical insurance.
2. I do not think I could afford the co-payments at hospital although I have joined public medical insurance.
3. Your child did not want to go to hospital.
4. The hospital is located too far to go to.
5. I was too busy to bring my child to hospital.
6. I thought I should let my child seen by doctors but on second thought I could not find any necessities.
7. My health condition was too but to bring my child to hospital.
8. Others（Be specific： ）

Q10. Tell us about your child's condition on vaccination.

*** Regular vaccination (Tubeculosis (BCG), Measles, Rubella, Polio, etc.) is free of charge. Optional vaccination (Flu, Mumps, etc.) is for fees.**

	Vaccinated	Not vaccinated	I do not know
(1) Regular vaccination	1	2	3
(2) Optional vaccination (Flu) * the past one year	1	2	3
(3) Optional vaccination (Mumps)	1	2	3

(a) To who answered "2. Not vaccinated" in Q10 about regular vaccination.
What is the reason you do not let your child take?

1. I did not know it is for free.
2. My child did not want to be vaccinated.
3. The hospital is located too far to go to.
4. I was too busy to bring my child to hospital.
5. I thought I did not need to let my child vaccinated considering the child's condition.
6. I did not know where and when the child should be vaccinated.
7. My child had a fever at the timing of vaccination.
8. My health condition was too bad to bring child to hospital.
9. Others（Be specific：　　　　　　　　　　　　　　　　　　　　　　）

Q11. Please choose the number closest to the conditions to each of the following sections regarding your child's behavior during the past 6 months.

Please answer all the following questions even if you are unsure.

	True	Somewhat true	Certianly true
(1) Considerate of other people's feelings.	1	2	3
(2) Restless, everactive, cannot stay still for long.	1	2	3
(3) Often complains of headaches, stomachaches or sickness.	1	2	3
(4) Shares readily with other children (treats, toys, pencils etc.)	1	2	3
(5) Often has temper tantrums or hot tempers.	1	2	3
(6) Rather solitary, tends to play alone.	1	2	3
(7) Generally obedient, usually does what dults request	1	2	3
(8) Many worries, often seems worried.	1	2	3
(9) Helpful if somone is hurt, upset or feeling ill.	1	2	3
(10) Constantly fidgeting or squirming.	1	2	3
(11) Has at least one good friend.	1	2	3
(12) Often fights with other children or bullies them.	1	2	3
(13) Often unhappy, down-hearted or tearful.	1	2	3
(14) Generally liked by other children.	1	2	3
(15) Easily distracted, concentration wanders.	1	2	3
(16) Nervous or clingy in new situations, easily loses confindence.	1	2	3
(17) Kind to younger children.	1	2	3
(18) Often lies or cheats.	1	2	3
(19) Picked on or bullied by other children.	1	2	3
(20) Often volunteers to help others (parents, teachers, other children).	1	2	3
(21) Thinks things out before acting.	1	2	3
(22) Steals from home, school or elsewhere.	1	2	3
(23) Gets on better with adults than with other children.	1	2	3
(24) Many fears, easily scared.	1	2	3
(25) Sees tasks through to the end, good attention span.	1	2	3

Q12. Tell us about health condition of the child's mother and father.

*** Please write answer separately for both parents in your response form. If the parent is absent, please leave your response blank.**

(1) Tell us about the ages, heights, and weights of the child's mother and father.

* The response to this question will be used to find the correlations with physique of the child. Please respond as accurately as possible.

Mother	
Age	( ) years old
Height	( ) cm
Wight	( ) kg

Father	
Age	( ) years old
Height	( ) cm
Wight	( ) kg

(2) Do the child's mother and father smoke habitually. Or, Have they ever smoked?

Mother
1. Currently smoking habitually.
2. Smoked before but is not currenly.
3. Have never smoked before.

Father
1. Currently smoking habitually.
2. Smoked before but is not currenly.
3. Have never smoked before.

To who answered "1. Currently smoking habitually." And "2. Smoked before but is not currently." In (2).

(a) Do they smoke/ have they smoked inside of house?

Mother
1. Yes
2. No

Father
1. Yes
2. No

(b) Do they smoke/ have they smoked in front of the child?

Mother
1. Always
2. Sometimes
3. Never

Father
1. Always
2. Sometimes
3. Never

Q13. Tell us about the occupation and experience of the child's mother and father.

*** Please write answer separately for both parents in your response form. If the parent is absent, please leave your response blank.**

(1) What is the current occupation of the child's mother and father?

Mother	Father
1. Company executives	1. Company executives
2. Full-time employees of a private company	2. Full-time employees of a private company
3. Regular employees such as civil servants	3. Regular employees such as civil servants
4. Contract, temporary, and part-time employees	4. Contract, temporary, and part-time employees
5. Part-time employees	5. Part-time employees
6. Self-employed (including family business)	6. Self-employed (including family business)
7. Free enterprise	7. Free enterprise
8. Staff members	8. Staff members
9. Working in a way other than 1 to 8	9. Working in a way other than 1 to 8
10. Full-time housewife	10. Full-time housewife
11. Student	11. Student
12. Retired	12. Retired
13. Seeking employment	13. Seeking employment
14. Other unemployed	14. Other unemployed

(2) To who answered "1" to "9" in (1).

(a) Are you allowed to return home from work if the child got sick suddenly.

Mother	Father
1. Can go back immediately	1. Can go back immediately
2. Can go back	2. Can go back
3. Cannot go back easily	3. Cannot go back easily
4. Cannot go back ever	4. Cannot go back ever

(b) How many hours does the child's mother and father work a week? * if they work in several workplaces, please sum up all the working time.

Mother	( ) hours a week on average
Father	( ) hours a week on average

(c) Please choose the time the child's mother and father usually arrive home. * if they work at home, please choose the time you finish to work.

Mother	Father
1. Until 6 pm	1. Until 6 pm
2. 6pm to 8pm	2. 6pm to 8pm
3. 8pm to 10pm	3. 8pm to 10pm
4. After 10pm (including early morning)	4. After 10pm (including early morning)
5. There is no fixed time	5. There is no fixed time

(3) Tell us about the last school the child's mother and father went/ is going.
(if they graduated high school at the highest, please choose "3. Graduated high school"; if they dropped out of school, please choose "2. Dropped out of high school".)

Mother	Father
1. Graduated junior high school	1. Graduated junior high school
2. dropped out of high school	2. dropped out of high school
3. Graduated high school	3. Graduated high school
4. Graduated a vocational school (after high school)	4. Graduated a vocational school (after high school)
5. Graduated a junior college/ technical college	5. Graduated a junior college/ technical college
6. Dropped out of university	6. Dropped out of university
7. Graduated university	7. Graduated university
8. Graduated graduate university	8. Graduated graduate university
9. Others（　　　　　　）	9. Others（　　　　　　）
10. I do not know	10. I do not know

Q14. Tell us child's home economics.

(1) How do you feel about the current living conditions?

1. Very affordable	3. Normal	5. In very financial difficulty
2. Somewhat affordable	4. In financial difficulty	

(2) How do you rate of your family's social status within a whole society?

1. High	3. Middle	5. Low
2. Middle-high	4. Middle-low	6. I do not know

(3) Among the following items, is there anything that you do not have due to financial reasons.

1.	A washing machine	8.	A computer connected to the internet
2.	A rick cooker	9.	A bathtub for household
3.	A vaccuum	10.	A bed per person
4.	A hearter/ hearting appliance	11.	A curtain
5.	An air-conditioner	12.	A car
6.	A microwave	13.	A savings (more than 50,000 JPY)
7.	A phone (including both landlines and mobiles)	14.	There is nothing applicable

(4) Among the following items, is there anything that you could not pay or buy due to financial reasons?

	Could not pay/buy	Could pay/buy	Not applicable (no need to pay/buy)
1. School field trips/ extracurricular activities	1	2	3
2. School textbooks	1	2	3
3. Rent	1	2	3
4. Housing loans	1	2	3
5. Electricity bills	1	2	3
6. Gas bills	1	2	3
7. Water bills	1	2	3
8. Phone bills (including both lanllines and moblies)	1	2	3
9. Insurance fees for public pension, national health insurance, and/or public nursing care	1	2	3
10. Bus or train fees for commuting	1	2	3
11. Food	1	2	3
12. Clothes or bags necessary for child	1	2	3

(5) Tell us about the living costs and educational fees for the child whom the questionnaire was distributed to. How much do you spend to the following each item per month? Please answer the average cost.

* If there is no cost, please fill "0" in the parentheses.

	Monthly cost
(a) Tuition/ school fees	Approximately ( ) JPY
(b) Extracurricular educational fees such as cram school	Approximately ( ) JPY
(c) Lesson fees (excluding sport club)	Approximately ( ) JPY
(d) Sport club	Approximately ( ) JPY
(e) Allowances	Approximately ( ) JPY
(f) Mobile phone and smart phone bills	Approximately ( ) JPY

(6) Tell us about the cost you spent when the child entered high school (Admission fees, payment for clothes and bags, shoes and teaching materials). Did you feel it is difficult to pay?

1. Difficult	2. Somewhat difficult	3. Not so much difficult	4. Not difficult

(a) To who answered “1. Difficult” or “2. Somewhat difficult” in (6).

How did you deal with it? Please choose the closest answers.

1. Made ends meet by saving money.	5. Borrowed money from credit cards.
2. Got a few second-hand items.	6. Could not prepare before entering.
3. Borrowed money from friends and relatives.	7. Others（Be specific：　　　　　）
4. Borrowed money from local municipalities.	

(7) Are you receiving scholarship for high school students in your household for the sake of the child?

* Scholarship for high school students is available to household that are exempt from tax on income and aims to support educational fees except for tuition fees.

1. Receiving	2. Not receiving	3. I do not know

(a) To who answered “2. Not receiving” in (7). What is the reason you are not receiving it?

1. Not applied (necessary but not meet the requirement)
2. Not applied (necessary but not know how to apply it)
3. Not applied (necessary but feel resistence to application)
4. Not applied (feel no necessities)
5. Applied but not approved
6. Not knowing the scholarship for high school students
7. Others（Be specific：　　　　　）

(8) How much of the following public pension and social security benefits is your household receiving at any one time?

If there is any corresponding income, please fill "0" in the parentheses.

It includes any pension/ benefits related to siblings.

Child support allowance is the allowance for household whose income is below the threshold and has only one parent.

	Payment at a time
(a) Child support allowance	( ) JPY （Payed once per four months）
(b) Special child support allowance	( ) JPY （Payed once per four months）
(c) Annuities (survivor's pensions, age pensions)	( ) JPY （Payed once per two months）
(d) Public Allowance	( ) JPY

(9) Tell us about the income excluding public pension and social security benefits you described in (8).

How much is the approximate total annual income of all the members who share the livelihood?

* income includes income from work (including part-time jobs), business income (self-employment, etc.), agricultural income, real estate income, interest and dividends and personal pensions. Please exclude the income from public pension and social security you answered in (8).

* If there is several income resources (income from work by father, business income by mother for example), please tell us the approximate total income.

1. There is no income (0 JPY)	5. 2.00 – 2.99M JPY	9. 6.00 – 6.99M JPY
2. 1 - 49,999 JPY	6. 3.00 – 3.99M JPY	10. 7.00 – 7.99M JPY
3. 50,000 – 99,999 JPY	7. 4.00 – 4.99M JPY	11. 8.00 – 8.99M JPY
4. 1M – 1.99M JPY	8. 5.00 – 5.99M JPY	12. More than 9.00M JPY
		13. I do not know

Q15. Tell us the relationship between the child and family members including you.

(1) Do you do the following activities in your family?

	Almost every day	3-4 times a week	1-2 times a week	1-2 times a month	Rarely
a) Helping the child's study	1	2	3	4	5
b) Exercising with the child	1	2	3	4	5
c) Talking about school activities with the child	1	2	3	4	5
d) Talking about social/political/economical problems with the child	1	2	3	4	5
e) Talking about TV (excluding news) with the child	1	2	3	4	5
f) Cooking with the child	1	2	3	4	5
g) Going out with the child	1	2	3	4	5

(2) Do you have opportunities to think or talk about the child's future plan (such as dreams, carrer path, and occupation) with the child in your family?

1. Often do	2. Sometimes do	3. Not so much	4. Have never done before

(3) Do you have high expectations for your child's potential in your family?

1. Have high expectations	3. Have few expectations	5. I do not know
2. Have a few expectations	4. Have no expectations	

(4) How often do you cook your child's meal in your family? (including easy meal like stirred eggs)

1. Almost every day	4. A few days a month (on weekeds/ holidays)
2. 4 to 5 days a week	5. Rarely cook
3. 2 to 3 days a week	

(5) Do you do the following activities to the child in your family?

	Yes, we do	No, we do not	
		Do not want to（Not willing to）	Cannot afford
(a) Celebrating the birth day	1	2	3
(b) Going to family trip once a year	1	2	3
(c) Giving Christmans present/ new year's gift (e.g., money)	1	2	3

(6) Do you do the following reactions to the child in your family?

	Often	Sometimes	Parely	Never
a) Hitting the child's body (e.g., bottom, hands, head, face, etc.)	1	2	3	4
b) Yelling at the child	1	2	3	4
c) Beating the child	1	2	3	4
d) Shutting the child outside	1	2	3	4
e) Neglecting the child	1	2	3	4
f) Not feeding the child	1	2	3	4
g) Insulting the child repeatedly	1	2	3	4
h) Leaving the child alone in the house at night	1	2	3	4
i) Having a big fight in front of the child	1	2	3	4

(7) **<u>To whose child is not living together with grandparnts.</u>**

(a) How many hours does it take to go to grandparents' house? Please tell us how to go usually and how many hours to take.

① To the mother's parents' house

1. on foot	2. By bike	3. By car	4. By bus	5. By train	6. By air plane	（ ）Hours	（ ）Minutes

② To the father's parents' house

1. on foot	2. By bike	3. By car	4. By bus	5. By train	6. By air plane	（ ）Hours	（ ）Minutes

(b) How much help of child rearing do you receive from the child's grandparents?

Parents of the mother	Parents of the father
1. On a daily basis	1. On a daily basis
2. Frequently	2. Frequently
3. Sometimes	3. Sometimes
4. Rarely	4. Rarely
5. I do not know	5. I do not know

Q16. Tell us about you, who are answering this questionnaire.

(1) Do you think you are happy? When we set "very happy" as 10-point and "very unhappy" as 0-point, what score do you give?

Very unhappy	Very unhappy
0---1---2---3---4----5---6---7---8---9---10	

(2) How long have you lived in the current community?

( ) years ( ) months

(3) Do you socialize with your neighbors in your family?

1. Having a very close relationship.	3. Socializing but do not have close relationship.
2. Having a close relationship.	4. Almost no/ no relationship.

(4) How often do you receive food from or share food with people in your community?

Receving food	Sharing food
1. On a daily basis	1. On a daily basis
2. Frequently	2. Frequently
3. Sometimes	3. Sometimes
4. Rarely	4. Rarely
5. Never	5. Never

(5) Are you belonging to some groups, sport club, or private organizations such as NPO, and neighborhood associations, and doing any activities?

1. Yes	2. No

(6) Do you have any person you can consult with if you are in trouble or having any worries?

1. No	2. 1 to 2 people	3. 3 to 4 people	4. 5 to 7 people	5. More than 7 people

(a) To who answered any numbers except for “1. No” in (6).

Who/where is that?

1. Partners	5. Friends living nearby
2. Own parents	6. Friends living far
3. Partners’ parents	7. Staff at work places
4. Siblings/ other relatives	8. Others（Be specific：　　　　）

(7) Have you ever consulted with the following public institutions? If not, please choose the most suitable resons.

	Yes, I have consulted.	No, I have never consulted.			
		Have never feel like to consult with.	Want to consult but felt resistant.	Time and place was not suitable.	I did not know how to consult.
1. Municipal offices (including welfare offices, health center, etc.)	1	2	3	4	5
2. Child and family support center	1	2	3	4	5
3. Teachers in schools, nursery schoos and kindergartens	1	2	3	4	5
4. School cousellors	1	2	3	4	5
5. School social workers	1	2	3	4	5
6. Demogracy and childre's committee members	1	2	3	4	5
7. The county welfare and health department	1	2	3	4	5
8. Child guidance centers	1	2	3	4	5
9. Police	1	2	3	4	5
10. Labour information centers	1	2	3	4	5
11. Consultation sites on the internet	1	2	3	4	5
12. Other public institutions	1	2	3	4	5

(8) How do you feel about your health condition?

1. Good	2. Somewhat good	3. Normal	4. Not very good	5. Not good

(9) During the past thirty days, about how often did you feel…

	All of the time	Most of the time	Some of the time	A little fo the time	None of the time
a) … nervous?	1	2	3	4	5
b) … hopeless?	1	2	3	4	5
c) … restless or fidgety?	1	2	3	4	5
d) … so depressed that nothing could cheer you up?	1	2	3	4	5
e) … that everything was an effort?	1	2	3	4	5
f) … worthless?	1	2	3	4	5

(10) Have you ever experienced the following events during childhood?

1. My mother died.	5. My parnets played violence to me.
2. My father died.	6. I was neglected (diets, clothes).
3. My parents divorced.	7. My parents said something hurt me.
4. My father played violence to my mother.	8. We experienced financial hardships.

(11) How do you rate of your family’s social status within a whole society <u>in your childhood</u>?

1. High	3. Middle	5. Low
2. Middle-high	4. Middle-low	6. I do not know

Q17. Tell us about how you collect the information and the conditions that you utilize the support.

(1) Where do you get the information on the child’s education, health and medicine?

Please choose the main three sources.

1. Partner	10. SNS (facebook, Twitter, LINE, etc.)
2. My family or relatives	11. Internet or blog
3. My friends and neighbors	12. Municipal publicity
4. Teachers in school	13. News papers
5. Teachers in club activities	14. Books or magazines
6. Municipal survice office	15. Others（Be specific： ）
7. Health center	16. There is no sources.
8. Hospital	
9. Television/ radio	

(2) Have you ever received any support for the child, whom the questionnaire was distributed, from the following resources?

If not, please choose the most suitable reasons.

	I have ever used.	I have never used.					
		I did not feel like to be supported.	I want to use but did not know how to use.	Time or other system was not usable.	I do not know how to use it.	I do not know about the system.	There is no system available.
a) A place where you can talk about anything on the child (outside of school)	1	2	3	4	5	6	7
b) Children's cafeteria	1	2	3	4	5	6	7
c) Food assistance by food bank	1	2	3	4	5	6	7
d) A place where children in junior high school and above can spend their free time	1	2	3	4	5	6	7
e) Educational support from school	1	2	3	4	5	6	7
f) Educational support outside of school	1	2	3	4	5	6	7

＜references＞

(b) Children's cafeteria	A place where private organizations provide free or discounted meals to children for the purpose of supporting their food.
(c) food bank	Activities in which NPOs and other organizations retrieve food from food manufacturers and provide it to welfare facilities and people in need of support, free of charge, for food that would otherwise be disposed of when it is still edible.

(a) Are you currently interested in using the following support system?

1. A place where you can talk about anything on the child (outside of school)
2. Children's cafeteria
3. Food assistance from food bank
4. A place where children in junior high school and above can spend their free time
5. Educational support from school
6. Educational support outside of school

(3) Have you ever used the following support system in your family? If not, please choose the most suitable rasons.

	I have used.	I have never used.				
		I have never felt like using the system (at the first place, I was not eligible to the system.)	I would like to use but did not know how to use it.	I would like to use but it was difficult to use.	I did not know how to use it.	I did not know anyting about the system.
a) Public welfare funds	1	2	3	4	5	6
b) Public Assistance	1	2	3	4	5	6
c) Mother/ father and child welfare fund	1	2	3	4	5	6
d) Child support allowance	1	2	3	4	5	6

＜References＞

a) Public welfare funds	Low-interest or interst-free loans for households with income below a certain level. You can contact with your local social welfare council.
b) Public Assisntace	If you have no other way to pay for living expenses or medical care due to illness or unemployment, etc. and have no other choice, you may be able to get a living allowance under certain conditions. This system provides assistance in eight areas: education, housing, medical care, nursing care, childbirth, occupation and funerals. Please contact your local government office for assistance.
c) Mother/ father and child welfare fund	A low-interest or interest-free loan for single-parent households. Please contact your local government office.
d) Child support allowance	An allowance to support single-parent households with incomes below a certain level. Municipal offices are open to the public.

This is the end of the question. Thank you for answering the questions.

One last time, make sure that there are no unfilled items and put it in a brown envelope, seal it, and then along with the white envelope of your gardians, please submit it to the school in a large envelope.

Questionnaire

Kochi Child Health Impact of Living Difficulty (K-CHILD) study

To second-year high school students

Purpose of the survey

- This is the questionnaire aiming to find out the dreams, worries and living situations of the children in Kochi Prefecture.

A request to you

- Please complete this servey on your own. There is no need for your family to see your answer.
- Please do not write down your name.
- You do not need to questions that you would not like to answer. Also, there are no wrong or correct answers. Please write your thoughts on this question.
- When you are asked about "parents" in the question, please respond as a parent or a guardian of you.

How to submit your questionnaire

- When you have finished answering, fold it into three and put it in a brown envelop by yourself. Then close it with glue or tape.
- Put your envelope and your guardian's envelope in a large envelope together.
- Close the large envelope tightly with glue or tape, and take it to school.

-If you have any questions, please call us. -

Division of Children and Family Affairs of the Kochi Prefectural Government's Regional Welfare Department

Phone number: 088-823-9655

(Phone reception desk is open: Monday-Friday: 8:30 - 17:15)

- ❖ Excluding December 29th to January 3rd and holidays.

Q1. Please tell us about your gender and date of birth.

(1) What is your gender? (circle one of them.)

1. Male	2. Female

(2) When is your birth day?
(please answer with a number in parentheses.)

Year (   ) Month (   )

Q2. Please tell us your height and weight. (please answer with a number in parentheses.)

(1) Height	（　　　　　） cm
(2) Weight	（　　　　　） kg
(3) Month of measurement	Year （　　　　） Month （　　）

Q3. Do you have any of the following items? Please choose the one that is closest to your situation. (please circle one of the numbers that apply to each.)

	I have.	I want to have but do not.	I do not want.
(1) New clothes (not someone's used clothes)	1	2	3
(2) At least two pairs of right sized shoes	1	2	3
(3) A jacket and coat for winter	1	2	3
(4) Beds for your own use	1	2	3
(5) A place where you can study at home	1	2	3
(6) A computer connected to the internet	1	2	3
(7) An electronic dictionary	1	2	3
(8) Your own room	1	2	3
(9) About 5,000 JPY per month that you can spend freely	1	2	3
(10) A cell phone, a smart phone	1	2	3
(11) A money to go out with friends	1	2	3
(12) A money you invest in yourself （self-help books, vocational training courses, etc.）	1	2	3

Q4. Do you have an occupation that you want to be in the future? (Circle the number applies the most.）

1. Yes	2. No → To (c)

To who answered "yes" in Q4.

a) What is the occupation that you want to be? Please write down in the box below.

b) What are the reasons you want to be? (circle every number apply.)

1. You can earn a lot of money.	5. It is useful and contributes to society and people.
2. You can get position and prestige comes with it.	6. There's no threat of losing one's jobs.
3. I can make use of my abilities and aptitudes.	7. I want to take over my parents' business.
4. It suits my interests and preferences.	8. Other（Be specific： ）

<u>To who answered "no" in Q4.</u>

c) What is the reason you do not have any occupation that you want to be? (circle one of the numbers applies.)

1. I cannot think of anything specific.	4. I do not think I am able to be what I want to be.
2. I will take any job that generates an income.	5. I do not know.
3. I do not want to work.	6. Other（Be specific： ）

Q5. Please tell us about your higher education.

(1) Which level of education do you want to get in the future? (circle one of the numbers applies)

1. To high school	6. To university (in Kochi)
2. To vocational school * (in Kochi)	7. To university (out of Kochi)
3. To vocational school * (out of Kochi)	8. To graduate university (in Kochi)
4. To technical college or university (in Kochi)	9. To graduate university (out of Kochi)
5. To technical college or university (out of Kochi)	10. Others（Be specific： ）

*This school is the one you are going to after graduating high school.

(2) Which level of education do you think you will get in the future? (circle one of the numbers applies.)

1. To high school	6. To university (in Kochi)
2. To vocational school * (in Kochi)	7. To university (out of Kochi)
3. To vocational school * (out of Kochi)	8. To graduate university (in Kochi)
4. To technical college or university (in Kochi)	9. To graduate university (out of Kochi)
5. To technical college or university (out of Kochi)	10. Others（Be specific： ）

*This school is the one you are going to after graduating high school.

(3) Why is your reason of your anwer in (2)? (circle every number apply.)

1. That is what I want to.
2. I think most people would go to that level of school.
3. Considering my academic ability.
4. Lack of financial resources at home.
5. That is what my parents want to. (excluding the financial problems.)
6. Others（Be specific： ）
7. There is no specific reason.

(a) To who answered “4. Lack of financial resources at home” in (3). In which situation, can you consider going to the school of your choice? (circle one of the numbers applies.)

1. Only if I get scholarship that you do not need to repay.
2. Only if I get any interest-free scholarship.
3. Only if I get any scholarship.
4. I cannot consider to continue educated since I need to work for earning living costs.
5. Others（Be specific： ）

Q6. Please tell us about your friends and family

(1) How many friends like the following do you have in total? If you do not have, please answer “0”. (please answer with a number in parentheses.)

a) A good friend in a same school （ ）people

b) A good friend in a different school （ ）people

c) A friend you can consult with if you have any worries （ ）people

(2) How much influence do you think does your opinions and behaviors have on your classmates? (circle one of the numbers apply.)

1. Very much	2. To some extent	3. Not very much	4. Not at all

(3) Please tell us about your relationship with your parents. Please choose every answer apply. (circle the numbers apply)

1. Talking about school events and my friends.	4. Helping me learn.
2. Talking about my future and carrer.	5. Listening to me.
3. Talking about social events and news.	

Q7. Please tell us about your daily life.

(1) **With whom** do you usually spend time after school **during weekdays.** (circle one of the numbers applies.)

1. Your family (including grandparents and relatives)
2. Adults except for your family (neighbors, teachers at a cram school, coaches at a sport club, etc.)
3. Friends of my school
4. Fiends of the different school (friends you met at a local sport club, neighbors, friends from the same elementary and junior high school)
5. Coworkers such as part-time jobs
6. Spending alone

(2) Where do you spend your time after school on weekdays and for how many days a week? (circle the number applies to each.)

		Every day	3 to 4 days a week	1 to 2 days a week	Never
(a)	Home	1	2	3	4
(b)	Grandparents' or relative's house	1	2	3	4
(c)	Friend's house	1	2	3	4
(d)	Extracarricular activities or cram school	1	2	3	4
(e)	Extracarricular activities at school	1	2	3	4
(f)	Sport club（base ball stadium, soccer stadium, etc.）	1	2	3	4
(g)	Work places for part-time jobs	1	2	3	4
(h)	Park	1	2	3	4
(i)	Library	1	2	3	4
(j)	Restaurant, shopping street or supermarket	1	2	3	4
(k)	Game center	1	2	3	4
(l)	Others（Be specific：　　　　　　　　　）	1	2	3	4

(3) Which place are you relaxed the most from (a) to (i) in (2)? Please choose the one and write down the alphabet in the parenthesis. If you do not have place you are relaxed, please circle "no, there is no place I feel relaxed."

Alphabet（　　　　　　）	No, there is no place I feel relaxed.

(4) How long do you spend time for each activity? (circle the number applies to each.)

		More than 2 hours a day	1 to 2 hours a day	Less than 1 hour a day	4 to 5 days a week	2 to 3 days a week	One day a week	Merely/ never
(a)	Playing with a computer game	1	2	3	4	5	6	7
(b)	Watching TV or DVDs	1	2	3	4	5	6	7
(c)	Using a cell phone, tablet or smart phone	1	2	3	4	5	6	7
(d)	Reading a book	1	2	3	4	5	6	7
(e)	Indoor activity except for (a) to (d)	1	2	3	4	5	6	7
(f)	Houseworks (laundry, clearning, cooking, tyding up, etc.) or supporting your siblings.	1	2	3	4	5	6	7
(g)	Exercise or physical activities	1	2	3	4	5	6	7
(h)	Part-time job	1	2	3	4	5	6	7

(5) How many books did you read within the last one month? (circle the number applies.)

※Please count the books you read outside of home. Also, count the electronic books that you can read with a laptop, tablet or smartphone. Please exclude any journals or comics regardless of their contents.

1. Did not read<br>2. 1 book	3. 2 to 3 books<br>4. 4 to 7 books	5. 8 to 11 books<br>6. More than 11 books

(6) How long do you sleep on weekdays? (circle the number applies.)

1. More than 10 hours<br>2. 9 to 10 hours	3. 8 to 9 hours<br>4. 7 to 8 hours	5. 6 to 7 hours<br>6. Less than 6 hours

(7) What time do you wake up and go to sleep? (circle the number applies to each.)

Time you wake up	Weekdays	Weekends
Earlier than 6 am	1	1
6 am to 7 am	2	2
7 am to 8 am	3	3
8 am to 9 am	4	4
9 am to 10 am	5	5
Later than 10 am	6	6

Time you go to sleep	Days except for Fri. and Sat.	Fridays and Saturdays
Ealier than 8 pm	1	1
8 pm to 9 pm	2	2
9 pm to 10 pm	3	3
10 pm to 11 pm	4	4
11 pm to 12 pm	5	5
Later than 12 pm	6	6

Q8. Please tell us about your diet and health.

(1) Do you usually eat breakfast everyday on weekdays? (circle the number applies.)

1. Every day (5 days a week)<br>2. Most days (3 to 4 days a week)	3. Few days (1 to 2 days a week)<br>4. Not eat

a) To who answered “2. Most days”, “3. Few days”, “4. Not eat” in (1). Which of the reasons is closest to your reason why you do not eat breakfast? (circle the number applies.)

1. There is no time to eat.<br>2. The meal is not ready.<br>3. There is no appetite in the morning.	4. There is no specific reason.<br>5. Others（Be specific： ）

(2) With whom do you eat breakfast on weekdays? (circle all the numbers apply.)

1. Family members (parents)<br>2. Family members (siblings)<br>3. Other family members (grandparents, etc.)	4. Eating alone<br>5. Do not eat breakfast<br>6. Others（Be specific： ）

(3) With whom do you eat supper on weekdays? (circle all the numbers apply.)

1. Family members (parents)	4. Eating alone
2. Family members (siblings)	5. Do not eat breakfast
3. Other family members (grandparents, etc.)	6. Others (Be specific：)

(4) How much do you usually eat/ drink the following foods/ drinks except for school meal?
(circle the number applies to each.)

		Evey day	4 to 5 days a week	2 to 3 days a week	Less than one day a week	Do not eat/ drink
(a)	Vegetable	1	2	3	4	5
(b)	Fruit	1	2	3	4	5
(c)	Meat or fish	1	2	3	4	5
(d)	Cup and instant noodles	1	2	3	4	5
(e)	Rice balls and bento boxes bought at convenience stores	1	2	3	4	5
(f)	Junk food	1	2	3	4	5
(g)	Snacks	1	2	3	4	5
(h)	Juices	1	2	3	4	5

(5) What do you eat first at a meal? (circle the number applies.)

1. Rice or bread (staple foods)	3. Vegetable	5. Do not decide
2. Meal or fish	4. Soup	6. Do not know

(6) Are you currently on a diet? (circle the number applies.)

1. Yes.	2. Not currently but was on a diet in the past.	3. No.

(ア) To who answered “1. Yes”, “2. Not currently but was on a diet in the past” in (6). What are you doing/ have you done to reduce your weight? (circle all the numbers apply.)

1. Reducing the amount of meal.	6. Calculating calory intakes.
2. Cutting back on snacks and evening meals.	7. Doing exercise.
3. Skipping a meal.	8. Taking a medicine.
4. Eating only certain foods (e.g., vegetables, tofu).	9. Spitting out what you eat.
5. Not eating certain foods (e.g., carbohydrate such as rice or bread).	10. Others（Be specific： ）

(7) Do you cook by yourself or help cooking at home? (circle the number applies.)

1. Almost every day (more than 3 days a week)	2. Often (1 to 2 days a week)
3. Sometimes (1 to 3 days a month)	4. Never

(8) How do you feel about your health condition? (circle the number applies.)

1. Good	2. Somewhat good	3. Normal	4. Not very good	5. Not good

(9) How many times do you brush your teeth a day? (circle the number applies.)

1. More than two times a day	2. One time a day	3. Not brushing every day

(10) How long do you usually brush your teeth a time? (circle the number applies.)

1. Less than 1 minute	2. 1 to 2 minutes	3. 3 to 5 minutes	4. More than 5 minutes

(11) Approximately how many cavities do you have right now? Please include the cavities currently under treatment. * please write down “0” if you do not have any cavity. Please do no include the cavities complete the treatment.

（ ）teeth

(a) To who answered any number except for “0” in (11) (who have any cavities curreny).

Are you currently under treatment of cavities? (circle the number applies.)

1. Yes	2. No	3. I do not know

(b) To who answered “2. No” in (a).

What is the reason? (circle the number applies.)

1. Because treatment at dentist is painful.
2. Because treatment at dentist is scary.
3. Because there is no time to go to dentist.
4. Because there is no dentist nearby.
5. Because cavities do not hurt.
6. Because I have never been to dentist.
7. Because parents do not bring me to dentist.
8. Other reasons（Be specific： ）

Q9. Please tell us about your school and study.

(1) Which school do you go to? (circle the number applies.)

1. Full-time	2. Part-time	3. Multi-system/credit (daytime)	4. Multi-system/ credit (nighttime)

(2) Which course are you belong to? (circle the number applies.)

1. General course	3. Agriculture course	5. General studies course
2. Industrial course	4. Commercial course	6. Others（Be specific： ）

(3) Which statement is closest to the situation about you and your school? (circle one the numbers apllies.)

	Do not agree at all.	Not agree so much.	Neigher yes or no.	Agree to some extent.	Agree so much.
(a) I like the atmosphere at class.	1	2	3	4	5
(b) I like my school teacher.	1	2	3	4	5
(c) I enjoy school life.	1	2	3	4	5
(d) I say hello to school teachers and classmates.	1	2	3	4	5
(e) I trust my teacher.	1	2	3	4	5
(f) I trust my classmates.	1	2	3	4	5
(g) I actively participate in the school events.	1	2	3	4	5
(h) I like to meet my teacher.	1	2	3	4	5
(i) I like to meet my classmates.	1	2	3	4	5

(4) Do you understand your school lessons well? (circle one of the numbers apllies.)

1. Understand all the lessons.	3. Do not understand much.	5. Not understand almost all the lessons.
2. Understand most of the lessons.	4. Not understand most of the lessons.	

(5) Whom do you ask to help when you have questions about your study? (circle the number applies.)

1. Your parents	5. Teachers at cram school
2. Other family members (siblings, grandparents, etc.)	6. Other adults
3. School teachers	7. Friends of yours
4. School teachers at extracurricular activities	8. There is no one to ask

(6) How high do you think your grades are in your class? (circle the number applies.)

1. High	3. Middle	5. Low
2. Middle-high	4. Middle-low	6. I do not know

(7) What subject are you good at? (circle all the numbers apply.)

1. Math	5. English	9. Home economics
2. Language	6. P.E.	10. Vocational training
3. Science	7. Art	11.None is applicable.
4. Society	8. Information	

(8) How long do you study except for class lessons. Please tell us your study hours a day. (circle the number applies.) * please include the time you spend at cram school.

1. Not at all.	4. 1 hours to 2 hours.
2. Less than 30 minutes.	5. 2 hours to 3 hours.
3. 30 minutes to 1 hours.	6. More than 3 hours.

(9) Do you go to tutoring schools for a fee, or do you have paid tutors? If yes, how many days do you go to or do paid tutors come to your place? (circle the number applies.)

1. No.<br>2. A day a week.<br>3. Two days a week.	4. Three days a week.<br>5. Four days a week.<br>6. Five days a week.	7. Six days a week.<br>8. Every day.

(10) When did you do your summer homework this year?
(circle the number applies.)

1. Did all the homework at the beginning of summer vacation.<br>2. If anything, did it at the beginning of summer vacation.	3. Did it little by little almost every day.<br>4. If anything, did it at the end of summer vacation.<br>5. Did all the homework at the end of summer vacation.

Q 10. Please tell us about your communy.

(1) Have you ever joined the following events in your community? (circle the number applies.)

1. Summer festival in neighborhood<br>2. Events such as a sport festival or Christmas party held by neighborhood association<br>3. Clearning up parks and roads, evacuating training in the community<br>4. Courses and classes held by child centers and community centers<br>5. None of 1-4 events

(2) Are there any adults around you, other than your parents, who are like the following? (circle the numbers applies.)

1. Whom you can trust<br>2. Whom you can consult with no difficulties<br>3. Whom you look up<br>4. Whom you would like to be like.	5. Who take care of you<br>6. Who say hello to you when you meet<br>7. There is no one like 1-6.

Q11. Tell us about yourself.

(1) Do you think you are happy? When we set “very happy” as 10-point and “very unhappy” as 0-point, what score do you give? (circle the number applies.)

Very unhappy	Very unhappy
0---1---2---3---4----5---6---7---8---9---10	

(2) Please choose one number which is closet to your usual condition.

(circle the number applies to each.)

	No	Somewhat no	Somewhat yes	Yes
(a) Do you have confidence in yourself?	1	2	3	4
(b) Do you think you can do most things better than others?	1	2	3	4
(c) Do you think you have a lot to be proud of?	1	2	3	4
(d) Do you feel nothing you do seems to work?	1	2	3	4
(e) Are you satisfied with yourself?	1	2	3	4
(f) Do you think you will become a great person?	1	2	3	4
(g) Do you think you are not a very useful person?	1	2	3	4
(h) Are you confident in your opinion?	1	2	3	4
(i) Do you think you do not have much to offer yourself?	1	2	3	4

(3) Have you ever experienced the following situations? (circle the number applies.)

		A lot	Sometimes	Rarely	Never	I do not know
(a)	I did not want to go to school.	1	2	3	4	5
(b)	I was absent from school (due not to illness).	1	2	3	4	5
(c)	I was bullied.	1	2	3	4	5

(4) We do not have happy days all the time; there are days when we feel a little longely and days when we are not happy. How you have felt in the past week? There is no good or bad answer. Please answer as honestly as you can. (circle one of the numbers applies to each.)

		Mostly	Sometimes	Never
(a)	I look forward to thing as much as I used to.	1	2	3
(b)	I sleep very well.	1	2	3
(c)	I feel like crying.	1	2	3
(d)	I like to go out to play.	1	2	3
(e)	I feel like running away.	1	2	3
(f)	I get tummy aches.	1	2	3
(g)	I have lots of energy.	1	2	3
(h)	I enjoy my food.	1	2	3
(i)	I can stick up for myself.	1	2	3
(j)	I enjoy the things I do as much as I used to.	1	2	3
(k)	I have bad dreams.	1	2	3
(l)	I feel very lonely.	1	2	3
(m)	I am easily cheered up.	1	2	3
(n)	I feel so sad I can hardly stand it.	1	2	3
(o)	I feel very bored.	1	2	3

Q12. Tell us about your worries.

(1) Do you have any worries or concerns currenty? (circle the number applies.)

1. Yes.	2. No.

(a) To who answered "1. Yes" in (1).

Whom do you consult with? (circle all the numbers apply.)

1. Mother	11. Health teacher at school
2. Father	12. School social workers
3. Siblings	13. School counsellors
4. Grandparents	14. Counsellors (except for 13) including phone call
5. Other family members or relatives	15. Staffs at neighborhood center, community center or child center
6. Friends	16. Neighbors
7. Seniors or juniors	17. Internet consultation sites
8. School teachers	18. Others（Be specific： ）
9. School teachers at extracurricular activities	19. Not consult with any one
10. Teachers at cram school	

(2) Tell us how do you think or behave to your stressful events. Please choose the number closest to your current way of thinking or behaving currently.

(circle the number applies to each.)

	Not at all	Sometimes	Often	Always
a) Trying to change the current situation.	1	2	3	4
b) Cheer up by yourself.	1	2	3	4
c) Trying to see the positive side of things.	1	2	3	4
d) Leaving it as it is.	1	2	3	4
e) Asking others' help to solve the problems.	1	2	3	4
f) Thinking problems as not a big deal.	1	2	3	4
g) Trying to find out the cause of the problems.	1	2	3	4
h) Trying to explain the situation you are in.	1	2	3	4
i) Trying to assume the current experience beneficial.	1	2	3	4

This is the end of the question. Thank you for answering the questions.

One last time, make sure that there are no unfilled items and put it in a brown envelope, seal it, and then along with the white envelope of your gardians, please submit it to the school in a large envelope.
